# Supplementary figures and images for: Epidemiological investigation and analysis of the genetic evolution of duck circovirus in China, 2022
Source: PLoS One. 2025 May 9;20(5):e0323282. doi: 10.1371/journal.pone.0323282 (PMC12064196; doi:10.1371/journal.pone.0323282)

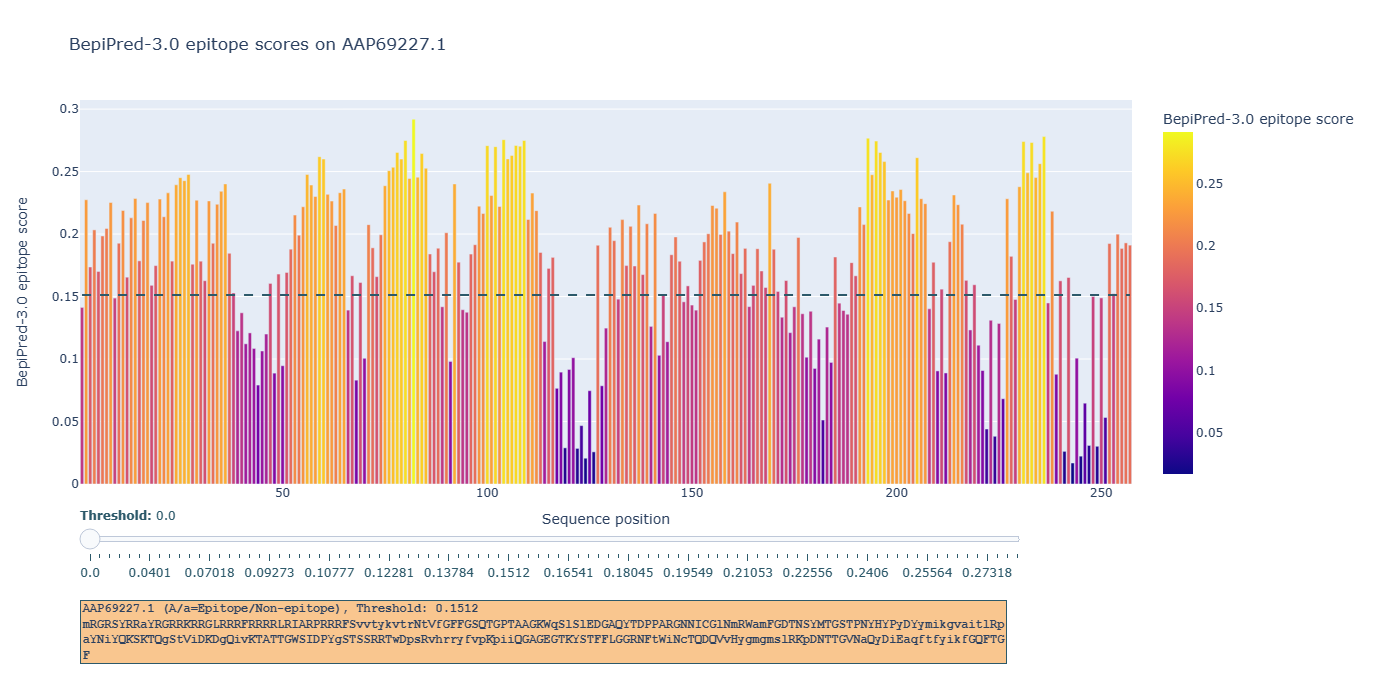

Supplement: S1 Fig — The BepiPred-3.0 online forecasting program was used with the default settings. The protein ID of the ORF2 protein of AY228555.1 is AAP69227.1. (PNG) [file pone.0323282.s001.png]
