# Supplementary material for: Epidemiological investigation and analysis of the genetic evolution of duck circovirus in China, 2022
Source: PLoS One. 2025 May 9;20(5):e0323282. doi: 10.1371/journal.pone.0323282 (PMC12064196; doi:10.1371/journal.pone.0323282)
Supplement: S1 Table — (DOCX) [file pone.0323282.s002.docx]

**Supplementary Table 1.** Sequence name and sequence coding of the genes sequenced in this study

| NCBI ID | Isolate | Abbreviation | Collection date | Country | City | Host |
| --- | --- | --- | --- | --- | --- | --- |
| OR387724 | Anhui/BB-LP0919/2022 | LP0919 | 19-Sep-22 | China | Anhui | Duck |
| OR387725 | Anhui/LB-LP1027-N3-2/2022 | LP1027-N3-2 | 27-Oct-22 | China | Anhui | Duck |
| OR387726 | Anhui/SZ-LP0720-1/2022 | LP0720-1 | 20-Jul-22 | China | Anhui | Duck |
| OR387727 | Anhui/SZ-LP0920-N2/2022 | LP0920-N2 | 20-Sep-22 | China | Anhui | Duck |
| OR387728 | Anhui/SZ-CGH0810/2022 | CGH0810 | 10-Aug-22 | China | Anhui | Duck |
| OR387729 | Anhui/SZ-LP0924/2022 | LP0924 | 24-Sep-22 | China | Anhui | Duck |
| OR387730 | Fujian/ZZ-DCJ0729-M2-1/2022 | DCJ0729-M2-1 | 29-Jul-22 | China | Fujian | Duck |
| OR387731 | Fujian/ZZ-DCJ0729-M2-2/2022 | DCJ0729-M2-2 | 29-Jul-22 | China | Fujian | Duck |
| OR387732 | Fujian/ZZ-DCJ0729-M4-2/2022 | DCJ0729-M4-2 | 29-Jul-22 | China | Fujian | Duck |
| OR387733 | Fujian/ZZ-DCJ0729-M4-4/2022 | DCJ0729-M4-4 | 29-Jul-22 | China | Fujian | Duck |
| OR387734 | Guangdong/LJ-LZH1024-38/2022 | LZH1024-38 | 24-Oct-22 | China | Guangdong | Duck |
| OR387735 | Guangdong/R20726/2022 | R20726 | 26-Jul-22 | China | Guangdong | Duck |
| OR387736 | Guangdong/YF-ZLC0927-M1/2022 | ZLC0927-M1 | 27-Sep-22 | China | Guangdong | Duck |
| OR387737 | Guangdong/ZJ-LH0720/2022 | LH0720 | 20-Jul-22 | China | Guangdong | Duck |
| OR387738 | Guangdong/ZLC0820-M2-2/2022 | ZLC0820-M2-2 | 20-Aug-22 | China | Guangdong | Duck |
| OR387739 | Guangdong/ZQ-XYF0729-B/2022 | XYF0729-B | 29-Jul-22 | China | Guangdong | Duck |
| OR387740 | Guangdong/ZQ-XYF0804-N1/2022 | XYF0804-N1 | 4-Aug-22 | China | Guangdong | Duck |
| OR387741 | Guangdong/ZQ-XYF0805-N3-2/2022 | XYF0805-N3-2 | 5-Aug-22 | China | Guangdong | Duck |
| OR387742 | Guangdong/ZQ-XYF0815/2022 | XYF0815 | 15-Aug-22 | China | Guangdong | Duck |
| OR387743 | Guangdong/ZQ-XYF0909-2/2022 | XYF0909-2 | 9-Sep-22 | China | Guangdong | Duck |
| OR387744 | Guangdong/ZQ-XYF1010-1/2022 | XYF1010-1 | 10-Oct-22 | China | Guangdong | Duck |
| OR387745 | Guangdong/ZQ-XYF1010-4/2022 | XYF1010-4 | 10-Oct-22 | China | Guangdong | Duck |
| OR387746 | Guangdong/ZQ-XYX1018-3/2022 | XYX1018-3 | 18-Oct-22 | China | Guangdong | Duck |
| OR387747 | Guangdong/ZQ-XYX1018-4/2022 | XYX1018-4 | 18-Oct-22 | China | Guangdong | Duck |
| OR387748 | Guangdong/ZQ-XYF1005-38-4/2022 | XYF1005-38-4 | 5-Oct-22 | China | Guangdong | Duck |
| OR387749 | Guangdong/ZZ-L-LH0720-2/2022 | LH0720-2 | 20-Jul-22 | China | Guangdong | Duck |
| OR387750 | Guangxi/HZ-ZCY0822/2022 | ZCY0822 | 22-Aug-22 | China | Guangxi | Duck |
| OR387751 | Guangxi/LB-YYX0921-1/2022 | YYX0921-1 | 21-Sep-22 | China | Guangxi | Duck |
| OR387752 | Henan/SQ-LBF0712-G/2022 | LBF0712-G | 12-Jul-22 | China | Henan | Duck |
| OR387753 | Henan/ZK-ZXL0810-3/2022 | ZXL0810-3 | 10-Aug-22 | China | Henan | Duck |
| OR387754 | Henan/ZZ-ZXL0810-4/2022 | ZXL0810-4 | 10-Aug-22 | China | Henan | Duck |
| OR387755 | Jiangxi/GZ-GXG0905-X4/2022 | GXG0905-X4 | 5-Sep-22 | China | Jiangxi | Duck |
| OR387756 | Jiangxi/GZ-GXG0919/2022 | GXG0919 | 19-Sep-22 | China | Jiangxi | Duck |
| OR387757 | Shandong/DZ-WJB0823-G4/2022 | WJB0823-G4 | 23-Aug-22 | China | Shandong | Duck |
| OR387758 | Shandong/DZ-WJB0823-Y2/2022 | WJB0823-Y2 | 23-Aug-22 | China | Shandong | Duck |
| OR387759 | Shandong/DZ-XGM0718-B/2022 | XGM0718-B | 18-Jul-22 | China | Shandong | Duck |
| OR387760 | Shandong/DZ-XGM0922-M1-3/2022 | XGM0922-M1-3 | 22-Sep-22 | China | Shandong | Duck |
| OR387761 | Shandong/DZ-XGM0922-M1-4/2022 | XGM0922-M1-4 | 22-Sep-22 | China | Shandong | Duck |
| OR387762 | Shandong/LC-LF0720-2/2022 | LF0720-2 | 20-Jul-22 | China | Shandong | Duck |
| OR387763 | Shandong/LC-LF0815/2022 | LF0815 | 15-Aug-22 | China | Shandong | Duck |
| OR387764 | Shandong/LC-XCY0820GYZ/2022 | XCY0820GYZ | 20-Aug-22 | China | Shandong | Duck |
| OR387765 | Shandong/LC-XCY0820SJJ/2022 | XCY0820SJJ | 20-Aug-22 | China | Shandong | Duck |
| OR387766 | Shandong/LY-GZC0830/2022 | GZC0830 | 30-Aug-22 | China | Shandong | Duck |
| OR387767 | Shandong/TA-WT1024-N/2022 | WT1024-N | 24-Oct-22 | China | Shandong | Duck |
| OR387768 | Shandong/WF-C0720-G7-2/2022 | C0720-G7-2 | 20-Jul-22 | China | Shandong | Duck |
| OR387769 | Shandong/WF-C0720-G7-3/2022 | C0720-G7-3 | 20-Jul-22 | China | Shandong | Duck |
| OR387770 | Shandong/WF-C0720-Y7-2/2022 | C0720-Y7-2 | 20-Jul-22 | China | Shandong | Duck |
| OR387771 | Shandong/WF-CXZ0721-G6-1/2022 | CXZ0721-G6-1 | 21-Jul-22 | China | Shandong | Duck |
| OR387772 | Shandong/WF-CXZ0721-G6-2/2022 | CXZ0721-G6-2 | 21-Jul-22 | China | Shandong | Duck |
| OR387773 | Shandong/WF-FXR0819/2022 | FXR0819 | 19-Aug-22 | China | Shandong | Duck |
| OR387774 | Sichuan/CD-FDD0930/2022 | FDD0930 | 30-Sep-22 | China | Sichuan | Duck |
