# Supplementary material for: Epidemiological investigation and analysis of the genetic evolution of duck circovirus in China, 2022
Source: PLoS One. 2025 May 9;20(5):e0323282. doi: 10.1371/journal.pone.0323282 (PMC12064196; doi:10.1371/journal.pone.0323282)
Supplement: S2 Table — (DOCX) [file pone.0323282.s003.docx]

**S2 Table. Summary of sample information for this study**

| Sample collection date | Province | Species | Breed | Day of age | No of samples tested |
| --- | --- | --- | --- | --- | --- |
| 2022.01.04 | Guangdong | Duck | Muscovy Duck | 35 | 1 |
| 2022.01.04 | Guangdong | Duck | White Duck | 25 | 1 |
| 2022.01.04 | Guangdong | Duck | White Duck | 30 | 1 |
| 2022.01.04 | Shandong | Duck | / | 13 | 1 |
| 2022.01.04 | Shandong | Duck | / | 13 | 1 |
| 2022.01.04 | Shandong | Duck | / | 13 | 1 |
| 2022.01.04 | Shandong | Duck | / | 7 | 1 |
| 2022.01.04 | Shandong | Duck | / | 20 | 1 |
| 2022.01.04 | Shandong | Duck | / | 20 | 1 |
| 2022.01.04 | Shandong | Duck | / | 20 | 1 |
| 2022.01.04 | Hebei | Duck | / | 30 | 1 |
| 2022.01.06 | Guangdong | Duck | White Duck | 35 | 1 |
| 2022.01.06 | Guangdong | Duck | White Duck | 35 | 1 |
| 2022.01.06 | Guangdong | Duck | White Duck | 35 | 1 |
| 2022.01.06 | Guangdong | Duck | White Duck | 16 | 1 |
| 2022.01.06 | Guangdong | Duck | White Duck | 16 | 1 |
| 2022.01.06 | Guangdong | Duck | White Duck | 16 | 1 |
| 2022.01.06 | Guangdong | Duck | White Duck | 31 | 1 |
| 2022.01.06 | Anhui | Duck | / | 21 | 1 |
| 2022.01.06 | Shandong | Duck | / | 26 | 1 |
| 2022.01.06 | Shandong | Duck | / | 26 | 1 |
| 2022.01.07 | Anhui | Duck | Muscovy Duck | 11 | 1 |
| 2022.01.07 | Anhui | Duck | Muscovy Duck | 11 | 1 |
| 2022.01.07 | Anhui | Duck | Muscovy Duck | 11 | 1 |
| 2022.01.07 | Anhui | Duck | Muscovy Duck | 11 | 1 |
| 2022.01.07 | Anhui | Duck | Muscovy Duck | 11 | 1 |
| 2022.01.07 | Anhui | Duck | Muscovy Duck | 11 | 1 |
| 2022.01.07 | Anhui | Duck | Muscovy Duck | 11 | 1 |
| 2022.01.07 | Anhui | Duck | Muscovy Duck | 11 | 1 |
| 2022.01.07 | Anhui | Duck | Muscovy Duck | 11 | 1 |
| 2022.01.07 | Anhui | Duck | Muscovy Duck | 11 | 1 |
| 2022.01.07 | Anhui | Duck | Muscovy Duck | 11 | 1 |
| 2022.01.07 | Anhui | Duck | Muscovy Duck | 11 | 1 |
| 2022.01.07 | Hebei | Duck | Cherry Valley Duck | 25 | 1 |
| 2022.01.07 | Hebei | Duck | Cherry Valley Duck | 25 | 1 |
| 2022.01.07 | Hebei | Duck | Cherry Valley Duck | 25 | 1 |
| 2022.01.07 | Nei Mongol | Duck | / | 20 | 1 |
| 2022.01.07 | Nei Mongol | Duck | / | 20 | 1 |
| 2022.01.07 | Shanxi | Duck | Cherry Valley Duck | 1 | 1 |
| 2022.01.07 | Shanxi | Duck | Cherry Valley Duck | 1 | 1 |
| 2022.01.07 | Shanxi | Duck | Cherry Valley Duck | 1 | 1 |
| 2022.01.07 | Shanxi | Duck | Cherry Valley Duck | 1 | 1 |
| 2022.01.07 | Shanxi | Duck | Cherry Valley Duck | 1 | 1 |
| 2022.01.07 | Shanxi | Duck | Cherry Valley Duck | 1 | 1 |
| 2022.01.08 | Guangdong | Duck | Shelduck | 40 | 1 |
| 2022.01.08 | Guangdong | Duck | Shelduck | 40 | 1 |
| 2022.01.08 | Guangdong | Duck | White Muscovy Duck | 31 | 1 |
| 2022.01.08 | Anhui | Duck | / | 34 | 1 |
| 2022.01.08 | Anhui | Duck | / | 34 | 1 |
| 2022.01.08 | Anhui | Duck | / | 34 | 1 |
| 2022.01.08 | Hebei | Duck | White Duck | 26 | 1 |
| 2022.01.08 | Hebei | Duck | White Duck | 26 | 1 |
| 2022.01.08 | Shandong | Duck | / | 27 | 1 |
| 2022.01.08 | Shandong | Duck | / | 32 | 1 |
| 2022.01.10 | Anhui | Duck | / | 31 | 1 |
| 2022.01.10 | Guangdong | Duck | Muscovy Duck | 45 | 1 |
| 2022.01.10 | Guangdong | Duck | Muscovy Duck | 45 | 1 |
| 2022.01.10 | Hebei | Duck | Cherry Valley Duck | 27 | 1 |
| 2022.01.11 | Anhui | Duck | / | 29 | 1 |
| 2022.01.11 | Anhui | Duck | / | 29 | 1 |
| 2022.01.11 | Shandong | Duck | / | 26 | 1 |
| 2022.01.11 | Shandong | Duck | / | 26 | 1 |
| 2022.01.11 | Shandong | Duck | / | 32 | 1 |
| 2022.01.12 | Guangdong | Duck | Slightly Black Duck | 65 | 1 |
| 2022.01.12 | Guangdong | Duck | Shelduck | 35 | 1 |
| 2022.01.12 | Guangdong | Duck | Shelduck | 35 | 1 |
| 2022.01.12 | Guangdong | Duck | Shelduck | 35 | 1 |
| 2022.01.12 | Guangdong | Duck | Shelduck | 35 | 1 |
| 2022.01.12 | Guangdong | Duck | Shelduck | 35 | 1 |
| 2022.01.12 | Guangdong | Duck | Shelduck | 35 | 1 |
| 2022.01.12 | Guangdong | Duck | Shelduck | 30 | 1 |
| 2022.01.12 | Guangdong | Duck | Shelduck | 30 | 1 |
| 2022.01.12 | Guangdong | Duck | Shelduck | 30 | 1 |
| 2022.01.12 | Anhui | Duck | / | 34 | 1 |
| 2022.01.13 | Sichuan | Duck | / | 32 | 1 |
| 2022.01.14 | Guangxi | Duck | / | 240 | 1 |
| 2022.01.14 | Guangxi | Duck | / | 28 | 1 |
| 2022.01.14 | Guangxi | Duck | / | 28 | 1 |
| 2022.01.14 | Guangxi | Duck | / | 26 | 1 |
| 2022.01.14 | Guangxi | Duck | / | 26 | 1 |
| 2022.01.14 | Jiangsu | Duck | White Duck | 10 | 1 |
| 2022.01.14 | Hebei | Duck | / | 25 | 1 |
| 2022.01.14 | Shandong | Duck | / | 12 | 1 |
| 2022.01.14 | Shandong | Duck | / | 13 | 1 |
| 2022.01.14 | Shandong | Duck | / | 15 | 1 |
| 2022.01.14 | Shandong | Duck | / | 35 | 1 |
| 2022.01.14 | Shandong | Duck | / | 35 | 1 |
| 2022.01.14 | Shandong | Duck | / | 33 | 1 |
| 2022.01.17 | Anhui | Duck | Cherry Valley Duck | 28 | 1 |
| 2022.01.17 | Anhui | Duck | Cherry Valley Duck | 28 | 1 |
| 2022.01.17 | Anhui | Duck | Cherry Valley Duck | 28 | 1 |
| 2022.01.17 | Anhui | Duck | Cherry Valley Duck | 28 | 1 |
| 2022.01.17 | Guangdong | Duck | White Duck | 8 | 1 |
| 2022.01.17 | Guangdong | Duck | White Duck | 8 | 1 |
| 2022.01.17 | Guangdong | Duck | White Duck | 8 | 1 |
| 2022.01.17 | Guangxi | Duck | Muscovy Duck | 23 | 1 |
| 2022.01.17 | Shandong | Duck | / | 30 | 1 |
| 2022.01.17 | Shandong | Duck | / | 35 | 1 |
| 2022.01.17 | Shandong | Duck | / | 26 | 1 |
| 2022.01.17 | Henan | Duck | Cherry Valley Duck | 30 | 1 |
| 2022.01.18 | Guangdong | Duck | White Duck | 37 | 1 |
| 2022.01.18 | Nei Mongol | Duck | / | 34 | 1 |
| 2022.01.20 | Sichuan | Duck | / | 150 | 1 |
| 2022.01.21 | Nei Mongol | Duck | / | 5 | 1 |
| 2022.01.22 | Fujian | Duck | Muscovy Duck | 40 | 1 |
| 2022.01.22 | Fujian | Duck | Muscovy Duck | 40 | 1 |
| 2022.01.22 | Fujian | Duck | Muscovy Duck | 40 | 1 |
| 2022.01.22 | Fujian | Duck | Muscovy Duck | 40 | 1 |
| 2022.01.22 | Fujian | Duck | Muscovy Duck | 24 | 1 |
| 2022.01.22 | Fujian | Duck | Muscovy Duck | 24 | 1 |
| 2022.01.22 | Fujian | Duck | Muscovy Duck | 20 | 1 |
| 2022.01.22 | Fujian | Duck | Muscovy Duck | 20 | 1 |
| 2022.01.22 | Fujian | Duck | Muscovy Duck | 20 | 1 |
| 2022.01.22 | Fujian | Duck | Muscovy Duck | 20 | 1 |
| 2022.01.22 | Fujian | Duck | Muscovy Duck | 31 | 1 |
| 2022.01.22 | Fujian | Duck | Muscovy Duck | 31 | 1 |
| 2022.01.22 | Fujian | Duck | Muscovy Duck | 46 | 1 |
| 2022.01.22 | Fujian | Duck | Muscovy Duck | 46 | 1 |
| 2022.01.22 | Fujian | Duck | Muscovy Duck | 63 | 1 |
| 2022.01.22 | Fujian | Duck | Muscovy Duck | 63 | 1 |
| 2022.01.22 | Fujian | Duck | Muscovy Duck | 83 | 1 |
| 2022.01.22 | Fujian | Duck | Muscovy Duck | 83 | 1 |
| 2022.01.22 | Fujian | Duck | Muscovy Duck | 298 | 1 |
| 2022.01.22 | Fujian | Duck | Muscovy Duck | 298 | 1 |
| 2022.01.22 | Fujian | Duck | Muscovy Duck | 180 | 1 |
| 2022.01.22 | Fujian | Duck | Muscovy Duck | 180 | 1 |
| 2022.01.22 | Fujian | Duck | Muscovy Duck | 133 | 1 |
| 2022.01.22 | Fujian | Duck | Muscovy Duck | 133 | 1 |
| 2022.01.22 | Hebei | Duck | / | 1 | 1 |
| 2022.01.24 | Guangdong | Duck | Mule Duck | 6 | 1 |
| 2022.01.24 | Guangdong | Duck | Muscovy Duck | 12 | 1 |
| 2022.01.25 | Anhui | Duck | Muscovy Duck | 40 | 1 |
| 2022.01.25 | Anhui | Duck | Muscovy Duck | 25 | 1 |
| 2022.01.25 | Anhui | Duck | Muscovy Duck | 25 | 1 |
| 2022.01.25 | Anhui | Duck | Muscovy Duck | 40 | 1 |
| 2022.01.25 | Shandong | Duck | / | 1 | 1 |
| 2022.01.25 | Shandong | Duck | / | 1 | 1 |
| 2022.02.09 | Guangxi | Duck | / | 350 | 1 |
| 2022.02.09 | Anhui | Duck | / | / | 1 |
| 2022.02.09 | Anhui | Duck | / | / | 1 |
| 2022.02.09 | Anhui | Duck | / | / | 1 |
| 2022.02.09 | Anhui | Duck | / | / | 1 |
| 2022.02.09 | Anhui | Duck | / | / | 1 |
| 2022.02.09 | Anhui | Duck | / | / | 1 |
| 2022.02.09 | Anhui | Duck | / | / | 1 |
| 2022.02.09 | Heilongjiang | Duck | / | 30 | 1 |
| 2022.02.09 | Shandong | Duck | / | 30 | 1 |
| 2022.02.09 | Shandong | Duck | / | 31 | 1 |
| 2022.02.14 | Henan | Duck | / | 58 | 1 |
| 2022.02.14 | Shandong | Duck | / | 28 | 1 |
| 2022.02.14 | Shandong | Duck | / | 28 | 1 |
| 2022.02.14 | Shandong | Duck | / | / | 1 |
| 2022.02.14 | Shandong | Duck | / | / | 1 |
| 2022.02.14 | Shandong | Duck | / | / | 1 |
| 2022.02.14 | Shandong | Duck | / | / | 1 |
| 2022.02.14 | Shandong | Duck | / | / | 1 |
| 2022.02.14 | Shandong | Duck | / | / | 1 |
| 2022.02.14 | Shandong | Duck | / | / | 1 |
| 2022.02.14 | Shandong | Duck | / | / | 1 |
| 2022.02.14 | Shandong | Duck | / | / | 1 |
| 2022.02.14 | Shandong | Duck | / | / | 1 |
| 2022.02.14 | Shandong | Duck | / | / | 1 |
| 2022.02.14 | Shandong | Duck | / | / | 1 |
| 2022.02.14 | Shandong | Duck | / | / | 1 |
| 2022.02.14 | Shandong | Duck | / | / | 1 |
| 2022.02.14 | Shandong | Duck | / | / | 1 |
| 2022.02.14 | Shandong | Duck | / | / | 1 |
| 2022.02.14 | Shandong | Duck | / | / | 1 |
| 2022.02.14 | Shandong | Duck | / | / | 1 |
| 2022.02.14 | Shandong | Duck | / | / | 1 |
| 2022.02.14 | Shandong | Duck | / | / | 1 |
| 2022.02.14 | Shandong | Duck | / | / | 1 |
| 2022.02.14 | Shandong | Duck | / | / | 1 |
| 2022.02.14 | Shandong | Duck | / | / | 1 |
| 2022.02.14 | Shandong | Duck | / | / | 1 |
| 2022.02.14 | Shandong | Duck | / | / | 1 |
| 2022.02.14 | Shandong | Duck | / | / | 1 |
| 2022.02.14 | Shandong | Duck | / | / | 1 |
| 2022.02.14 | Shandong | Duck | / | / | 1 |
| 2022.02.14 | Shandong | Duck | / | / | 1 |
| 2022.02.14 | Shandong | Duck | / | / | 1 |
| 2022.02.14 | Shandong | Duck | / | / | 1 |
| 2022.02.14 | Shandong | Duck | / | / | 1 |
| 2022.02.14 | Shandong | Duck | / | / | 1 |
| 2022.02.14 | Shandong | Duck | / | / | 1 |
| 2022.02.14 | Shandong | Duck | / | / | 1 |
| 2022.02.14 | Shandong | Duck | / | / | 1 |
| 2022.02.14 | Shandong | Duck | / | / | 1 |
| 2022.02.14 | Shandong | Duck | / | / | 1 |
| 2022.02.14 | Shandong | Duck | / | / | 1 |
| 2022.02.14 | Shandong | Duck | / | / | 1 |
| 2022.02.14 | Shandong | Duck | / | / | 1 |
| 2022.02.14 | Shandong | Duck | / | / | 1 |
| 2022.02.14 | Henan | Duck | Cherry Valley Duck | 18 | 1 |
| 2022.02.17 | Shannxi | Duck | White Duck | 24 | 1 |
| 2022.02.17 | Shannxi | Duck | White Duck | 24 | 1 |
| 2022.02.17 | Shannxi | Duck | White Duck | 24 | 1 |
| 2022.02.17 | Shannxi | Duck | White Duck | 24 | 1 |
| 2022.02.17 | Shannxi | Duck | White Duck | 24 | 1 |
| 2022.02.17 | Shannxi | Duck | White Duck | 24 | 1 |
| 2022.02.17 | Shannxi | Duck | White Duck | 24 | 1 |
| 2022.02.17 | Shannxi | Duck | White Duck | 24 | 1 |
| 2022.02.17 | Shandong | Duck | / | 17 | 1 |
| 2022.02.17 | Shandong | Duck | / | 32 | 1 |
| 2022.02.17 | Shandong | Duck | / | 28 | 1 |
| 2022.02.17 | Henan | Duck | / | 18 | 1 |
| 2022.02.17 | Shandong | Duck | / | 28 | 1 |
| 2022.02.18 | Anhui | Duck | Cherry Valley Duck | 28 | 1 |
| 2022.02.18 | Anhui | Duck | Cherry Valley Duck | 28 | 1 |
| 2022.02.18 | Anhui | Duck | / | 20 | 1 |
| 2022.02.18 | Shanxi | Duck | Cherry Valley Duck | 2 | 1 |
| 2022.02.18 | Shanxi | Duck | Cherry Valley Duck | 2 | 1 |
| 2022.02.18 | Shanxi | Duck | Cherry Valley Duck | 2 | 1 |
| 2022.02.18 | Shanxi | Duck | Cherry Valley Duck | 2 | 1 |
| 2022.02.19 | Jiangsu | Duck | / | 35 | 1 |
| 2022.02.19 | Shandong | Duck | White Duck | 20 | 1 |
| 2022.02.19 | Shandong | Duck | White Duck | 20 | 1 |
| 2022.02.19 | Shandong | Duck | White Duck | 20 | 1 |
| 2022.02.19 | Shandong | Duck | White Duck | 20 | 1 |
| 2022.02.19 | Shandong | Duck | White Duck | 20 | 1 |
| 2022.02.19 | Shandong | Duck | White Duck | 20 | 1 |
| 2022.02.19 | Shandong | Duck | White Duck | 20 | 1 |
| 2022.02.19 | Shandong | Duck | White Duck | 20 | 1 |
| 2022.02.19 | Shandong | Duck | White Duck | 20 | 1 |
| 2022.02.19 | Shandong | Duck | / | 31 | 1 |
| 2022.02.19 | Hebei | Duck | Cherry Valley Duck | 13 | 1 |
| 2022.02.19 | Henan | Duck | / | 48 | 1 |
| 2022.02.19 | Henan | Duck | / | 48 | 1 |
| 2022.02.19 | Henan | Duck | / | 48 | 1 |
| 2022.02.19 | Henan | Duck | / | 48 | 1 |
| 2022.02.20 | Fujian | Duck | Muscovy Duck | 35 | 1 |
| 2022.02.20 | Fujian | Duck | Muscovy Duck | 35 | 1 |
| 2022.02.20 | Fujian | Duck | Muscovy Duck | 50 | 1 |
| 2022.02.20 | Fujian | Duck | Muscovy Duck | 50 | 1 |
| 2022.02.20 | Fujian | Duck | Muscovy Duck | 50 | 1 |
| 2022.02.20 | Fujian | Duck | Muscovy Duck | 50 | 1 |
| 2022.02.20 | Fujian | Duck | Muscovy Duck | 50 | 1 |
| 2022.02.20 | Fujian | Duck | Muscovy Duck | 50 | 1 |
| 2022.02.20 | Fujian | Duck | Muscovy Duck | 50 | 1 |
| 2022.02.20 | Fujian | Duck | Muscovy Duck | 50 | 1 |
| 2022.02.20 | Fujian | Duck | Muscovy Duck | 50 | 1 |
| 2022.02.20 | Fujian | Duck | Muscovy Duck | 50 | 1 |
| 2022.02.20 | Fujian | Duck | Muscovy Duck | 100 | 1 |
| 2022.02.20 | Fujian | Duck | Muscovy Duck | 100 | 1 |
| 2022.02.20 | Fujian | Duck | Muscovy Duck | 100 | 1 |
| 2022.02.20 | Fujian | Duck | Muscovy Duck | 100 | 1 |
| 2022.02.20 | Fujian | Duck | Muscovy Duck | 100 | 1 |
| 2022.02.20 | Fujian | Duck | Muscovy Duck | 100 | 1 |
| 2022.02.20 | Fujian | Duck | Muscovy Duck | 100 | 1 |
| 2022.02.20 | Fujian | Duck | Muscovy Duck | 100 | 1 |
| 2022.02.20 | Fujian | Duck | Muscovy Duck | 100 | 1 |
| 2022.02.20 | Fujian | Duck | Muscovy Duck | 100 | 1 |
| 2022.02.20 | Fujian | Duck | Muscovy Duck | 100 | 1 |
| 2022.02.20 | Fujian | Duck | Muscovy Duck | 100 | 1 |
| 2022.02.22 | Anhui | Duck | Muscovy Duck | 101 | 1 |
| 2022.02.22 | Anhui | Duck | Muscovy Duck | 171 | 1 |
| 2022.02.22 | Anhui | Duck | Muscovy Duck | 440 | 1 |
| 2022.02.22 | Guangdong | Duck | White Duck | 11 | 1 |
| 2022.02.22 | Guangdong | Duck | White Duck | 11 | 1 |
| 2022.02.22 | Guangdong | Duck | White Duck | 11 | 1 |
| 2022.02.22 | Guangdong | Duck | White Duck | 11 | 1 |
| 2022.02.22 | Henan | Duck | / | 30 | 1 |
| 2022.02.22 | Anhui | Duck | / | 40 | 1 |
| 2022.02.22 | Shandong | Duck | Cherry Valley Duck | 11 | 1 |
| 2022.02.22 | Hebei | Duck | / | 42 | 1 |
| 2022.02.22 | Shandong | Duck | / | 14 | 1 |
| 2022.02.22 | Shandong | Duck | / | 14 | 1 |
| 2022.02.22 | Shandong | Duck | / | 14 | 1 |
| 2022.02.22 | Shandong | Duck | / | 22 | 1 |
| 2022.02.22 | Shandong | Duck | Cherry Valley Duck | 23 | 1 |
| 2022.02.22 | Shandong | Duck | Cherry Valley Duck | 23 | 1 |
| 2022.02.22 | Shandong | Duck | Cherry Valley Duck | 23 | 1 |
| 2022.02.22 | Henan | Duck | Cherry Valley Duck | 9 | 1 |
| 2022.02.22 | Henan | Duck | Cherry Valley Duck | 9 | 1 |
| 2022.02.22 | Henan | Duck | Cherry Valley Duck | 9 | 1 |
| 2022.02.23 | Anhui | Duck | / | 30 | 1 |
| 2022.02.23 | Shandong | Duck | / | 14 | 1 |
| 2022.02.23 | Shandong | Duck | Cherry Valley Duck | 26 | 1 |
| 2022.02.23 | Shandong | Duck | Cherry Valley Duck | 26 | 1 |
| 2022.02.23 | Hebei | Duck | / | 1 | 1 |
| 2022.02.23 | Hebei | Duck | / | 1 | 1 |
| 2022.02.23 | Hebei | Duck | / | 1 | 1 |
| 2022.02.23 | Hebei | Duck | / | 1 | 1 |
| 2022.02.24 | Sichuan | Duck | / | 27 | 1 |
| 2022.02.24 | Sichuan | Duck | / | 27 | 1 |
| 2022.02.24 | Sichuan | Duck | / | 27 | 1 |
| 2022.02.24 | Guangdong | Duck | White Duck | 16 | 1 |
| 2022.02.24 | Shandong | Duck | Cherry Valley Duck | 25 | 1 |
| 2022.02.26 | Guangdong | Duck | Shelduck | 13 | 1 |
| 2022.02.26 | Guangdong | Duck | Shelduck | 15 | 1 |
| 2022.02.26 | Anhui | Duck | Muscovy Duck | 184 | 1 |
| 2022.02.26 | Anhui | Duck | Muscovy Duck | 184 | 1 |
| 2022.02.26 | Shandong | Duck | / | 29 | 1 |
| 2022.02.26 | Shandong | Duck | / | 29 | 1 |
| 2022.02.28 | Guangdong | Duck | / | / | 1 |
| 2022.02.28 | Guangdong | Duck | / | / | 1 |
| 2022.02.28 | Guangdong | Duck | / | / | 1 |
| 2022.02.28 | Guangdong | Duck | / | / | 1 |
| 2022.02.28 | Shandong | Duck | / | 32 | 1 |
| 2022.02.28 | Shandong | Duck | / | 32 | 1 |
| 2022.02.28 | Shandong | Duck | / | 32 | 1 |
| 2022.02.28 | Shandong | Duck | / | 32 | 1 |
| 2022.02.28 | Shandong | Duck | / | 32 | 1 |
| 2022.02.28 | Shandong | Duck | / | 9 | 1 |
| 2022.02.28 | Shandong | Duck | / | 29 | 1 |
| 2022.03.01 | Anhui | Duck | / | 12 | 1 |
| 2022.03.01 | Guangdong | Duck | White Duck | 37 | 1 |
| 2022.03.01 | Guangdong | Duck | Muscovy Duck | 13 | 1 |
| 2022.03.01 | Anhui | Duck | / | 27 | 1 |
| 2022.03.02 | Guangdong | Duck | Muscovy Duck | 25 | 1 |
| 2022.03.02 | Anhui | Duck | / | 20 | 1 |
| 2022.03.02 | Hebei | Duck | / | / | 1 |
| 2022.03.02 | Hebei | Duck | / | / | 1 |
| 2022.03.02 | Hebei | Duck | / | / | 1 |
| 2022.03.02 | Hebei | Duck | / | / | 1 |
| 2022.03.03 | Shandong | Duck | / | 34 | 1 |
| 2022.03.04 | Hebei | Duck | Cherry Valley Duck | 33 | 1 |
| 2022.03.09 | Anhui | Duck | / | 35 | 1 |
| 2022.03.09 | Guangdong | Duck | Shelduck | 35 | 1 |
| 2022.03.09 | Guangdong | Duck | Muscovy Duck | 35 | 1 |
| 2022.03.09 | Jiangsu | Duck | / | 20 | 1 |
| 2022.03.09 | Shandong | Duck | / | 28 | 1 |
| 2022.03.09 | Shandong | Duck | / | 29 | 1 |
| 2022.03.09 | Shandong | Duck | / | 29 | 1 |
| 2022.03.09 | Shandong | Duck | / | 29 | 1 |
| 2022.03.09 | Shandong | Duck | / | 29 | 1 |
| 2022.03.09 | Shandong | Duck | / | 29 | 1 |
| 2022.03.09 | Shandong | Duck | / | 29 | 1 |
| 2022.03.09 | Shandong | Duck | / | 29 | 1 |
| 2022.03.09 | Shandong | Duck | / | 29 | 1 |
| 2022.03.09 | Shandong | Duck | / | 29 | 1 |
| 2022.03.09 | Shandong | Duck | / | 29 | 1 |
| 2022.03.09 | Shandong | Duck | / | 29 | 1 |
| 2022.03.09 | Shandong | Duck | / | 29 | 1 |
| 2022.03.09 | Shandong | Duck | / | 29 | 1 |
| 2022.03.09 | Shandong | Duck | / | 29 | 1 |
| 2022.03.10 | Fujian | Duck | Mule Duck | 70 | 1 |
| 2022.03.10 | Fujian | Duck | Mule Duck | 40 | 1 |
| 2022.03.10 | Guangdong | Duck | Muscovy Duck | 60 | 1 |
| 2022.03.10 | Hebei | Duck | Cherry Valley Duck | 1 | 1 |
| 2022.03.10 | Shandong | Duck | / | 9 | 1 |
| 2022.03.10 | Shandong | Duck | / | 9 | 1 |
| 2022.03.11 | Anhui | Duck | / | 19 | 1 |
| 2022.03.14 | Guangdong | Duck | Shelduck | 32 | 1 |
| 2022.03.14 | Guangdong | Duck | Shelduck | 32 | 1 |
| 2022.03.14 | Anhui | Duck | / | 34 | 1 |
| 2022.03.14 | Anhui | Duck | / | 132 | 1 |
| 2022.03.14 | Anhui | Duck | / | 132 | 1 |
| 2022.03.14 | Anhui | Duck | / | 132 | 1 |
| 2022.03.14 | Anhui | Duck | / | 132 | 1 |
| 2022.03.14 | Anhui | Duck | / | 132 | 1 |
| 2022.03.14 | Anhui | Duck | / | 132 | 1 |
| 2022.03.14 | Anhui | Duck | / | 132 | 1 |
| 2022.03.14 | Anhui | Duck | / | 132 | 1 |
| 2022.03.14 | Shandong | Duck | / | 27 | 1 |
| 2022.03.15 | Guangxi | Duck | / | 22 | 1 |
| 2022.03.15 | Guangxi | Duck | / | 22 | 1 |
| 2022.03.15 | Guangxi | Duck | / | 22 | 1 |
| 2022.03.15 | Guangxi | Duck | / | 22 | 1 |
| 2022.03.15 | Guangxi | Duck | / | 22 | 1 |
| 2022.03.15 | Guangxi | Duck | / | 22 | 1 |
| 2022.03.15 | Guangxi | Duck | / | 22 | 1 |
| 2022.03.15 | Guangdong | Duck | White Duck | 25 | 1 |
| 2022.03.15 | Guangdong | Duck | White Duck | 29 | 1 |
| 2022.03.15 | Shandong | Duck | / | 31 | 1 |
| 2022.03.15 | Henan | Duck | / | 33 | 1 |
| 2022.03.15 | Henan | Duck | / | 33 | 1 |
| 2022.03.21 | Guangdong | Duck | / | 55 | 1 |
| 2022.03.25 | Guangdong | Duck | / | 30 | 1 |
| 2022.03.25 | Guangdong | Duck | Muscovy Duck | 50 | 1 |
| 2022.03.25 | Guangdong | Duck | Muscovy Duck | 70 | 1 |
| 2022.03.25 | Hebei | Duck | / | 20 | 1 |
| 2022.03.25 | Hebei | Duck | / | 20 | 1 |
| 2022.03.28 | Jiangxi | Duck | / | 35 | 1 |
| 2022.03.28 | Jiangxi | Duck | / | 35 | 1 |
| 2022.03.28 | Jiangxi | Duck | / | 35 | 1 |
| 2022.03.28 | Jiangxi | Duck | / | 35 | 1 |
| 2022.03.28 | Jiangxi | Duck | / | 35 | 1 |
| 2022.03.28 | Jiangxi | Duck | / | 35 | 1 |
| 2022.03.28 | Jiangxi | Duck | / | 35 | 1 |
| 2022.03.28 | Jiangxi | Duck | / | 35 | 1 |
| 2022.03.28 | Jiangxi | Duck | / | 35 | 1 |
| 2022.03.29 | Shandong | Duck | / | 24 | 1 |
| 2022.03.29 | Shandong | Duck | / | 24 | 1 |
| 2022.03.31 | Shandong | Duck | / | 29 | 1 |
| 2022.03.31 | Shandong | Duck | / | 30 | 1 |
| 2022.03.31 | Shandong | Duck | / | / | 1 |
| 2022.03.31 | Shandong | Duck | / | / | 1 |
| 2022.03.31 | Shandong | Duck | / | / | 1 |
| 2022.03.31 | Shandong | Duck | / | / | 1 |
| 2022.03.31 | Shandong | Duck | / | / | 1 |
| 2022.03.31 | Shandong | Duck | / | / | 1 |
| 2022.03.31 | Shandong | Duck | / | / | 1 |
| 2022.03.31 | Shandong | Duck | / | / | 1 |
| 2022.03.31 | Shandong | Duck | / | / | 1 |
| 2022.03.31 | Shandong | Duck | / | / | 1 |
| 2022.03.31 | Shandong | Duck | / | / | 1 |
| 2022.03.31 | Shandong | Duck | / | / | 1 |
| 2022.03.31 | Shandong | Duck | / | / | 1 |
| 2022.03.31 | Shandong | Duck | / | / | 1 |
| 2022.03.31 | Shandong | Duck | / | / | 1 |
| 2022.03.31 | Shandong | Duck | / | / | 1 |
| 2022.04.01 | Shandong | Duck | Cherry Valley Duck | 33 | 1 |
| 2022.04.01 | Shandong | Duck | Cherry Valley Duck | 33 | 1 |
| 2022.04.01 | Shandong | Duck | Cherry Valley Duck | 33 | 1 |
| 2022.04.01 | Shandong | Duck | Cherry Valley Duck | 33 | 1 |
| 2022.04.06 | Shandong | Duck | White Duck | 12 | 1 |
| 2022.04.08 | Jiangxi | Duck | White Duck | 27 | 1 |
| 2022.04.11 | Fujian | Duck | Mule Duck | 36 | 1 |
| 2022.04.11 | Fujian | Duck | Mule Duck | 36 | 1 |
| 2022.04.11 | Fujian | Duck | Mule Duck | 36 | 1 |
| 2022.04.11 | Fujian | Duck | Mule Duck | 36 | 1 |
| 2022.04.11 | Guangdong | Duck | Muscovy Duck | 200 | 1 |
| 2022.04.11 | Guangdong | Duck | White Duck | 15 | 1 |
| 2022.04.11 | Guangdong | Duck | White Duck | 15 | 1 |
| 2022.04.11 | Guangdong | Duck | White Duck | 46 | 1 |
| 2022.04.11 | Guangdong | Duck | Muscovy Duck | 12 | 1 |
| 2022.04.11 | Guangdong | Duck | Muscovy Duck | 12 | 1 |
| 2022.04.11 | Shandong | Duck | / | 13 | 1 |
| 2022.04.11 | Shandong | Duck | / | 13 | 1 |
| 2022.04.11 | Shandong | Duck | / | 13 | 1 |
| 2022.04.11 | Shandong | Duck | / | 13 | 1 |
| 2022.04.11 | Shandong | Duck | / | 13 | 1 |
| 2022.04.11 | Shandong | Duck | / | 13 | 1 |
| 2022.04.15 | Shandong | Duck | / | 15 | 1 |
| 2022.04.15 | Shandong | Duck | / | 15 | 1 |
| 2022.04.16 | Shandong | Duck | White Duck | 23 | 1 |
| 2022.04.16 | Shandong | Duck | White Duck | 33 | 1 |
| 2022.04.16 | Shandong | Duck | White Duck | 37 | 1 |
| 2022.04.16 | Shandong | Duck | White Duck | 37 | 1 |
| 2022.04.16 | Shandong | Duck | White Duck | 37 | 1 |
| 2022.04.16 | Shandong | Duck | White Duck | 37 | 1 |
| 2022.04.18 | Shandong | Duck | / | 24 | 1 |
| 2022.04.18 | Shandong | Duck | / | 24 | 1 |
| 2022.04.18 | Shandong | Duck | / | 35 | 1 |
| 2022.04.20 | Guangdong | Duck | / | 180 | 1 |
| 2022.04.20 | Guangdong | Duck | / | 180 | 1 |
| 2022.04.20 | Guangdong | Duck | / | 180 | 1 |
| 2022.04.20 | Guangdong | Duck | / | 180 | 1 |
| 2022.04.20 | Guangdong | Duck | / | 180 | 1 |
| 2022.04.20 | Guangdong | Duck | / | 180 | 1 |
| 2022.04.20 | Guangdong | Duck | / | 180 | 1 |
| 2022.04.20 | Guangdong | Duck | / | 180 | 1 |
| 2022.04.20 | Anhui | Duck | / | / | 1 |
| 2022.04.20 | Anhui | Duck | / | / | 1 |
| 2022.04.20 | Anhui | Duck | / | / | 1 |
| 2022.04.20 | Anhui | Duck | / | / | 1 |
| 2022.04.21 | Jiangxi | Duck | Mule Duck | 12 | 1 |
| 2022.04.21 | Jiangxi | Duck | White Duck | 45 | 1 |
| 2022.04.21 | Guangdong | Duck | White Duck | 17 | 1 |
| 2022.04.21 | Guangdong | Duck | / | 200 | 1 |
| 2022.04.21 | Guangdong | Duck | Shelduck | 45 | 1 |
| 2022.04.21 | Guangdong | Duck | Shelduck | 45 | 1 |
| 2022.04.21 | Shandong | Duck | / | 10 | 1 |
| 2022.04.21 | Shandong | Duck | / | 32 | 1 |
| 2022.04.21 | Shandong | Duck | / | 35 | 1 |
| 2022.04.21 | Shandong | Duck | / | 35 | 1 |
| 2022.04.21 | Shandong | Duck | / | 35 | 1 |
| 2022.04.21 | Shandong | Duck | / | 35 | 1 |
| 2022.04.22 | Shandong | Duck | / | 251 | 1 |
| 2022.04.24 | Sichuan | Duck | / | 16 | 1 |
| 2022.04.24 | Sichuan | Duck | / | 16 | 1 |
| 2022.04.24 | Sichuan | Duck | Slightly Spotted Duck | 80 | 1 |
| 2022.04.24 | Jiangxi | Duck | Mule Duck | 27 | 1 |
| 2022.04.24 | Jiangxi | Duck | Mule Duck | 27 | 1 |
| 2022.04.24 | Shandong | Duck | / | / | 1 |
| 2022.04.24 | Guangxi | Duck | Cherry Valley Duck | 29 | 1 |
| 2022.04.24 | Guangxi | Duck | Cherry Valley Duck | 29 | 1 |
| 2022.04.24 | Guangxi | Duck | Cherry Valley Duck | 29 | 1 |
| 2022.04.25 | Guangdong | Duck | / | 25 | 1 |
| 2022.04.25 | Guangdong | Duck | Muscovy Duck | 25 | 1 |
| 2022.04.25 | Guangxi | Duck | Cherry Valley Duck | 30 | 1 |
| 2022.04.25 | Guangxi | Duck | Cherry Valley Duck | 30 | 1 |
| 2022.04.25 | Guangxi | Duck | Cherry Valley Duck | 30 | 1 |
| 2022.04.25 | Guangxi | Duck | Cherry Valley Duck | 30 | 1 |
| 2022.04.25 | Guangxi | Duck | Cherry Valley Duck | 30 | 1 |
| 2022.04.25 | Guangxi | Duck | Cherry Valley Duck | 30 | 1 |
| 2022.04.25 | Guangxi | Duck | Cherry Valley Duck | 30 | 1 |
| 2022.04.25 | Guangxi | Duck | Cherry Valley Duck | 30 | 1 |
| 2022.04.25 | Guangxi | Duck | Cherry Valley Duck | 30 | 1 |
| 2022.04.25 | Guangxi | Duck | Cherry Valley Duck | 30 | 1 |
| 2022.04.25 | Guangxi | Duck | Cherry Valley Duck | 30 | 1 |
| 2022.04.25 | Guangxi | Duck | Cherry Valley Duck | 30 | 1 |
| 2022.04.25 | Guangxi | Duck | Cherry Valley Duck | 30 | 1 |
| 2022.04.25 | Guangxi | Duck | Cherry Valley Duck | 30 | 1 |
| 2022.04.25 | Guangdong | Duck | White Duck | 28 | 1 |
| 2022.04.25 | Guangdong | Duck | White Duck | 28 | 1 |
| 2022.04.25 | Guangdong | Duck | White Duck | 28 | 1 |
| 2022.04.25 | Guangdong | Duck | White Duck | 28 | 1 |
| 2022.04.25 | Guangdong | Duck | White Duck | 28 | 1 |
| 2022.04.25 | Guangdong | Duck | White Duck | 28 | 1 |
| 2022.04.25 | Guangdong | Duck | White Duck | 28 | 1 |
| 2022.04.25 | Guangdong | Duck | White Duck | 28 | 1 |
| 2022.04.25 | Guangdong | Duck | White Duck | 28 | 1 |
| 2022.04.25 | Guangdong | Duck | White Duck | 28 | 1 |
| 2022.04.25 | Guangdong | Duck | White Duck | 28 | 1 |
| 2022.04.25 | Guangdong | Duck | White Duck | 28 | 1 |
| 2022.04.25 | Guangdong | Duck | White Duck | 28 | 1 |
| 2022.04.25 | Guangdong | Duck | White Duck | 28 | 1 |
| 2022.04.25 | Guangdong | Duck | White Duck | 28 | 1 |
| 2022.04.25 | Guangdong | Duck | White Duck | 28 | 1 |
| 2022.04.25 | Guangdong | Duck | White Duck | 28 | 1 |
| 2022.04.25 | Guangdong | Duck | White Duck | 28 | 1 |
| 2022.04.25 | Guangdong | Duck | / | 20 | 1 |
| 2022.04.25 | Guangdong | Duck | / | 20 | 1 |
| 2022.04.25 | Guangdong | Duck | / | 20 | 1 |
| 2022.04.25 | Shandong | Duck | / | 32 | 1 |
| 2022.04.25 | Shandong | Duck | / | 32 | 1 |
| 2022.04.25 | Shannxi | Duck | Four-line Duck | 27 | 1 |
| 2022.04.25 | Shannxi | Duck | Four-line Duck | 27 | 1 |
| 2022.04.25 | Shannxi | Duck | Four-line Duck | 27 | 1 |
| 2022.04.25 | Shandong | Duck | / | 18 | 1 |
| 2022.04.26 | Fujian | Duck | Mule Duck | 20 | 1 |
| 2022.04.26 | Fujian | Duck | Mule Duck | 20 | 1 |
| 2022.04.26 | Fujian | Duck | Mule Duck | 20 | 1 |
| 2022.04.26 | Fujian | Duck | Mule Duck | 20 | 1 |
| 2022.04.26 | Fujian | Duck | Mule Duck | 20 | 1 |
| 2022.04.26 | Fujian | Duck | Mule Duck | 20 | 1 |
| 2022.04.26 | Fujian | Duck | Mule Duck | 20 | 1 |
| 2022.04.26 | Fujian | Duck | Mule Duck | 20 | 1 |
| 2022.04.26 | Fujian | Duck | Mule Duck | 20 | 1 |
| 2022.04.26 | Fujian | Duck | Mule Duck | 20 | 1 |
| 2022.04.26 | Fujian | Duck | Mule Duck | 20 | 1 |
| 2022.04.26 | Fujian | Duck | Mule Duck | 20 | 1 |
| 2022.04.26 | Fujian | Duck | Mule Duck | 46 | 1 |
| 2022.04.26 | Shandong | Duck | / | / | 1 |
| 2022.04.26 | Shandong | Duck | / | 15 | 1 |
| 2022.04.26 | Shandong | Duck | / | 15 | 1 |
| 2022.04.27 | Shandong | Duck | Cherry Valley Duck | 38 | 1 |
| 2022.04.28 | Shandong | Duck | White Duck | 30 | 1 |
| 2022.04.29 | Guangdong | Duck | White Duck | 33 | 1 |
| 2022.04.29 | Guangdong | Duck | White Duck | 16 | 1 |
| 2022.04.29 | Anhui | Duck | Muscovy Duck | / | 1 |
| 2022.04.29 | Anhui | Duck | Muscovy Duck | / | 1 |
| 2022.04.29 | Anhui | Duck | Muscovy Duck | / | 1 |
| 2022.04.29 | Anhui | Duck | Muscovy Duck | / | 1 |
| 2022.04.29 | Anhui | Duck | Muscovy Duck | / | 1 |
| 2022.04.29 | Anhui | Duck | Muscovy Duck | / | 1 |
| 2022.04.29 | Anhui | Duck | Muscovy Duck | / | 1 |
| 2022.04.29 | Anhui | Duck | Muscovy Duck | / | 1 |
| 2022.04.29 | Anhui | Duck | Muscovy Duck | / | 1 |
| 2022.04.29 | Anhui | Duck | Muscovy Duck | / | 1 |
| 2022.04.29 | Anhui | Duck | Muscovy Duck | / | 1 |
| 2022.04.29 | Anhui | Duck | Muscovy Duck | / | 1 |
| 2022.04.29 | Anhui | Duck | Muscovy Duck | / | 1 |
| 2022.04.29 | Anhui | Duck | Muscovy Duck | / | 1 |
| 2022.04.29 | Anhui | Duck | Muscovy Duck | / | 1 |
| 2022.04.29 | Anhui | Duck | Muscovy Duck | / | 1 |
| 2022.04.29 | Anhui | Duck | Muscovy Duck | / | 1 |
| 2022.04.29 | Anhui | Duck | Muscovy Duck | / | 1 |
| 2022.04.29 | Anhui | Duck | Muscovy Duck | / | 1 |
| 2022.04.29 | Anhui | Duck | Muscovy Duck | / | 1 |
| 2022.04.29 | Anhui | Duck | Muscovy Duck | / | 1 |
| 2022.04.29 | Anhui | Duck | Muscovy Duck | / | 1 |
| 2022.04.29 | Anhui | Duck | Muscovy Duck | / | 1 |
| 2022.04.29 | Anhui | Duck | Muscovy Duck | / | 1 |
| 2022.04.29 | Anhui | Duck | Muscovy Duck | / | 1 |
| 2022.04.29 | Anhui | Duck | Muscovy Duck | / | 1 |
| 2022.04.29 | Anhui | Duck | Muscovy Duck | / | 1 |
| 2022.04.29 | Anhui | Duck | Muscovy Duck | / | 1 |
| 2022.04.29 | Anhui | Duck | Muscovy Duck | / | 1 |
| 2022.04.29 | Anhui | Duck | Muscovy Duck | / | 1 |
| 2022.04.29 | Anhui | Duck | Muscovy Duck | / | 1 |
| 2022.04.29 | Hebei | Duck | Cherry Valley Duck | 26 | 1 |
| 2022.04.29 | Hebei | Duck | Cherry Valley Duck | 26 | 1 |
| 2022.04.29 | Hebei | Duck | Cherry Valley Duck | 26 | 1 |
| 2022.04.29 | Hebei | Duck | Cherry Valley Duck | 26 | 1 |
| 2022.04.29 | Hebei | Duck | Cherry Valley Duck | 26 | 1 |
| 2022.05.01 | Liaoning | Duck | / | 30 | 1 |
| 2022.05.01 | Hebei | Duck | / | 33 | 1 |
| 2022.05.01 | Guangdong | Duck | Mule Duck | 30 | 1 |
| 2022.05.05 | Shandong | Duck | / | 32 | 1 |
| 2022.05.05 | Shandong | Duck | / | 32 | 1 |
| 2022.05.05 | Shandong | Duck | / | 32 | 1 |
| 2022.05.05 | Shandong | Duck | / | 32 | 1 |
| 2022.05.05 | Shandong | Duck | / | 32 | 1 |
| 2022.05.05 | Shandong | Duck | / | 25 | 1 |
| 2022.05.05 | Shandong | Duck | / | 25 | 1 |
| 2022.05.05 | Shandong | Duck | / | 25 | 1 |
| 2022.05.05 | Hebei | Duck | Cherry Valley Duck | 35 | 1 |
| 2022.05.05 | Shannxi | Duck | Four-line Duck | 530 | 1 |
| 2022.05.05 | Shannxi | Duck | Four-line Duck | 530 | 1 |
| 2022.05.05 | Shannxi | Duck | Four-line Duck | 530 | 1 |
| 2022.05.05 | Shannxi | Duck | Four-line Duck | 530 | 1 |
| 2022.05.05 | Shandong | Duck | Cherry Valley Duck | 29 | 1 |
| 2022.05.06 | Guangdong | Duck | White Duck | 40 | 1 |
| 2022.05.06 | Guangdong | Duck | White Duck | 40 | 1 |
| 2022.05.06 | Guangdong | Duck | White Duck | 15 | 1 |
| 2022.05.06 | Guangdong | Duck | / | 100 | 1 |
| 2022.05.06 | Guangdong | Duck | / | 200 | 1 |
| 2022.05.06 | Guangdong | Duck | / | 200 | 1 |
| 2022.05.06 | Guangdong | Duck | / | 40 | 1 |
| 2022.05.07 | Shandong | Duck | / | 36 | 1 |
| 2022.05.07 | Anhui | Duck | / | 40 | 1 |
| 2022.05.09 | Shandong | Duck | / | 15 | 1 |
| 2022.05.09 | Shandong | Duck | / | 32 | 1 |
| 2022.05.09 | Shandong | Duck | / | 33 | 1 |
| 2022.05.09 | Shandong | Duck | / | 9 | 1 |
| 2022.05.09 | Shandong | Duck | / | 22 | 1 |
| 2022.05.09 | Shandong | Duck | / | 26 | 1 |
| 2022.05.09 | Hebei | Duck | Cherry Valley Duck | 1 | 1 |
| 2022.05.09 | Heilongjiang | Duck | / | 31 | 1 |
| 2022.05.09 | Heilongjiang | Duck | / | 31 | 1 |
| 2022.05.09 | Heilongjiang | Duck | / | 31 | 1 |
| 2022.05.09 | Shandong | Duck | Cherry Valley Duck | 29 | 1 |
| 2022.05.09 | Guangdong | Duck | / | 160 | 1 |
| 2022.05.10 | Shandong | Duck | / | 37 | 1 |
| 2022.05.10 | Guangdong | Duck | White Duck | 33 | 1 |
| 2022.05.10 | Guangdong | Duck | White Duck | 33 | 1 |
| 2022.05.10 | Guangdong | Duck | White Duck | 33 | 1 |
| 2022.05.10 | Guangdong | Duck | Muscovy Duck | 330 | 1 |
| 2022.05.10 | Guangdong | Duck | Muscovy Duck | 330 | 1 |
| 2022.05.10 | Guangdong | Duck | Muscovy Duck | 15 | 1 |
| 2022.05.10 | Guangdong | Duck | / | 12 | 1 |
| 2022.05.11 | Shandong | Duck | / | 31 | 1 |
| 2022.05.11 | Shandong | Duck | / | 27 | 1 |
| 2022.05.11 | Shandong | Duck | / | 14 | 1 |
| 2022.05.11 | Anhui | Duck | / | 279 | 1 |
| 2022.05.12 | Shandong | Duck | Peking Duck | 33 | 1 |
| 2022.05.12 | Shandong | Duck | / | 27 | 1 |
| 2022.05.12 | Guangxi | Duck | Cherry Valley Duck | 24 | 1 |
| 2022.05.12 | Guangxi | Duck | Cherry Valley Duck | 24 | 1 |
| 2022.05.12 | Guangxi | Duck | Cherry Valley Duck | 24 | 1 |
| 2022.05.12 | Guangxi | Duck | Cherry Valley Duck | 39 | 1 |
| 2022.05.12 | Guangxi | Duck | Cherry Valley Duck | 39 | 1 |
| 2022.05.12 | Guangxi | Duck | Cherry Valley Duck | 39 | 1 |
| 2022.05.12 | Guangxi | Duck | Cherry Valley Duck | 39 | 1 |
| 2022.05.12 | Guangxi | Duck | Cherry Valley Duck | 39 | 1 |
| 2022.05.12 | Guangxi | Duck | Cherry Valley Duck | 39 | 1 |
| 2022.05.12 | Guangxi | Duck | Cherry Valley Duck | 39 | 1 |
| 2022.05.12 | Guangxi | Duck | Cherry Valley Duck | 39 | 1 |
| 2022.05.12 | Guangxi | Duck | Cherry Valley Duck | 39 | 1 |
| 2022.05.12 | Guangxi | Duck | Cherry Valley Duck | 39 | 1 |
| 2022.05.12 | Guangxi | Duck | Cherry Valley Duck | 39 | 1 |
| 2022.05.12 | Guangxi | Duck | Cherry Valley Duck | 39 | 1 |
| 2022.05.13 | Shandong | Duck | / | 29 | 1 |
| 2022.05.13 | Hebei | Duck | Cherry Valley Duck | 38 | 1 |
| 2022.05.13 | Hebei | Duck | Cherry Valley Duck | 38 | 1 |
| 2022.05.13 | Hebei | Duck | Cherry Valley Duck | 38 | 1 |
| 2022.05.13 | Hebei | Duck | Cherry Valley Duck | 38 | 1 |
| 2022.05.13 | Shandong | Duck | / | 19 | 1 |
| 2022.05.13 | Shandong | Duck | / | 19 | 1 |
| 2022.05.13 | Shandong | Duck | / | 32 | 1 |
| 2022.05.13 | Shandong | Duck | / | / | 1 |
| 2022.05.13 | Shandong | Duck | / | / | 1 |
| 2022.05.13 | Shandong | Duck | / | / | 1 |
| 2022.05.13 | Shanxi | Duck | Cherry Valley Duck | 1 | 1 |
| 2022.05.16 | Shandong | Duck | / | 13 | 1 |
| 2022.05.16 | Shandong | Duck | / | 13 | 1 |
| 2022.05.16 | Shandong | Duck | / | 45 | 1 |
| 2022.05.16 | Shandong | Duck | Cherry Valley Duck | 32 | 1 |
| 2022.05.16 | Shandong | Duck | / | 18 | 1 |
| 2022.05.16 | Shandong | Duck | / | 33 | 1 |
| 2022.05.16 | Shandong | Duck | / | 1 | 1 |
| 2022.05.16 | Shandong | Duck | / | 1 | 1 |
| 2022.05.16 | Shandong | Duck | / | 1 | 1 |
| 2022.05.16 | Shandong | Duck | / | 1 | 1 |
| 2022.05.16 | Shandong | Duck | / | 1 | 1 |
| 2022.05.16 | Shandong | Duck | / | 1 | 1 |
| 2022.05.16 | Shandong | Duck | / | 1 | 1 |
| 2022.05.16 | Shandong | Duck | / | 1 | 1 |
| 2022.05.16 | Shandong | Duck | / | 1 | 1 |
| 2022.05.16 | Shandong | Duck | / | 30 | 1 |
| 2022.05.16 | Shannxi | Duck | Four-line Duck | 21 | 1 |
| 2022.05.16 | Shannxi | Duck | Four-line Duck | 21 | 1 |
| 2022.05.16 | Shannxi | Duck | Four-line Duck | 21 | 1 |
| 2022.05.16 | Shannxi | Duck | Four-line Duck | 21 | 1 |
| 2022.05.16 | Shannxi | Duck | Four-line Duck | 21 | 1 |
| 2022.05.16 | Shannxi | Duck | Four-line Duck | 21 | 1 |
| 2022.05.16 | Shannxi | Duck | Four-line Duck | 21 | 1 |
| 2022.05.16 | Shannxi | Duck | Four-line Duck | 21 | 1 |
| 2022.05.16 | Shandong | Duck | Cherry Valley Duck | 33 | 1 |
| 2022.05.16 | Fujian | Duck | Mule Duck | 70 | 1 |
| 2022.05.16 | Fujian | Duck | Mule Duck | 70 | 1 |
| 2022.05.16 | Fujian | Duck | Mule Duck | 70 | 1 |
| 2022.05.16 | Fujian | Duck | Mule Duck | 70 | 1 |
| 2022.05.16 | Fujian | Duck | Mule Duck | 73 | 1 |
| 2022.05.16 | Fujian | Duck | Mule Duck | 73 | 1 |
| 2022.05.16 | Fujian | Duck | Mule Duck | 73 | 1 |
| 2022.05.16 | Fujian | Duck | Mule Duck | 63 | 1 |
| 2022.05.16 | Fujian | Duck | Mule Duck | 63 | 1 |
| 2022.05.16 | Guangxi | Duck | Cherry Valley Duck | 42 | 1 |
| 2022.05.16 | Guangxi | Duck | Cherry Valley Duck | 42 | 1 |
| 2022.05.16 | Guangdong | Duck | / | 40 | 1 |
| 2022.05.17 | Anhui | Duck | / | 1 | 1 |
| 2022.05.17 | Anhui | Duck | / | 1 | 1 |
| 2022.05.17 | Anhui | Duck | / | 1 | 1 |
| 2022.05.17 | Anhui | Duck | / | 1 | 1 |
| 2022.05.17 | Anhui | Duck | / | 1 | 1 |
| 2022.05.17 | Anhui | Duck | / | 1 | 1 |
| 2022.05.17 | Anhui | Duck | / | 1 | 1 |
| 2022.05.17 | Anhui | Duck | / | 1 | 1 |
| 2022.05.17 | Anhui | Duck | / | 1 | 1 |
| 2022.05.17 | Anhui | Duck | / | 1 | 1 |
| 2022.05.17 | Anhui | Duck | / | 1 | 1 |
| 2022.05.17 | Anhui | Duck | / | 1 | 1 |
| 2022.05.17 | Anhui | Duck | / | 1 | 1 |
| 2022.05.17 | Anhui | Duck | / | 1 | 1 |
| 2022.05.17 | Anhui | Duck | / | 1 | 1 |
| 2022.05.17 | Anhui | Duck | / | 1 | 1 |
| 2022.05.17 | Anhui | Duck | / | 1 | 1 |
| 2022.05.17 | Anhui | Duck | / | 1 | 1 |
| 2022.05.17 | Anhui | Duck | / | 1 | 1 |
| 2022.05.17 | Anhui | Duck | / | 1 | 1 |
| 2022.05.18 | Shandong | Duck | / | 15 | 1 |
| 2022.05.18 | Hebei | Duck | Cherry Valley Duck | 33 | 1 |
| 2022.05.19 | Shandong | Duck | / | 28 | 1 |
| 2022.05.19 | Shandong | Duck | / | 28 | 1 |
| 2022.05.19 | Shandong | Duck | / | 28 | 1 |
| 2022.05.19 | Shandong | Duck | / | 24 | 1 |
| 2022.05.20 | Shandong | Duck | / | 32 | 1 |
| 2022.05.20 | Shandong | Duck | / | 32 | 1 |
| 2022.05.20 | Shandong | Duck | / | 1 | 1 |
| 2022.05.20 | Shandong | Duck | / | 1 | 1 |
| 2022.05.20 | Shandong | Duck | / | 30 | 1 |
| 2022.05.20 | Shandong | Duck | / | 30 | 1 |
| 2022.05.20 | Shandong | Duck | Zhongxin Duck | 42 | 1 |
| 2022.05.21 | Shandong | Duck | / | 36 | 1 |
| 2022.05.21 | Shandong | Duck | / | 36 | 1 |
| 2022.05.21 | Hebei | Duck | Cherry Valley Duck | 34 | 1 |
| 2022.05.23 | Hebei | Duck | Cherry Valley Duck | 39 | 1 |
| 2022.05.23 | Shandong | Duck | / | 32 | 1 |
| 2022.05.23 | Shandong | Duck | / | 32 | 1 |
| 2022.05.23 | Shandong | Duck | / | 32 | 1 |
| 2022.05.23 | Shandong | Duck | / | 32 | 1 |
| 2022.05.23 | Shandong | Duck | / | 32 | 1 |
| 2022.05.23 | Shandong | Duck | / | 33 | 1 |
| 2022.05.23 | Shandong | Duck | / | 12 | 1 |
| 2022.05.23 | Guangdong | Duck | White Duck | 22 | 1 |
| 2022.05.24 | Henan | Duck | White Duck | 14 | 1 |
| 2022.05.24 | Nei Mongol | Duck | / | 33 | 1 |
| 2022.05.24 | Anhui | Duck | / | 28 | 1 |
| 2022.05.24 | Anhui | Duck | / | 28 | 1 |
| 2022.05.25 | Shandong | Duck | / | 35 | 1 |
| 2022.05.26 | Shandong | Duck | / | 35 | 1 |
| 2022.05.26 | Shandong | Duck | / | 35 | 1 |
| 2022.05.26 | Shandong | Duck | / | 35 | 1 |
| 2022.05.26 | Shandong | Duck | / | 0 | 1 |
| 2022.05.26 | Shandong | Duck | / | 0 | 1 |
| 2022.05.26 | Shandong | Duck | / | 0 | 1 |
| 2022.05.26 | Anhui | Duck | / | 36 | 1 |
| 2022.05.26 | Henan | Duck | / | 35 | 1 |
| 2022.05.26 | Henan | Duck | / | 35 | 1 |
| 2022.05.26 | Henan | Duck | / | 35 | 1 |
| 2022.05.26 | Henan | Duck | / | 35 | 1 |
| 2022.05.26 | Henan | Duck | / | 35 | 1 |
| 2022.05.26 | Henan | Duck | / | 35 | 1 |
| 2022.05.27 | Shandong | Duck | / | 33 | 1 |
| 2022.05.27 | Shandong | Duck | / | 23 | 1 |
| 2022.05.27 | Shandong | Duck | / | 32 | 1 |
| 2022.05.27 | Jiangxi | Duck | Shelduck | 40 | 1 |
| 2022.05.27 | Jiangxi | Duck | Shelduck | 40 | 1 |
| 2022.05.27 | Guizhou | Duck | / | 300 | 1 |
| 2022.05.27 | Guizhou | Duck | / | 300 | 1 |
| 2022.05.30 | Nei Mongol | Duck | / | 24 | 1 |
| 2022.05.30 | Nei Mongol | Duck | / | 24 | 1 |
| 2022.05.30 | Nei Mongol | Duck | / | 24 | 1 |
| 2022.05.30 | Nei Mongol | Duck | / | 36 | 1 |
| 2022.05.30 | Shandong | Duck | / | 30 | 1 |
| 2022.05.30 | Shandong | Duck | Cherry Valley Duck | 33 | 1 |
| 2022.05.30 | Shandong | Duck | Cherry Valley Duck | 30 | 1 |
| 2022.05.30 | Shandong | Duck | / | 27 | 1 |
| 2022.05.30 | Guangdong | Duck | White Duck | 13 | 1 |
| 2022.05.30 | Guangdong | Duck | White Duck | 13 | 1 |
| 2022.05.30 | Guangdong | Duck | White Duck | 13 | 1 |
| 2022.05.30 | Guangdong | Duck | White Duck | 13 | 1 |
| 2022.05.30 | Guangdong | Duck | White Duck | 13 | 1 |
| 2022.05.30 | Guangdong | Duck | White Duck | 13 | 1 |
| 2022.05.30 | Fujian | Duck | Cherry Valley Duck | 2 | 1 |
| 2022.05.30 | Fujian | Duck | Cherry Valley Duck | 2 | 1 |
| 2022.05.30 | Fujian | Duck | Cherry Valley Duck | 2 | 1 |
| 2022.05.30 | Fujian | Duck | Cherry Valley Duck | 2 | 1 |
| 2022.05.30 | Fujian | Duck | Cherry Valley Duck | 2 | 1 |
| 2022.05.30 | Fujian | Duck | Cherry Valley Duck | 2 | 1 |
| 2022.05.30 | Fujian | Duck | Cherry Valley Duck | 2 | 1 |
| 2022.05.30 | Fujian | Duck | Cherry Valley Duck | 2 | 1 |
| 2022.05.30 | Fujian | Duck | Cherry Valley Duck | 2 | 1 |
| 2022.05.30 | Fujian | Duck | Cherry Valley Duck | 2 | 1 |
| 2022.05.30 | Fujian | Duck | Cherry Valley Duck | 2 | 1 |
| 2022.05.30 | Fujian | Duck | Cherry Valley Duck | 2 | 1 |
| 2022.05.30 | Fujian | Duck | Cherry Valley Duck | 2 | 1 |
| 2022.05.30 | Fujian | Duck | Cherry Valley Duck | 2 | 1 |
| 2022.05.30 | Fujian | Duck | Cherry Valley Duck | 2 | 1 |
| 2022.05.30 | Fujian | Duck | Cherry Valley Duck | 2 | 1 |
| 2022.05.31 | Hebei | Duck | / | 39 | 1 |
| 2022.05.31 | Hebei | Duck | / | 39 | 1 |
| 2022.06.01 | Guangdong | Duck | / | / | 1 |
| 2022.06.01 | Guangdong | Duck | / | / | 1 |
| 2022.06.02 | Anhui | Duck | / | 20 | 1 |
| 2022.06.02 | Guangdong | Duck | White Duck | 20 | 1 |
| 2022.06.02 | Guangdong | Duck | Muscovy Duck | 15 | 1 |
| 2022.06.02 | Guangdong | Duck | / | 150 | 1 |
| 2022.06.02 | Guangdong | Duck | White Duck | 30 | 1 |
| 2022.06.06 | Hebei | Duck | Cherry Valley Duck | 23 | 1 |
| 2022.06.06 | Hebei | Duck | Cherry Valley Duck | 23 | 1 |
| 2022.06.06 | Hebei | Duck | Cherry Valley Duck | 23 | 1 |
| 2022.06.06 | Hebei | Duck | Cherry Valley Duck | 23 | 1 |
| 2022.06.06 | Hebei | Duck | Cherry Valley Duck | 23 | 1 |
| 2022.06.06 | Hebei | Duck | Cherry Valley Duck | 23 | 1 |
| 2022.06.06 | Shandong | Duck | / | 33 | 1 |
| 2022.06.06 | Guangdong | Duck | / | 55 | 1 |
| 2022.06.06 | Guangdong | Duck | / | 55 | 1 |
| 2022.06.06 | Guangdong | Duck | / | 55 | 1 |
| 2022.06.06 | Guangdong | Duck | / | 55 | 1 |
| 2022.06.06 | Guangdong | Duck | / | 55 | 1 |
| 2022.06.06 | Guangdong | Duck | / | 55 | 1 |
| 2022.06.06 | Guangdong | Duck | / | 55 | 1 |
| 2022.06.06 | Guangdong | Duck | / | 55 | 1 |
| 2022.06.06 | Guangdong | Duck | / | 55 | 1 |
| 2022.06.06 | Guangdong | Duck | / | 60 | 1 |
| 2022.06.06 | Guangdong | Duck | / | 60 | 1 |
| 2022.06.06 | Guangdong | Duck | / | 60 | 1 |
| 2022.06.06 | Guangdong | Duck | / | 60 | 1 |
| 2022.06.06 | Guangdong | Duck | White Duck | 20 | 1 |
| 2022.06.06 | Guangdong | Duck | Muscovy Duck | 20 | 1 |
| 2022.06.07 | Sichuan | Duck | / | 120 | 1 |
| 2022.06.07 | Sichuan | Duck | / | 120 | 1 |
| 2022.06.07 | Sichuan | Duck | / | 120 | 1 |
| 2022.06.07 | Sichuan | Duck | / | 120 | 1 |
| 2022.06.07 | Sichuan | Duck | / | 120 | 1 |
| 2022.06.07 | Sichuan | Duck | / | 120 | 1 |
| 2022.06.07 | Sichuan | Duck | / | 120 | 1 |
| 2022.06.07 | Sichuan | Duck | / | 120 | 1 |
| 2022.06.07 | Sichuan | Duck | / | 120 | 1 |
| 2022.06.07 | Jiangsu | Duck | / | 323 | 1 |
| 2022.06.07 | Jiangsu | Duck | / | 323 | 1 |
| 2022.06.07 | Jiangsu | Duck | / | 323 | 1 |
| 2022.06.07 | Jiangsu | Duck | / | 323 | 1 |
| 2022.06.07 | Jiangsu | Duck | / | 323 | 1 |
| 2022.06.07 | Jiangxi | Duck | Muscovy Duck | 30 | 1 |
| 2022.06.07 | Jiangxi | Duck | Muscovy Duck | 30 | 1 |
| 2022.06.08 | Shandong | Duck | / | 20 | 1 |
| 2022.06.08 | Shandong | Duck | / | 20 | 1 |
| 2022.06.08 | Shandong | Duck | / | 30 | 1 |
| 2022.06.08 | Shandong | Duck | / | 30 | 1 |
| 2022.06.09 | Shandong | Duck | / | 30 | 1 |
| 2022.06.09 | Shandong | Duck | / | 21 | 1 |
| 2022.06.09 | Shandong | Duck | / | 21 | 1 |
| 2022.06.09 | Shandong | Duck | / | 21 | 1 |
| 2022.06.09 | Shandong | Duck | / | 21 | 1 |
| 2022.06.10 | Guangdong | Duck | Shelduck | 60 | 1 |
| 2022.06.10 | Guangdong | Duck | Shelduck | 60 | 1 |
| 2022.06.13 | Shandong | Duck | / | 26 | 1 |
| 2022.06.13 | Shandong | Duck | / | 26 | 1 |
| 2022.06.13 | Henan | Duck | Cherry Valley Duck | 17 | 1 |
| 2022.06.13 | Shandong | Duck | / | 30 | 1 |
| 2022.06.13 | Shandong | Duck | / | 23 | 1 |
| 2022.06.13 | Shandong | Duck | / | 27 | 1 |
| 2022.06.13 | Shandong | Duck | / | 20 | 1 |
| 2022.06.13 | Shandong | Duck | / | 30 | 1 |
| 2022.06.13 | Shandong | Duck | / | 21 | 1 |
| 2022.06.13 | Shandong | Duck | / | 30 | 1 |
| 2022.06.13 | Shandong | Duck | White Duck | 13 | 1 |
| 2022.06.13 | Anhui | Duck | / | 29 | 1 |
| 2022.06.13 | Anhui | Duck | / | 29 | 1 |
| 2022.06.13 | Guangdong | Duck | White Duck | 30 | 1 |
| 2022.06.13 | Guangdong | Duck | White Duck | 30 | 1 |
| 2022.06.13 | Guangdong | Duck | White Duck | 30 | 1 |
| 2022.06.13 | Guangdong | Duck | White Duck | 30 | 1 |
| 2022.06.13 | Guangdong | Duck | Shelduck | / | 1 |
| 2022.06.13 | Guangdong | Duck | Shelduck | / | 1 |
| 2022.06.13 | Guangdong | Duck | Shelduck | / | 1 |
| 2022.06.13 | Guangdong | Duck | Shelduck | / | 1 |
| 2022.06.13 | Guangdong | Duck | Shelduck | / | 1 |
| 2022.06.13 | Guangdong | Duck | Shelduck | / | 1 |
| 2022.06.13 | Guangdong | Duck | Shelduck | / | 1 |
| 2022.06.13 | Guangdong | Duck | White Duck | 35 | 1 |
| 2022.06.13 | Guangdong | Duck | White Duck | 35 | 1 |
| 2022.06.14 | Shandong | Duck | / | 32 | 1 |
| 2022.06.14 | Shandong | Duck | / | 32 | 1 |
| 2022.06.14 | Shandong | Duck | / | 32 | 1 |
| 2022.06.14 | Anhui | Duck | Cherry Valley Duck | 37 | 1 |
| 2022.06.14 | Guangdong | Duck | Muscovy Duck | 30 | 1 |
| 2022.06.14 | Guangdong | Duck | Muscovy Duck | 30 | 1 |
| 2022.06.14 | Guangdong | Duck | Muscovy Duck | 30 | 1 |
| 2022.06.14 | Guangdong | Duck | Muscovy Duck | 30 | 1 |
| 2022.06.14 | Guangdong | Duck | Muscovy Duck | 30 | 1 |
| 2022.06.14 | Guangdong | Duck | Muscovy Duck | 30 | 1 |
| 2022.06.16 | Anhui | Duck | / | 1 | 1 |
| 2022.06.16 | Anhui | Duck | / | 1 | 1 |
| 2022.06.16 | Anhui | Duck | / | 1 | 1 |
| 2022.06.16 | Anhui | Duck | / | 1 | 1 |
| 2022.06.16 | Anhui | Duck | / | 1 | 1 |
| 2022.06.16 | Anhui | Duck | / | 1 | 1 |
| 2022.06.16 | Anhui | Duck | / | 1 | 1 |
| 2022.06.16 | Anhui | Duck | / | 1 | 1 |
| 2022.06.16 | Anhui | Duck | / | 1 | 1 |
| 2022.06.16 | Anhui | Duck | / | 1 | 1 |
| 2022.06.16 | Anhui | Duck | / | 1 | 1 |
| 2022.06.16 | Anhui | Duck | / | 1 | 1 |
| 2022.06.16 | Anhui | Duck | / | 1 | 1 |
| 2022.06.16 | Anhui | Duck | / | 1 | 1 |
| 2022.06.16 | Anhui | Duck | / | 1 | 1 |
| 2022.06.16 | Anhui | Duck | / | 1 | 1 |
| 2022.06.16 | Guangdong | Duck | / | 35 | 1 |
| 2022.06.16 | Guangdong | Duck | / | 35 | 1 |
| 2022.06.16 | Guangdong | Duck | / | 35 | 1 |
| 2022.06.16 | Guangdong | Duck | / | 35 | 1 |
| 2022.06.17 | Henan | Duck | White Duck | 1 | 1 |
| 2022.06.17 | Henan | Duck | White Duck | 1 | 1 |
| 2022.06.17 | Henan | Duck | White Duck | 1 | 1 |
| 2022.06.17 | Henan | Duck | White Duck | 1 | 1 |
| 2022.06.17 | Henan | Duck | White Duck | 1 | 1 |
| 2022.06.17 | Henan | Duck | White Duck | 1 | 1 |
| 2022.06.17 | Henan | Duck | White Duck | 1 | 1 |
| 2022.06.17 | Henan | Duck | White Duck | 1 | 1 |
| 2022.06.17 | Henan | Duck | White Duck | 1 | 1 |
| 2022.06.17 | Henan | Duck | White Duck | 1 | 1 |
| 2022.06.17 | Shandong | Duck | / | / | 1 |
| 2022.06.17 | Shandong | Duck | / | / | 1 |
| 2022.06.17 | Shandong | Duck | / | / | 1 |
| 2022.06.17 | Shandong | Duck | White Duck | 21 | 1 |
| 2022.06.17 | Anhui | Duck | / | / | 1 |
| 2022.06.17 | Anhui | Duck | / | / | 1 |
| 2022.06.17 | Anhui | Duck | / | / | 1 |
| 2022.06.17 | Anhui | Duck | / | / | 1 |
| 2022.06.17 | Anhui | Duck | / | / | 1 |
| 2022.06.17 | Anhui | Duck | / | / | 1 |
| 2022.06.17 | Anhui | Duck | / | / | 1 |
| 2022.06.17 | Anhui | Duck | / | / | 1 |
| 2022.06.17 | Anhui | Duck | / | / | 1 |
| 2022.06.17 | Anhui | Duck | / | / | 1 |
| 2022.06.17 | Anhui | Duck | / | / | 1 |
| 2022.06.17 | Anhui | Duck | / | / | 1 |
| 2022.06.17 | Anhui | Duck | / | / | 1 |
| 2022.06.17 | Anhui | Duck | / | / | 1 |
| 2022.06.17 | Anhui | Duck | / | / | 1 |
| 2022.06.17 | Anhui | Duck | / | / | 1 |
| 2022.06.17 | Guangdong | Duck | / | 34 | 1 |
| 2022.06.20 | Hebei | Duck | White Duck | 36 | 1 |
| 2022.06.20 | Hebei | Duck | White Duck | 36 | 1 |
| 2022.06.20 | Hebei | Duck | White Duck | 36 | 1 |
| 2022.06.20 | Hebei | Duck | White Duck | 36 | 1 |
| 2022.06.20 | Hebei | Duck | White Duck | 36 | 1 |
| 2022.06.20 | Hebei | Duck | White Duck | 36 | 1 |
| 2022.06.20 | Hebei | Duck | White Duck | 36 | 1 |
| 2022.06.20 | Hebei | Duck | White Duck | 36 | 1 |
| 2022.06.20 | Hebei | Duck | White Duck | 36 | 1 |
| 2022.06.20 | Hebei | Duck | White Duck | 36 | 1 |
| 2022.06.20 | Hebei | Duck | White Duck | 36 | 1 |
| 2022.06.20 | Hebei | Duck | White Duck | 36 | 1 |
| 2022.06.20 | Hebei | Duck | White Duck | 36 | 1 |
| 2022.06.20 | Hebei | Duck | White Duck | 36 | 1 |
| 2022.06.20 | Hebei | Duck | White Duck | 36 | 1 |
| 2022.06.20 | Hebei | Duck | White Duck | 36 | 1 |
| 2022.06.20 | Hebei | Duck | White Duck | 36 | 1 |
| 2022.06.20 | Hebei | Duck | White Duck | 36 | 1 |
| 2022.06.20 | Hebei | Duck | White Duck | 36 | 1 |
| 2022.06.20 | Hebei | Duck | White Duck | 36 | 1 |
| 2022.06.20 | Anhui | Duck | / | 35 | 1 |
| 2022.06.20 | Guangdong | Duck | White Duck | 40 | 1 |
| 2022.06.20 | Guangdong | Duck | White Duck | 15 | 1 |
| 2022.06.20 | Guangdong | Duck | White Duck | 35 | 1 |
| 2022.06.20 | Guangdong | Duck | White Duck | 40 | 1 |
| 2022.06.20 | Jiangxi | Duck | Muscovy Duck | 18 | 1 |
| 2022.06.20 | Jiangxi | Duck | Muscovy Duck | 18 | 1 |
| 2022.06.20 | Jiangxi | Duck | Muscovy Duck | 18 | 1 |
| 2022.06.21 | Shandong | Duck | / | 29 | 1 |
| 2022.06.21 | Jiangsu | Duck | / | / | 1 |
| 2022.06.21 | Jiangsu | Duck | / | / | 1 |
| 2022.06.21 | Jiangsu | Duck | / | / | 1 |
| 2022.06.21 | Jiangsu | Duck | / | / | 1 |
| 2022.06.21 | Shandong | Duck | / | 28 | 1 |
| 2022.06.21 | Shandong | Duck | / | 28 | 1 |
| 2022.06.21 | Shandong | Duck | / | 28 | 1 |
| 2022.06.21 | Shandong | Duck | / | 28 | 1 |
| 2022.06.21 | Hebei | Duck | White Duck | 22 | 1 |
| 2022.06.21 | Hebei | Duck | White Duck | 22 | 1 |
| 2022.06.21 | Hebei | Duck | Cherry Valley Duck | 2 | 1 |
| 2022.06.21 | Hebei | Duck | Cherry Valley Duck | 2 | 1 |
| 2022.06.21 | Hebei | Duck | Cherry Valley Duck | 2 | 1 |
| 2022.06.21 | Hebei | Duck | Cherry Valley Duck | 2 | 1 |
| 2022.06.22 | Shandong | Duck | White Duck | 3 | 1 |
| 2022.06.22 | Shandong | Duck | White Duck | 3 | 1 |
| 2022.06.22 | Shandong | Duck | White Duck | 3 | 1 |
| 2022.06.22 | Shandong | Duck | White Duck | 3 | 1 |
| 2022.06.22 | Shandong | Duck | White Duck | 3 | 1 |
| 2022.06.22 | Shandong | Duck | White Duck | 3 | 1 |
| 2022.06.22 | Shandong | Duck | White Duck | 3 | 1 |
| 2022.06.22 | Shandong | Duck | White Duck | 3 | 1 |
| 2022.06.22 | Shandong | Duck | White Duck | 3 | 1 |
| 2022.06.22 | Shandong | Duck | White Duck | 3 | 1 |
| 2022.06.22 | Shandong | Duck | White Duck | 3 | 1 |
| 2022.06.22 | Shandong | Duck | White Duck | 3 | 1 |
| 2022.06.22 | Shandong | Duck | White Duck | 3 | 1 |
| 2022.06.22 | Shandong | Duck | White Duck | 3 | 1 |
| 2022.06.22 | Shandong | Duck | White Duck | 3 | 1 |
| 2022.06.22 | Shandong | Duck | / | 18 | 1 |
| 2022.06.22 | Shandong | Duck | / | 18 | 1 |
| 2022.06.22 | Guangxi | Duck | Cherry Valley Duck | 14 | 1 |
| 2022.06.22 | Guangxi | Duck | Cherry Valley Duck | 14 | 1 |
| 2022.06.22 | Guangxi | Duck | Cherry Valley Duck | 14 | 1 |
| 2022.06.22 | Guangxi | Duck | Cherry Valley Duck | 14 | 1 |
| 2022.06.23 | Shandong | Duck | / | 27 | 1 |
| 2022.06.23 | Anhui | Duck | / | 1 | 1 |
| 2022.06.23 | Anhui | Duck | / | 1 | 1 |
| 2022.06.23 | Anhui | Duck | / | 1 | 1 |
| 2022.06.23 | Anhui | Duck | / | 1 | 1 |
| 2022.06.23 | Anhui | Duck | / | 1 | 1 |
| 2022.06.23 | Anhui | Duck | / | 1 | 1 |
| 2022.06.23 | Anhui | Duck | / | 1 | 1 |
| 2022.06.23 | Anhui | Duck | / | 1 | 1 |
| 2022.06.23 | Anhui | Duck | / | 1 | 1 |
| 2022.06.23 | Guangdong | Duck | / | 40 | 1 |
| 2022.06.23 | Guangdong | Duck | / | 22 | 1 |
| 2022.06.23 | Guangdong | Duck | / | 45 | 1 |
| 2022.06.23 | Guangdong | Duck | White Duck | 40 | 1 |
| 2022.06.23 | Guangdong | Duck | White Duck | 20 | 1 |
| 2022.06.23 | Guangdong | Duck | / | 20 | 1 |
| 2022.06.23 | Guangdong | Duck | / | 20 | 1 |
| 2022.06.23 | Guangdong | Duck | / | 20 | 1 |
| 2022.06.24 | Zhejiang | Duck | Muscovy Duck | 12 | 1 |
| 2022.06.24 | Zhejiang | Duck | Muscovy Duck | 12 | 1 |
| 2022.06.27 | Hebei | Duck | Cherry Valley Duck | 12 | 1 |
| 2022.06.27 | Jiangxi | Duck | Muscovy Duck | 18 | 1 |
| 2022.06.27 | Jiangxi | Duck | Muscovy Duck | 18 | 1 |
| 2022.06.28 | Shandong | Duck | / | 34 | 1 |
| 2022.06.28 | Shandong | Duck | / | 34 | 1 |
| 2022.06.28 | Shandong | Duck | / | 34 | 1 |
| 2022.06.28 | Shandong | Duck | / | 21 | 1 |
| 2022.06.28 | Anhui | Duck | / | 16 | 1 |
| 2022.06.28 | Anhui | Duck | / | 16 | 1 |
| 2022.06.28 | Guangdong | Duck | White Duck | 20 | 1 |
| 2022.06.28 | Guangdong | Duck | White Duck | 20 | 1 |
| 2022.06.28 | Guangdong | Duck | / | 28 | 1 |
| 2022.06.29 | Shandong | Duck | White Duck | 33 | 1 |
| 2022.06.29 | Jiangsu | Duck | / | 31 | 1 |
| 2022.06.29 | Jiangsu | Duck | / | 31 | 1 |
| 2022.06.30 | Shandong | Duck | / | 18 | 1 |
| 2022.06.30 | Shandong | Duck | / | 30 | 1 |
| 2022.06.30 | Guangxi | Duck | / | 14 | 1 |
| 2022.07.01 | Shandong | Duck | / | 20 | 1 |
| 2022.07.01 | Shandong | Duck | / | 20 | 1 |
| 2022.07.01 | Shandong | Duck | / | 20 | 1 |
| 2022.07.01 | Shandong | Duck | / | 20 | 1 |
| 2022.07.01 | Guangdong | Duck | White Duck | 24 | 1 |
| 2022.07.01 | Guangdong | Duck | White Duck | 24 | 1 |
| 2022.07.01 | Guangdong | Duck | White Duck | 24 | 1 |
| 2022.07.01 | Guangdong | Duck | White Duck | 24 | 1 |
| 2022.07.01 | Guangdong | Duck | White Duck | 24 | 1 |
| 2022.07.01 | Guangxi | Duck | / | 15 | 1 |
| 2022.07.02 | Shandong | Duck | / | 14 | 1 |
| 2022.07.04 | Shandong | Duck | / | 29 | 1 |
| 2022.07.04 | Heilongjiang | Duck | / | 18 | 1 |
| 2022.07.04 | Heilongjiang | Duck | / | 18 | 1 |
| 2022.07.04 | Heilongjiang | Duck | / | / | 1 |
| 2022.07.04 | Heilongjiang | Duck | / | / | 1 |
| 2022.07.04 | Guangdong | Duck | White Duck | 28 | 1 |
| 2022.07.04 | Jiangxi | Duck | Muscovy Duck | 18 | 1 |
| 2022.07.05 | Henan | Duck | / | 18 | 1 |
| 2022.07.05 | Guangdong | Duck | Shelduck | 16 | 1 |
| 2022.07.05 | Guangdong | Duck | Shelduck | 28 | 1 |
| 2022.07.05 | Guangdong | Duck | Shelduck | 15 | 1 |
| 2022.07.05 | Guangdong | Duck | Shelduck | 15 | 1 |
| 2022.07.05 | Guangdong | Duck | Shelduck | 15 | 1 |
| 2022.07.05 | Guangdong | Duck | Shelduck | 15 | 1 |
| 2022.07.06 | Shandong | Duck | / | 26 | 1 |
| 2022.07.07 | Anhui | Duck | / | 21 | 1 |
| 2022.07.08 | Guangdong | Duck | Mule Duck | 40 | 1 |
| 2022.07.09 | Anhui | Duck | / | 34 | 1 |
| 2022.07.09 | Guangdong | Duck | / | 30 | 1 |
| 2022.07.09 | Guangdong | Duck | / | 30 | 1 |
| 2022.07.09 | Guangdong | Duck | / | 30 | 1 |
| 2022.07.11 | Jiangxi | Duck | Shelduck | 40 | 1 |
| 2022.07.11 | Jiangxi | Duck | Shelduck | 40 | 1 |
| 2022.07.12 | Shandong | Duck | / | 35 | 1 |
| 2022.07.12 | Henan | Duck | / | 39 | 1 |
| 2022.07.12 | Henan | Duck | / | 39 | 1 |
| 2022.07.12 | Henan | Duck | / | 39 | 1 |
| 2022.07.12 | Henan | Duck | / | 39 | 1 |
| 2022.07.12 | Henan | Duck | / | 39 | 1 |
| 2022.07.13 | Shandong | Duck | / | 30 | 1 |
| 2022.07.14 | Anhui | Duck | / | 1 | 1 |
| 2022.07.14 | Anhui | Duck | / | 1 | 1 |
| 2022.07.14 | Anhui | Duck | / | 1 | 1 |
| 2022.07.14 | Anhui | Duck | / | 1 | 1 |
| 2022.07.14 | Anhui | Duck | / | 1 | 1 |
| 2022.07.14 | Anhui | Duck | / | 1 | 1 |
| 2022.07.14 | Anhui | Duck | / | 1 | 1 |
| 2022.07.14 | Anhui | Duck | / | 1 | 1 |
| 2022.07.14 | Anhui | Duck | / | 1 | 1 |
| 2022.07.14 | Anhui | Duck | / | 1 | 1 |
| 2022.07.14 | Anhui | Duck | / | 1 | 1 |
| 2022.07.14 | Anhui | Duck | / | 1 | 1 |
| 2022.07.14 | Shandong | Duck | Cherry Valley Duck | 4 | 1 |
| 2022.07.14 | Shandong | Duck | Cherry Valley Duck | 4 | 1 |
| 2022.07.14 | Shandong | Duck | Cherry Valley Duck | 4 | 1 |
| 2022.07.14 | Shandong | Duck | Cherry Valley Duck | 4 | 1 |
| 2022.07.14 | Shandong | Duck | Cherry Valley Duck | 4 | 1 |
| 2022.07.14 | Shandong | Duck | Cherry Valley Duck | 4 | 1 |
| 2022.07.14 | Shandong | Duck | Cherry Valley Duck | 4 | 1 |
| 2022.07.14 | Shandong | Duck | Cherry Valley Duck | 4 | 1 |
| 2022.07.14 | Shandong | Duck | Cherry Valley Duck | 4 | 1 |
| 2022.07.14 | Shandong | Duck | Cherry Valley Duck | 4 | 1 |
| 2022.07.14 | Shandong | Duck | Cherry Valley Duck | 4 | 1 |
| 2022.07.14 | Shandong | Duck | Cherry Valley Duck | 4 | 1 |
| 2022.07.14 | Shandong | Duck | Cherry Valley Duck | 4 | 1 |
| 2022.07.14 | Shandong | Duck | Cherry Valley Duck | 4 | 1 |
| 2022.07.14 | Shandong | Duck | Cherry Valley Duck | 4 | 1 |
| 2022.07.14 | Shandong | Duck | Cherry Valley Duck | 4 | 1 |
| 2022.07.14 | Shandong | Duck | Cherry Valley Duck | 4 | 1 |
| 2022.07.14 | Shandong | Duck | Cherry Valley Duck | 4 | 1 |
| 2022.07.14 | Shandong | Duck | Cherry Valley Duck | 4 | 1 |
| 2022.07.14 | Shandong | Duck | Cherry Valley Duck | 4 | 1 |
| 2022.07.14 | Shandong | Duck | Cherry Valley Duck | 4 | 1 |
| 2022.07.14 | Shandong | Duck | Cherry Valley Duck | 4 | 1 |
| 2022.07.14 | Shandong | Duck | Cherry Valley Duck | 4 | 1 |
| 2022.07.14 | Shandong | Duck | Cherry Valley Duck | 4 | 1 |
| 2022.07.14 | Shandong | Duck | / | 91 | 1 |
| 2022.07.14 | Shandong | Duck | / | 91 | 1 |
| 2022.07.14 | Shandong | Duck | / | 91 | 1 |
| 2022.07.14 | Jiangxi | Duck | Muscovy Duck | 18 | 1 |
| 2022.07.14 | Jiangxi | Duck | White Duck | 20 | 1 |
| 2022.07.15 | Hebei | Duck | Cherry Valley Duck | 42 | 1 |
| 2022.07.15 | Hebei | Duck | Cherry Valley Duck | 42 | 1 |
| 2022.07.15 | Shandong | Duck | / | 20 | 1 |
| 2022.07.15 | Shandong | Duck | / | 20 | 1 |
| 2022.07.15 | Shandong | Duck | / | 20 | 1 |
| 2022.07.15 | Anhui | Duck | / | 1 | 1 |
| 2022.07.15 | Anhui | Duck | / | 1 | 1 |
| 2022.07.15 | Anhui | Duck | / | 1 | 1 |
| 2022.07.15 | Anhui | Duck | / | 1 | 1 |
| 2022.07.15 | Anhui | Duck | / | 1 | 1 |
| 2022.07.15 | Anhui | Duck | / | 1 | 1 |
| 2022.07.15 | Anhui | Duck | / | 1 | 1 |
| 2022.07.15 | Anhui | Duck | / | 1 | 1 |
| 2022.07.15 | Shandong | Duck | White Duck | 1 | 1 |
| 2022.07.15 | Shandong | Duck | White Duck | 1 | 1 |
| 2022.07.15 | Shandong | Duck | White Duck | 1 | 1 |
| 2022.07.15 | Shandong | Duck | White Duck | 1 | 1 |
| 2022.07.15 | Shandong | Duck | White Duck | 1 | 1 |
| 2022.07.15 | Shandong | Duck | White Duck | 1 | 1 |
| 2022.07.15 | Shandong | Duck | White Duck | 1 | 1 |
| 2022.07.15 | Shandong | Duck | White Duck | 1 | 1 |
| 2022.07.15 | Shandong | Duck | White Duck | 1 | 1 |
| 2022.07.15 | Shandong | Duck | White Duck | 1 | 1 |
| 2022.07.15 | Shandong | Duck | White Duck | 1 | 1 |
| 2022.07.15 | Shandong | Duck | White Duck | 1 | 1 |
| 2022.07.15 | Shandong | Duck | White Duck | 1 | 1 |
| 2022.07.15 | Nei Mongol | Duck | / | 18 | 1 |
| 2022.07.15 | Nei Mongol | Duck | / | 18 | 1 |
| 2022.07.15 | Nei Mongol | Duck | / | 18 | 1 |
| 2022.07.15 | Guangxi | Duck | Cherry Valley Duck | 24 | 1 |
| 2022.07.15 | Guangxi | Duck | Cherry Valley Duck | 24 | 1 |
| 2022.07.15 | Guangxi | Duck | Cherry Valley Duck | 24 | 1 |
| 2022.07.15 | Guangdong | Duck | White Duck | 45 | 1 |
| 2022.07.15 | Guangdong | Duck | White Duck | 45 | 1 |
| 2022.07.15 | Guangdong | Duck | White Duck | 45 | 1 |
| 2022.07.15 | Guangdong | Duck | White Duck | 45 | 1 |
| 2022.07.15 | Guangdong | Duck | White Duck | 45 | 1 |
| 2022.07.15 | Guangdong | Duck | White Duck | 45 | 1 |
| 2022.07.15 | Guangdong | Duck | White Duck | 45 | 1 |
| 2022.07.15 | Guangdong | Duck | White Duck | 45 | 1 |
| 2022.07.15 | Guangdong | Duck | White Duck | 45 | 1 |
| 2022.07.15 | Guangdong | Duck | White Duck | 45 | 1 |
| 2022.07.15 | Guangdong | Duck | White Duck | 45 | 1 |
| 2022.07.15 | Guangdong | Duck | White Duck | 45 | 1 |
| 2022.07.15 | Guangdong | Duck | White Duck | 45 | 1 |
| 2022.07.15 | Guangdong | Duck | White Duck | 45 | 1 |
| 2022.07.15 | Jiangsu | Duck | White Duck | 26 | 1 |
| 2022.07.15 | Jiangsu | Duck | White Duck | 26 | 1 |
| 2022.07.15 | Jiangsu | Duck | White Duck | 26 | 1 |
| 2022.07.15 | Jiangsu | Duck | White Duck | 26 | 1 |
| 2022.07.18 | Hebei | Duck | Cherry Valley Duck | 7 | 1 |
| 2022.07.18 | Hebei | Duck | Cherry Valley Duck | 7 | 1 |
| 2022.07.18 | Henan | Duck | White Duck | 2 | 1 |
| 2022.07.18 | Henan | Duck | White Duck | 2 | 1 |
| 2022.07.18 | Shandong | Duck | Cherry Valley Duck | 7 | 1 |
| 2022.07.18 | Shandong | Duck | Cherry Valley Duck | 7 | 1 |
| 2022.07.18 | Shandong | Duck | Cherry Valley Duck | 7 | 1 |
| 2022.07.18 | Shandong | Duck | Cherry Valley Duck | 7 | 1 |
| 2022.07.18 | Shandong | Duck | Cherry Valley Duck | 31 | 1 |
| 2022.07.18 | Shandong | Duck | Cherry Valley Duck | 31 | 1 |
| 2022.07.18 | Shandong | Duck | Cherry Valley Duck | 31 | 1 |
| 2022.07.18 | Guangxi | Duck | Muscovy Duck | 400 | 1 |
| 2022.07.18 | Guangxi | Duck | Muscovy Duck | 400 | 1 |
| 2022.07.18 | Guangdong | Duck | White Duck | 35 | 1 |
| 2022.07.18 | Guangdong | Duck | White Duck | 35 | 1 |
| 2022.07.18 | Guangdong | Duck | White Duck | 35 | 1 |
| 2022.07.18 | Guangdong | Duck | White Duck | 35 | 1 |
| 2022.07.18 | Guangdong | Duck | White Duck | 35 | 1 |
| 2022.07.18 | Guangdong | Duck | White Duck | 35 | 1 |
| 2022.07.18 | Henan | Duck | / | 140 | 1 |
| 2022.07.18 | Henan | Duck | / | 140 | 1 |
| 2022.07.18 | Henan | Duck | / | 140 | 1 |
| 2022.07.19 | Shandong | Duck | White Duck | 1 | 1 |
| 2022.07.19 | Shandong | Duck | White Duck | 1 | 1 |
| 2022.07.19 | Shandong | Duck | White Duck | 1 | 1 |
| 2022.07.19 | Shandong | Duck | White Duck | 1 | 1 |
| 2022.07.19 | Shandong | Duck | White Duck | 1 | 1 |
| 2022.07.19 | Shandong | Duck | White Duck | 1 | 1 |
| 2022.07.19 | Shandong | Duck | White Duck | 1 | 1 |
| 2022.07.19 | Shandong | Duck | White Duck | 1 | 1 |
| 2022.07.19 | Shandong | Duck | White Duck | 1 | 1 |
| 2022.07.19 | Shandong | Duck | White Duck | 1 | 1 |
| 2022.07.19 | Shandong | Duck | White Duck | 1 | 1 |
| 2022.07.19 | Shandong | Duck | White Duck | 1 | 1 |
| 2022.07.19 | Shandong | Duck | White Duck | 1 | 1 |
| 2022.07.19 | Shandong | Duck | White Duck | 1 | 1 |
| 2022.07.19 | Shandong | Duck | White Duck | 1 | 1 |
| 2022.07.19 | Shandong | Duck | White Duck | 1 | 1 |
| 2022.07.19 | Shandong | Duck | White Duck | 1 | 1 |
| 2022.07.19 | Shandong | Duck | White Duck | 1 | 1 |
| 2022.07.19 | Shandong | Duck | White Duck | 1 | 1 |
| 2022.07.19 | Shandong | Duck | White Duck | 1 | 1 |
| 2022.07.19 | Shandong | Duck | White Duck | 1 | 1 |
| 2022.07.19 | Shandong | Duck | White Duck | 1 | 1 |
| 2022.07.19 | Shandong | Duck | White Duck | 1 | 1 |
| 2022.07.19 | Shandong | Duck | White Duck | 1 | 1 |
| 2022.07.19 | Shandong | Duck | White Duck | 1 | 1 |
| 2022.07.19 | Shandong | Duck | White Duck | 1 | 1 |
| 2022.07.19 | Shandong | Duck | White Duck | 1 | 1 |
| 2022.07.19 | Shandong | Duck | White Duck | 1 | 1 |
| 2022.07.19 | Shandong | Duck | White Duck | 1 | 1 |
| 2022.07.19 | Shandong | Duck | White Duck | 1 | 1 |
| 2022.07.19 | Shandong | Duck | White Duck | 1 | 1 |
| 2022.07.19 | Shandong | Duck | White Duck | 1 | 1 |
| 2022.07.19 | Shandong | Duck | White Duck | 1 | 1 |
| 2022.07.19 | Shandong | Duck | White Duck | 1 | 1 |
| 2022.07.19 | Shandong | Duck | White Duck | 1 | 1 |
| 2022.07.19 | Shandong | Duck | White Duck | 1 | 1 |
| 2022.07.19 | Shandong | Duck | White Duck | 1 | 1 |
| 2022.07.19 | Shandong | Duck | White Duck | 1 | 1 |
| 2022.07.19 | Shandong | Duck | White Duck | 1 | 1 |
| 2022.07.19 | Shandong | Duck | White Duck | 1 | 1 |
| 2022.07.19 | Shandong | Duck | White Duck | 1 | 1 |
| 2022.07.19 | Shandong | Duck | White Duck | 1 | 1 |
| 2022.07.19 | Shandong | Duck | White Duck | 1 | 1 |
| 2022.07.19 | Shandong | Duck | White Duck | 1 | 1 |
| 2022.07.20 | Shandong | Duck | Cherry Valley Duck | 1 | 1 |
| 2022.07.20 | Shandong | Duck | Cherry Valley Duck | 1 | 1 |
| 2022.07.20 | Shandong | Duck | Cherry Valley Duck | 1 | 1 |
| 2022.07.20 | Shandong | Duck | Cherry Valley Duck | 1 | 1 |
| 2022.07.20 | Shandong | Duck | Cherry Valley Duck | 1 | 1 |
| 2022.07.20 | Shandong | Duck | Cherry Valley Duck | 1 | 1 |
| 2022.07.20 | Shandong | Duck | Cherry Valley Duck | 1 | 1 |
| 2022.07.20 | Shandong | Duck | Cherry Valley Duck | 1 | 1 |
| 2022.07.20 | Shandong | Duck | Cherry Valley Duck | 1 | 1 |
| 2022.07.20 | Shandong | Duck | Cherry Valley Duck | 1 | 1 |
| 2022.07.20 | Shandong | Duck | Cherry Valley Duck | 1 | 1 |
| 2022.07.20 | Shandong | Duck | Cherry Valley Duck | 1 | 1 |
| 2022.07.20 | Shandong | Duck | Cherry Valley Duck | 1 | 1 |
| 2022.07.20 | Shandong | Duck | Cherry Valley Duck | 1 | 1 |
| 2022.07.20 | Shandong | Duck | Cherry Valley Duck | 1 | 1 |
| 2022.07.20 | Shandong | Duck | Cherry Valley Duck | 1 | 1 |
| 2022.07.20 | Shandong | Duck | Cherry Valley Duck | 1 | 1 |
| 2022.07.20 | Shandong | Duck | Cherry Valley Duck | 1 | 1 |
| 2022.07.20 | Shandong | Duck | Cherry Valley Duck | 1 | 1 |
| 2022.07.20 | Shandong | Duck | Cherry Valley Duck | 1 | 1 |
| 2022.07.20 | Shandong | Duck | Cherry Valley Duck | 1 | 1 |
| 2022.07.20 | Shandong | Duck | Cherry Valley Duck | 1 | 1 |
| 2022.07.20 | Shandong | Duck | Cherry Valley Duck | 1 | 1 |
| 2022.07.20 | Shandong | Duck | Cherry Valley Duck | 1 | 1 |
| 2022.07.20 | Shandong | Duck | Cherry Valley Duck | 1 | 1 |
| 2022.07.20 | Shandong | Duck | Cherry Valley Duck | 1 | 1 |
| 2022.07.20 | Shandong | Duck | Cherry Valley Duck | 1 | 1 |
| 2022.07.20 | Shandong | Duck | Cherry Valley Duck | 1 | 1 |
| 2022.07.20 | Shandong | Duck | Cherry Valley Duck | 1 | 1 |
| 2022.07.20 | Shandong | Duck | Cherry Valley Duck | 1 | 1 |
| 2022.07.20 | Shandong | Duck | Cherry Valley Duck | 1 | 1 |
| 2022.07.20 | Shandong | Duck | Cherry Valley Duck | 1 | 1 |
| 2022.07.20 | Shandong | Duck | Cherry Valley Duck | 32 | 1 |
| 2022.07.20 | Shandong | Duck | Cherry Valley Duck | 32 | 1 |
| 2022.07.20 | Shandong | Duck | Cherry Valley Duck | 32 | 1 |
| 2022.07.20 | Shandong | Duck | Cherry Valley Duck | 32 | 1 |
| 2022.07.20 | Shandong | Duck | Cherry Valley Duck | 32 | 1 |
| 2022.07.20 | Shandong | Duck | Cherry Valley Duck | 32 | 1 |
| 2022.07.20 | Shandong | Duck | Cherry Valley Duck | 32 | 1 |
| 2022.07.20 | Shandong | Duck | Cherry Valley Duck | 32 | 1 |
| 2022.07.20 | Shandong | Duck | Cherry Valley Duck | 32 | 1 |
| 2022.07.20 | Shandong | Duck | Cherry Valley Duck | 32 | 1 |
| 2022.07.20 | Shandong | Duck | Cherry Valley Duck | 32 | 1 |
| 2022.07.20 | Shandong | Duck | Cherry Valley Duck | 32 | 1 |
| 2022.07.20 | Shandong | Duck | Cherry Valley Duck | 32 | 1 |
| 2022.07.20 | Shandong | Duck | Cherry Valley Duck | 32 | 1 |
| 2022.07.20 | Shandong | Duck | Cherry Valley Duck | 32 | 1 |
| 2022.07.20 | Shandong | Duck | Cherry Valley Duck | 32 | 1 |
| 2022.07.20 | Hebei | Duck | Cherry Valley Duck | 2 | 1 |
| 2022.07.20 | Hebei | Duck | Cherry Valley Duck | 2 | 1 |
| 2022.07.20 | Hebei | Duck | Cherry Valley Duck | 2 | 1 |
| 2022.07.20 | Hebei | Duck | Cherry Valley Duck | 2 | 1 |
| 2022.07.20 | Hebei | Duck | Cherry Valley Duck | 2 | 1 |
| 2022.07.20 | Hebei | Duck | Cherry Valley Duck | 2 | 1 |
| 2022.07.20 | Shandong | Duck | / | 14 | 1 |
| 2022.07.20 | Anhui | Duck | Cherry Valley Duck | 38 | 1 |
| 2022.07.20 | Anhui | Duck | Cherry Valley Duck | 38 | 1 |
| 2022.07.20 | Guangdong | Duck | Muscovy Duck | 40 | 1 |
| 2022.07.20 | Guangdong | Duck | Muscovy Duck | 40 | 1 |
| 2022.07.20 | Guangdong | Duck | Muscovy Duck | 40 | 1 |
| 2022.07.20 | Guangdong | Duck | Muscovy Duck | 40 | 1 |
| 2022.07.20 | Guangdong | Duck | Muscovy Duck | 18 | 1 |
| 2022.07.20 | Guangdong | Duck | Muscovy Duck | 20 | 1 |
| 2022.07.20 | Guangdong | Duck | Muscovy Duck | 20 | 1 |
| 2022.07.20 | Guangdong | Duck | Muscovy Duck | 35 | 1 |
| 2022.07.20 | Guangdong | Duck | Muscovy Duck | 40 | 1 |
| 2022.07.20 | Guangdong | Duck | Muscovy Duck | 40 | 1 |
| 2022.07.20 | Guangdong | Duck | Muscovy Duck | 40 | 1 |
| 2022.07.20 | Guangdong | Duck | Muscovy Duck | 40 | 1 |
| 2022.07.20 | Guangdong | Duck | Muscovy Duck | 40 | 1 |
| 2022.07.21 | Hebei | Duck | White Duck | 2 | 1 |
| 2022.07.21 | Hebei | Duck | White Duck | 2 | 1 |
| 2022.07.21 | Hebei | Duck | White Duck | 2 | 1 |
| 2022.07.21 | Hebei | Duck | White Duck | 2 | 1 |
| 2022.07.21 | Hebei | Duck | White Duck | 2 | 1 |
| 2022.07.21 | Hebei | Duck | White Duck | 7 | 1 |
| 2022.07.21 | Hebei | Duck | White Duck | 7 | 1 |
| 2022.07.21 | Hebei | Duck | White Duck | 7 | 1 |
| 2022.07.21 | Hebei | Duck | White Duck | 7 | 1 |
| 2022.07.21 | Hebei | Duck | White Duck | 7 | 1 |
| 2022.07.21 | Hebei | Duck | White Duck | 3 | 1 |
| 2022.07.21 | Hebei | Duck | White Duck | 3 | 1 |
| 2022.07.21 | Hebei | Duck | White Duck | 3 | 1 |
| 2022.07.21 | Hebei | Duck | White Duck | 3 | 1 |
| 2022.07.21 | Hebei | Duck | White Duck | 3 | 1 |
| 2022.07.21 | Hebei | Duck | White Duck | 3 | 1 |
| 2022.07.21 | Hebei | Duck | White Duck | 3 | 1 |
| 2022.07.21 | Hebei | Duck | White Duck | 3 | 1 |
| 2022.07.21 | Hebei | Duck | White Duck | 3 | 1 |
| 2022.07.21 | Hebei | Duck | White Duck | 3 | 1 |
| 2022.07.21 | Shandong | Duck | Cherry Valley Duck | 2 | 1 |
| 2022.07.21 | Shandong | Duck | Cherry Valley Duck | 2 | 1 |
| 2022.07.21 | Shandong | Duck | Cherry Valley Duck | 2 | 1 |
| 2022.07.21 | Shandong | Duck | Cherry Valley Duck | 2 | 1 |
| 2022.07.21 | Shandong | Duck | Cherry Valley Duck | 2 | 1 |
| 2022.07.21 | Shandong | Duck | Cherry Valley Duck | 2 | 1 |
| 2022.07.21 | Shandong | Duck | Cherry Valley Duck | 2 | 1 |
| 2022.07.21 | Shandong | Duck | Cherry Valley Duck | 2 | 1 |
| 2022.07.21 | Shandong | Duck | Cherry Valley Duck | 2 | 1 |
| 2022.07.21 | Shandong | Duck | Cherry Valley Duck | 2 | 1 |
| 2022.07.21 | Shandong | Duck | Cherry Valley Duck | 2 | 1 |
| 2022.07.21 | Shandong | Duck | Cherry Valley Duck | 2 | 1 |
| 2022.07.21 | Shandong | Duck | Cherry Valley Duck | 2 | 1 |
| 2022.07.21 | Shandong | Duck | Cherry Valley Duck | 2 | 1 |
| 2022.07.21 | Shandong | Duck | Cherry Valley Duck | 2 | 1 |
| 2022.07.21 | Shandong | Duck | Cherry Valley Duck | 2 | 1 |
| 2022.07.21 | Shandong | Duck | Cherry Valley Duck | 2 | 1 |
| 2022.07.21 | Shandong | Duck | Cherry Valley Duck | 2 | 1 |
| 2022.07.21 | Shandong | Duck | Cherry Valley Duck | 2 | 1 |
| 2022.07.21 | Shandong | Duck | Cherry Valley Duck | 2 | 1 |
| 2022.07.21 | Shandong | Duck | Cherry Valley Duck | 2 | 1 |
| 2022.07.21 | Shandong | Duck | Cherry Valley Duck | 2 | 1 |
| 2022.07.21 | Shandong | Duck | Cherry Valley Duck | 2 | 1 |
| 2022.07.21 | Shandong | Duck | Cherry Valley Duck | 2 | 1 |
| 2022.07.21 | Shandong | Duck | / | 29 | 1 |
| 2022.07.21 | Shandong | Duck | / | 29 | 1 |
| 2022.07.21 | Shandong | Duck | Cherry Valley Duck | 105 | 1 |
| 2022.07.21 | Shandong | Duck | Cherry Valley Duck | 105 | 1 |
| 2022.07.21 | Shandong | Duck | Cherry Valley Duck | 105 | 1 |
| 2022.07.21 | Shandong | Duck | Cherry Valley Duck | 105 | 1 |
| 2022.07.21 | Shandong | Duck | Cherry Valley Duck | 105 | 1 |
| 2022.07.21 | Shandong | Duck | Cherry Valley Duck | 105 | 1 |
| 2022.07.21 | Shandong | Duck | Cherry Valley Duck | 105 | 1 |
| 2022.07.21 | Shandong | Duck | Cherry Valley Duck | 105 | 1 |
| 2022.07.21 | Shandong | Duck | Cherry Valley Duck | 105 | 1 |
| 2022.07.21 | Shandong | Duck | Cherry Valley Duck | 105 | 1 |
| 2022.07.21 | Shandong | Duck | Cherry Valley Duck | 105 | 1 |
| 2022.07.21 | Shandong | Duck | Cherry Valley Duck | 105 | 1 |
| 2022.07.21 | Shandong | Duck | Cherry Valley Duck | 105 | 1 |
| 2022.07.21 | Shandong | Duck | Cherry Valley Duck | 105 | 1 |
| 2022.07.21 | Shandong | Duck | Cherry Valley Duck | 105 | 1 |
| 2022.07.21 | Shandong | Duck | Cherry Valley Duck | 105 | 1 |
| 2022.07.21 | Shandong | Duck | Cherry Valley Duck | 105 | 1 |
| 2022.07.21 | Shandong | Duck | Cherry Valley Duck | 105 | 1 |
| 2022.07.21 | Shandong | Duck | Cherry Valley Duck | 105 | 1 |
| 2022.07.21 | Shandong | Duck | Cherry Valley Duck | 105 | 1 |
| 2022.07.21 | Shandong | Duck | Cherry Valley Duck | 105 | 1 |
| 2022.07.21 | Shandong | Duck | Cherry Valley Duck | 105 | 1 |
| 2022.07.21 | Shandong | Duck | Cherry Valley Duck | 105 | 1 |
| 2022.07.21 | Shandong | Duck | Cherry Valley Duck | 105 | 1 |
| 2022.07.21 | Shandong | Duck | Cherry Valley Duck | 105 | 1 |
| 2022.07.21 | Shandong | Duck | Cherry Valley Duck | 105 | 1 |
| 2022.07.21 | Shandong | Duck | Cherry Valley Duck | 105 | 1 |
| 2022.07.21 | Shandong | Duck | Cherry Valley Duck | 105 | 1 |
| 2022.07.21 | Shandong | Duck | Cherry Valley Duck | 105 | 1 |
| 2022.07.21 | Shandong | Duck | Cherry Valley Duck | 105 | 1 |
| 2022.07.21 | Shandong | Duck | Cherry Valley Duck | 105 | 1 |
| 2022.07.21 | Shandong | Duck | Cherry Valley Duck | 105 | 1 |
| 2022.07.21 | Guangdong | Duck | White Duck | 25 | 1 |
| 2022.07.21 | Guangdong | Duck | White Duck | 25 | 1 |
| 2022.07.21 | Jiangxi | Duck | Muscovy Duck | 23 | 1 |
| 2022.07.21 | Jiangxi | Duck | Muscovy Duck | 23 | 1 |
| 2022.07.21 | Jiangxi | Duck | Muscovy Duck | 23 | 1 |
| 2022.07.22 | Anhui | Duck | / | / | 1 |
| 2022.07.22 | Anhui | Duck | / | / | 1 |
| 2022.07.25 | Guangdong | Duck | Cherry Valley Duck | 32 | 1 |
| 2022.07.25 | Guangdong | Duck | Cherry Valley Duck | 32 | 1 |
| 2022.07.25 | Guangdong | Duck | Cherry Valley Duck | 32 | 1 |
| 2022.07.25 | Guangxi | Duck | Cherry Valley Duck | 15 | 1 |
| 2022.07.25 | Guangxi | Duck | Cherry Valley Duck | 15 | 1 |
| 2022.07.25 | Guangxi | Duck | Cherry Valley Duck | 15 | 1 |
| 2022.07.25 | Guangxi | Duck | Cherry Valley Duck | 15 | 1 |
| 2022.07.25 | Guangxi | Duck | Cherry Valley Duck | 15 | 1 |
| 2022.07.25 | Guangxi | Duck | Cherry Valley Duck | 15 | 1 |
| 2022.07.26 | Nei Mongol | Duck | / | 32 | 1 |
| 2022.07.26 | Nei Mongol | Duck | / | 32 | 1 |
| 2022.07.26 | Nei Mongol | Duck | / | 32 | 1 |
| 2022.07.26 | Nei Mongol | Duck | / | 32 | 1 |
| 2022.07.26 | Nei Mongol | Duck | / | 32 | 1 |
| 2022.07.26 | Shandong | Duck | Cherry Valley Duck | 1 | 1 |
| 2022.07.26 | Shandong | Duck | Cherry Valley Duck | 1 | 1 |
| 2022.07.26 | Shandong | Duck | Cherry Valley Duck | 1 | 1 |
| 2022.07.26 | Shandong | Duck | Cherry Valley Duck | 1 | 1 |
| 2022.07.26 | Shandong | Duck | Cherry Valley Duck | 1 | 1 |
| 2022.07.26 | Shandong | Duck | Cherry Valley Duck | 1 | 1 |
| 2022.07.26 | Shandong | Duck | Cherry Valley Duck | 1 | 1 |
| 2022.07.26 | Shandong | Duck | Cherry Valley Duck | 1 | 1 |
| 2022.07.26 | Shandong | Duck | Cherry Valley Duck | 1 | 1 |
| 2022.07.26 | Shandong | Duck | Cherry Valley Duck | 1 | 1 |
| 2022.07.26 | Shandong | Duck | Cherry Valley Duck | 1 | 1 |
| 2022.07.26 | Shandong | Duck | Cherry Valley Duck | 1 | 1 |
| 2022.07.26 | Shandong | Duck | Cherry Valley Duck | 1 | 1 |
| 2022.07.26 | Shandong | Duck | Cherry Valley Duck | 1 | 1 |
| 2022.07.26 | Shandong | Duck | Cherry Valley Duck | 1 | 1 |
| 2022.07.26 | Shandong | Duck | Cherry Valley Duck | 1 | 1 |
| 2022.07.26 | Shandong | Duck | Cherry Valley Duck | 1 | 1 |
| 2022.07.26 | Shandong | Duck | Cherry Valley Duck | 1 | 1 |
| 2022.07.26 | Shandong | Duck | Cherry Valley Duck | 1 | 1 |
| 2022.07.26 | Shandong | Duck | Cherry Valley Duck | 1 | 1 |
| 2022.07.26 | Shandong | Duck | Cherry Valley Duck | 1 | 1 |
| 2022.07.26 | Shandong | Duck | Cherry Valley Duck | 1 | 1 |
| 2022.07.26 | Shandong | Duck | Cherry Valley Duck | 1 | 1 |
| 2022.07.26 | Shandong | Duck | Cherry Valley Duck | 1 | 1 |
| 2022.07.26 | Shandong | Duck | Cherry Valley Duck | 1 | 1 |
| 2022.07.26 | Shandong | Duck | Cherry Valley Duck | 1 | 1 |
| 2022.07.26 | Shandong | Duck | Cherry Valley Duck | 1 | 1 |
| 2022.07.26 | Shandong | Duck | Cherry Valley Duck | 1 | 1 |
| 2022.07.26 | Shandong | Duck | Cherry Valley Duck | 1 | 1 |
| 2022.07.26 | Shandong | Duck | Cherry Valley Duck | 1 | 1 |
| 2022.07.26 | Shandong | Duck | Cherry Valley Duck | 1 | 1 |
| 2022.07.26 | Shandong | Duck | Cherry Valley Duck | 1 | 1 |
| 2022.07.26 | Shandong | Duck | Cherry Valley Duck | 1 | 1 |
| 2022.07.26 | Shandong | Duck | Cherry Valley Duck | 1 | 1 |
| 2022.07.26 | Shandong | Duck | Cherry Valley Duck | 1 | 1 |
| 2022.07.26 | Shandong | Duck | Cherry Valley Duck | 1 | 1 |
| 2022.07.26 | Shandong | Duck | Cherry Valley Duck | 1 | 1 |
| 2022.07.26 | Shandong | Duck | Cherry Valley Duck | 1 | 1 |
| 2022.07.26 | Shandong | Duck | Cherry Valley Duck | 1 | 1 |
| 2022.07.26 | Shandong | Duck | Cherry Valley Duck | 1 | 1 |
| 2022.07.26 | Shandong | Duck | Cherry Valley Duck | 1 | 1 |
| 2022.07.26 | Shandong | Duck | Cherry Valley Duck | 1 | 1 |
| 2022.07.26 | Shandong | Duck | Cherry Valley Duck | 1 | 1 |
| 2022.07.26 | Shandong | Duck | Cherry Valley Duck | 1 | 1 |
| 2022.07.26 | Shandong | Duck | Cherry Valley Duck | 1 | 1 |
| 2022.07.26 | Shandong | Duck | Cherry Valley Duck | 1 | 1 |
| 2022.07.26 | Shandong | Duck | Cherry Valley Duck | 1 | 1 |
| 2022.07.26 | Shandong | Duck | Cherry Valley Duck | 1 | 1 |
| 2022.07.26 | Shandong | Duck | Cherry Valley Duck | 1 | 1 |
| 2022.07.26 | Shandong | Duck | Cherry Valley Duck | 1 | 1 |
| 2022.07.26 | Shandong | Duck | Cherry Valley Duck | 1 | 1 |
| 2022.07.26 | Shandong | Duck | Cherry Valley Duck | 1 | 1 |
| 2022.07.26 | Shandong | Duck | Cherry Valley Duck | 1 | 1 |
| 2022.07.26 | Shandong | Duck | Cherry Valley Duck | 1 | 1 |
| 2022.07.26 | Shandong | Duck | Cherry Valley Duck | 1 | 1 |
| 2022.07.26 | Shandong | Duck | Cherry Valley Duck | 1 | 1 |
| 2022.07.26 | Shandong | Duck | Cherry Valley Duck | 1 | 1 |
| 2022.07.26 | Shandong | Duck | Cherry Valley Duck | 1 | 1 |
| 2022.07.26 | Shandong | Duck | Cherry Valley Duck | 1 | 1 |
| 2022.07.26 | Shandong | Duck | Cherry Valley Duck | 1 | 1 |
| 2022.07.26 | Shandong | Duck | Cherry Valley Duck | 1 | 1 |
| 2022.07.26 | Shandong | Duck | Cherry Valley Duck | 1 | 1 |
| 2022.07.26 | Shandong | Duck | Cherry Valley Duck | 1 | 1 |
| 2022.07.26 | Shandong | Duck | Cherry Valley Duck | 1 | 1 |
| 2022.07.26 | Shandong | Duck | Cherry Valley Duck | 1 | 1 |
| 2022.07.26 | Shandong | Duck | Cherry Valley Duck | 1 | 1 |
| 2022.07.26 | Shandong | Duck | Cherry Valley Duck | 1 | 1 |
| 2022.07.26 | Shandong | Duck | Cherry Valley Duck | 1 | 1 |
| 2022.07.26 | Shandong | Duck | Cherry Valley Duck | 1 | 1 |
| 2022.07.26 | Shandong | Duck | Cherry Valley Duck | 1 | 1 |
| 2022.07.26 | Shandong | Duck | Cherry Valley Duck | 1 | 1 |
| 2022.07.26 | Shandong | Duck | Cherry Valley Duck | 1 | 1 |
| 2022.07.26 | Shandong | Duck | Cherry Valley Duck | 1 | 1 |
| 2022.07.26 | Shandong | Duck | Cherry Valley Duck | 1 | 1 |
| 2022.07.26 | Shandong | Duck | Cherry Valley Duck | 1 | 1 |
| 2022.07.26 | Shandong | Duck | Cherry Valley Duck | 1 | 1 |
| 2022.07.26 | Shandong | Duck | Cherry Valley Duck | 1 | 1 |
| 2022.07.26 | Shandong | Duck | Cherry Valley Duck | 1 | 1 |
| 2022.07.26 | Shandong | Duck | Cherry Valley Duck | 1 | 1 |
| 2022.07.26 | Shandong | Duck | Cherry Valley Duck | 1 | 1 |
| 2022.07.26 | Shandong | Duck | Cherry Valley Duck | 1 | 1 |
| 2022.07.26 | Shandong | Duck | Cherry Valley Duck | 1 | 1 |
| 2022.07.26 | Shandong | Duck | Cherry Valley Duck | 1 | 1 |
| 2022.07.26 | Shandong | Duck | Cherry Valley Duck | 1 | 1 |
| 2022.07.26 | Shandong | Duck | Cherry Valley Duck | 1 | 1 |
| 2022.07.26 | Shandong | Duck | Cherry Valley Duck | 1 | 1 |
| 2022.07.26 | Shandong | Duck | Cherry Valley Duck | 1 | 1 |
| 2022.07.26 | Shandong | Duck | Cherry Valley Duck | 1 | 1 |
| 2022.07.26 | Shandong | Duck | Cherry Valley Duck | 1 | 1 |
| 2022.07.26 | Shandong | Duck | Cherry Valley Duck | 1 | 1 |
| 2022.07.26 | Shandong | Duck | Cherry Valley Duck | 1 | 1 |
| 2022.07.26 | Shandong | Duck | Cherry Valley Duck | 1 | 1 |
| 2022.07.26 | Shandong | Duck | Cherry Valley Duck | 1 | 1 |
| 2022.07.26 | Shandong | Duck | Cherry Valley Duck | 1 | 1 |
| 2022.07.26 | Shandong | Duck | Cherry Valley Duck | 1 | 1 |
| 2022.07.26 | Shandong | Duck | Cherry Valley Duck | 1 | 1 |
| 2022.07.26 | Shandong | Duck | Cherry Valley Duck | 1 | 1 |
| 2022.07.26 | Shandong | Duck | Cherry Valley Duck | 1 | 1 |
| 2022.07.26 | Shandong | Duck | Cherry Valley Duck | 1 | 1 |
| 2022.07.26 | Shandong | Duck | Cherry Valley Duck | 1 | 1 |
| 2022.07.26 | Shandong | Duck | Cherry Valley Duck | 1 | 1 |
| 2022.07.26 | Shandong | Duck | Cherry Valley Duck | 1 | 1 |
| 2022.07.26 | Shandong | Duck | Cherry Valley Duck | 1 | 1 |
| 2022.07.26 | Shandong | Duck | Cherry Valley Duck | 1 | 1 |
| 2022.07.26 | Shandong | Duck | Cherry Valley Duck | 1 | 1 |
| 2022.07.26 | Shandong | Duck | Cherry Valley Duck | 1 | 1 |
| 2022.07.26 | Shandong | Duck | Cherry Valley Duck | 1 | 1 |
| 2022.07.26 | Shandong | Duck | Cherry Valley Duck | 1 | 1 |
| 2022.07.26 | Shandong | Duck | Cherry Valley Duck | 1 | 1 |
| 2022.07.26 | Shandong | Duck | Cherry Valley Duck | 1 | 1 |
| 2022.07.26 | Shandong | Duck | Cherry Valley Duck | 1 | 1 |
| 2022.07.26 | Shandong | Duck | Cherry Valley Duck | 1 | 1 |
| 2022.07.26 | Shandong | Duck | Cherry Valley Duck | 1 | 1 |
| 2022.07.26 | Shandong | Duck | Cherry Valley Duck | 1 | 1 |
| 2022.07.26 | Shandong | Duck | Cherry Valley Duck | 1 | 1 |
| 2022.07.26 | Shandong | Duck | Cherry Valley Duck | 1 | 1 |
| 2022.07.26 | Shandong | Duck | Cherry Valley Duck | 1 | 1 |
| 2022.07.26 | Shandong | Duck | Cherry Valley Duck | 1 | 1 |
| 2022.07.26 | Shandong | Duck | Cherry Valley Duck | 1 | 1 |
| 2022.07.26 | Shandong | Duck | Cherry Valley Duck | 1 | 1 |
| 2022.07.26 | Shandong | Duck | Cherry Valley Duck | 1 | 1 |
| 2022.07.26 | Shandong | Duck | Cherry Valley Duck | 1 | 1 |
| 2022.07.26 | Shandong | Duck | Cherry Valley Duck | 1 | 1 |
| 2022.07.26 | Shandong | Duck | Cherry Valley Duck | 1 | 1 |
| 2022.07.26 | Shandong | Duck | Cherry Valley Duck | 1 | 1 |
| 2022.07.26 | Shandong | Duck | Cherry Valley Duck | 1 | 1 |
| 2022.07.26 | Shandong | Duck | Cherry Valley Duck | 1 | 1 |
| 2022.07.26 | Shandong | Duck | Cherry Valley Duck | 1 | 1 |
| 2022.07.26 | Shandong | Duck | Cherry Valley Duck | 1 | 1 |
| 2022.07.26 | Shandong | Duck | Cherry Valley Duck | 1 | 1 |
| 2022.07.26 | Shandong | Duck | Cherry Valley Duck | 1 | 1 |
| 2022.07.26 | Shandong | Duck | Cherry Valley Duck | 1 | 1 |
| 2022.07.26 | Shandong | Duck | Cherry Valley Duck | 1 | 1 |
| 2022.07.26 | Shandong | Duck | Cherry Valley Duck | 1 | 1 |
| 2022.07.26 | Shandong | Duck | Cherry Valley Duck | 1 | 1 |
| 2022.07.26 | Shandong | Duck | Cherry Valley Duck | 1 | 1 |
| 2022.07.26 | Shandong | Duck | Cherry Valley Duck | 1 | 1 |
| 2022.07.26 | Shandong | Duck | Cherry Valley Duck | 1 | 1 |
| 2022.07.26 | Shandong | Duck | Cherry Valley Duck | 1 | 1 |
| 2022.07.26 | Shandong | Duck | Cherry Valley Duck | 1 | 1 |
| 2022.07.26 | Shandong | Duck | Cherry Valley Duck | 1 | 1 |
| 2022.07.26 | Shandong | Duck | Cherry Valley Duck | 1 | 1 |
| 2022.07.26 | Shandong | Duck | Cherry Valley Duck | 1 | 1 |
| 2022.07.26 | Shandong | Duck | Cherry Valley Duck | 1 | 1 |
| 2022.07.26 | Shandong | Duck | Cherry Valley Duck | 1 | 1 |
| 2022.07.26 | Shandong | Duck | Cherry Valley Duck | 1 | 1 |
| 2022.07.26 | Shandong | Duck | Cherry Valley Duck | 1 | 1 |
| 2022.07.26 | Shandong | Duck | Cherry Valley Duck | 1 | 1 |
| 2022.07.26 | Shandong | Duck | Cherry Valley Duck | 1 | 1 |
| 2022.07.26 | Shandong | Duck | Cherry Valley Duck | 1 | 1 |
| 2022.07.26 | Shandong | Duck | Cherry Valley Duck | 1 | 1 |
| 2022.07.26 | Shandong | Duck | Cherry Valley Duck | 1 | 1 |
| 2022.07.26 | Shandong | Duck | Cherry Valley Duck | 1 | 1 |
| 2022.07.26 | Shandong | Duck | Cherry Valley Duck | 1 | 1 |
| 2022.07.26 | Shandong | Duck | Cherry Valley Duck | 1 | 1 |
| 2022.07.26 | Shandong | Duck | Cherry Valley Duck | 1 | 1 |
| 2022.07.26 | Shandong | Duck | Cherry Valley Duck | 56 | 1 |
| 2022.07.26 | Guangdong | Duck | Mule Duck | 26 | 1 |
| 2022.07.26 | Guangdong | Duck | Mule Duck | 26 | 1 |
| 2022.07.26 | Guangdong | Duck | Mule Duck | 26 | 1 |
| 2022.07.26 | Guangdong | Duck | Mule Duck | 26 | 1 |
| 2022.07.26 | Guangdong | Duck | White Duck | 33 | 1 |
| 2022.07.27 | Shandong | Duck | Cherry Valley Duck | 1 | 1 |
| 2022.07.27 | Shandong | Duck | Cherry Valley Duck | 1 | 1 |
| 2022.07.27 | Shandong | Duck | Cherry Valley Duck | 1 | 1 |
| 2022.07.27 | Shandong | Duck | Cherry Valley Duck | 1 | 1 |
| 2022.07.27 | Shandong | Duck | Cherry Valley Duck | 1 | 1 |
| 2022.07.27 | Shandong | Duck | Cherry Valley Duck | 1 | 1 |
| 2022.07.27 | Shandong | Duck | Cherry Valley Duck | 1 | 1 |
| 2022.07.27 | Shandong | Duck | Cherry Valley Duck | 1 | 1 |
| 2022.07.27 | Shandong | Duck | Cherry Valley Duck | 1 | 1 |
| 2022.07.27 | Shandong | Duck | Cherry Valley Duck | 1 | 1 |
| 2022.07.27 | Shandong | Duck | Cherry Valley Duck | 1 | 1 |
| 2022.07.27 | Shandong | Duck | Cherry Valley Duck | 1 | 1 |
| 2022.07.27 | Shandong | Duck | Cherry Valley Duck | 1 | 1 |
| 2022.07.27 | Shandong | Duck | Cherry Valley Duck | 1 | 1 |
| 2022.07.27 | Shandong | Duck | Cherry Valley Duck | 1 | 1 |
| 2022.07.27 | Shandong | Duck | Cherry Valley Duck | 1 | 1 |
| 2022.07.27 | Shandong | Duck | Cherry Valley Duck | 1 | 1 |
| 2022.07.27 | Shandong | Duck | Cherry Valley Duck | 1 | 1 |
| 2022.07.27 | Shandong | Duck | Cherry Valley Duck | 1 | 1 |
| 2022.07.27 | Shandong | Duck | Cherry Valley Duck | 1 | 1 |
| 2022.07.27 | Shandong | Duck | Cherry Valley Duck | 1 | 1 |
| 2022.07.27 | Shandong | Duck | Cherry Valley Duck | 1 | 1 |
| 2022.07.27 | Shandong | Duck | Cherry Valley Duck | 1 | 1 |
| 2022.07.27 | Shandong | Duck | Cherry Valley Duck | 1 | 1 |
| 2022.07.27 | Shandong | Duck | Cherry Valley Duck | 1 | 1 |
| 2022.07.27 | Shandong | Duck | Cherry Valley Duck | 1 | 1 |
| 2022.07.27 | Shandong | Duck | Cherry Valley Duck | 1 | 1 |
| 2022.07.27 | Shandong | Duck | Cherry Valley Duck | 1 | 1 |
| 2022.07.27 | Shandong | Duck | Cherry Valley Duck | 1 | 1 |
| 2022.07.27 | Shandong | Duck | Cherry Valley Duck | 1 | 1 |
| 2022.07.27 | Shandong | Duck | Cherry Valley Duck | 1 | 1 |
| 2022.07.27 | Shandong | Duck | Cherry Valley Duck | 1 | 1 |
| 2022.07.27 | Shandong | Duck | Cherry Valley Duck | 1 | 1 |
| 2022.07.27 | Shandong | Duck | Cherry Valley Duck | 1 | 1 |
| 2022.07.27 | Shandong | Duck | Cherry Valley Duck | 1 | 1 |
| 2022.07.27 | Shandong | Duck | Cherry Valley Duck | 1 | 1 |
| 2022.07.27 | Shandong | Duck | Cherry Valley Duck | 1 | 1 |
| 2022.07.27 | Shandong | Duck | Cherry Valley Duck | 1 | 1 |
| 2022.07.27 | Shandong | Duck | Cherry Valley Duck | 1 | 1 |
| 2022.07.27 | Shandong | Duck | Cherry Valley Duck | 1 | 1 |
| 2022.07.27 | Shandong | Duck | Cherry Valley Duck | 1 | 1 |
| 2022.07.27 | Shandong | Duck | Cherry Valley Duck | 1 | 1 |
| 2022.07.27 | Shandong | Duck | Cherry Valley Duck | 1 | 1 |
| 2022.07.27 | Shandong | Duck | Cherry Valley Duck | 1 | 1 |
| 2022.07.27 | Shandong | Duck | Cherry Valley Duck | 1 | 1 |
| 2022.07.27 | Shandong | Duck | Cherry Valley Duck | 1 | 1 |
| 2022.07.27 | Shandong | Duck | Cherry Valley Duck | 1 | 1 |
| 2022.07.27 | Shandong | Duck | Cherry Valley Duck | 1 | 1 |
| 2022.07.27 | Shandong | Duck | Cherry Valley Duck | 1 | 1 |
| 2022.07.27 | Shandong | Duck | Cherry Valley Duck | 1 | 1 |
| 2022.07.27 | Shandong | Duck | Cherry Valley Duck | 1 | 1 |
| 2022.07.27 | Shandong | Duck | Cherry Valley Duck | 1 | 1 |
| 2022.07.27 | Shandong | Duck | Cherry Valley Duck | 1 | 1 |
| 2022.07.27 | Shandong | Duck | Cherry Valley Duck | 1 | 1 |
| 2022.07.27 | Shandong | Duck | Cherry Valley Duck | 1 | 1 |
| 2022.07.27 | Shandong | Duck | Cherry Valley Duck | 1 | 1 |
| 2022.07.27 | Shandong | Duck | Cherry Valley Duck | 1 | 1 |
| 2022.07.27 | Shandong | Duck | Cherry Valley Duck | 1 | 1 |
| 2022.07.27 | Shandong | Duck | Cherry Valley Duck | 1 | 1 |
| 2022.07.27 | Shandong | Duck | Cherry Valley Duck | 1 | 1 |
| 2022.07.27 | Shandong | Duck | Cherry Valley Duck | 1 | 1 |
| 2022.07.27 | Shandong | Duck | Cherry Valley Duck | 1 | 1 |
| 2022.07.27 | Shandong | Duck | Cherry Valley Duck | 1 | 1 |
| 2022.07.27 | Shandong | Duck | Cherry Valley Duck | 1 | 1 |
| 2022.07.27 | Shandong | Duck | Cherry Valley Duck | 1 | 1 |
| 2022.07.27 | Shandong | Duck | Cherry Valley Duck | 1 | 1 |
| 2022.07.27 | Shandong | Duck | Cherry Valley Duck | 1 | 1 |
| 2022.07.27 | Shandong | Duck | Cherry Valley Duck | 1 | 1 |
| 2022.07.27 | Shandong | Duck | Cherry Valley Duck | 1 | 1 |
| 2022.07.27 | Shandong | Duck | Cherry Valley Duck | 1 | 1 |
| 2022.07.27 | Shandong | Duck | Cherry Valley Duck | 1 | 1 |
| 2022.07.27 | Shandong | Duck | Cherry Valley Duck | 1 | 1 |
| 2022.07.27 | Guangdong | Duck | Muscovy Duck | 24 | 1 |
| 2022.07.27 | Guangdong | Duck | Muscovy Duck | 24 | 1 |
| 2022.07.27 | Guangdong | Duck | Muscovy Duck | 24 | 1 |
| 2022.07.27 | Guangdong | Duck | White Duck | 25 | 1 |
| 2022.07.27 | Guangdong | Duck | White Duck | 35 | 1 |
| 2022.07.27 | Guangdong | Duck | / | 200 | 1 |
| 2022.07.27 | Guangdong | Duck | / | 300 | 1 |
| 2022.07.28 | Shandong | Duck | / | 21 | 1 |
| 2022.07.28 | Shandong | Duck | / | 21 | 1 |
| 2022.07.28 | Jiangxi | Duck | Muscovy Duck | 23 | 1 |
| 2022.07.29 | Shandong | Duck | Cherry Valley Duck | 6 | 1 |
| 2022.07.29 | Shandong | Duck | Cherry Valley Duck | 6 | 1 |
| 2022.07.29 | Shandong | Duck | Cherry Valley Duck | 6 | 1 |
| 2022.07.29 | Shandong | Duck | Cherry Valley Duck | 6 | 1 |
| 2022.07.29 | Shandong | Duck | Cherry Valley Duck | 6 | 1 |
| 2022.07.29 | Shandong | Duck | Cherry Valley Duck | 6 | 1 |
| 2022.07.29 | Shandong | Duck | Cherry Valley Duck | 6 | 1 |
| 2022.07.29 | Shandong | Duck | Cherry Valley Duck | 6 | 1 |
| 2022.07.29 | Shandong | Duck | Cherry Valley Duck | 6 | 1 |
| 2022.07.29 | Shandong | Duck | Cherry Valley Duck | 6 | 1 |
| 2022.07.29 | Shandong | Duck | Cherry Valley Duck | 6 | 1 |
| 2022.07.29 | Shandong | Duck | Cherry Valley Duck | 6 | 1 |
| 2022.07.29 | Shandong | Duck | Cherry Valley Duck | 6 | 1 |
| 2022.07.29 | Shandong | Duck | Cherry Valley Duck | 6 | 1 |
| 2022.07.29 | Shandong | Duck | Cherry Valley Duck | 6 | 1 |
| 2022.07.29 | Shandong | Duck | Cherry Valley Duck | 6 | 1 |
| 2022.07.29 | Shandong | Duck | Cherry Valley Duck | 6 | 1 |
| 2022.07.29 | Shandong | Duck | Cherry Valley Duck | 6 | 1 |
| 2022.07.29 | Shandong | Duck | Cherry Valley Duck | 6 | 1 |
| 2022.07.29 | Shandong | Duck | Cherry Valley Duck | 6 | 1 |
| 2022.07.29 | Shandong | Duck | Cherry Valley Duck | 6 | 1 |
| 2022.07.29 | Shandong | Duck | Cherry Valley Duck | 6 | 1 |
| 2022.07.29 | Shandong | Duck | Cherry Valley Duck | 6 | 1 |
| 2022.07.29 | Shandong | Duck | Cherry Valley Duck | 6 | 1 |
| 2022.07.29 | Guangdong | Duck | White Duck | 27 | 1 |
| 2022.07.29 | Guangdong | Duck | White Duck | 27 | 1 |
| 2022.07.29 | Guangdong | Duck | White Duck | 27 | 1 |
| 2022.07.29 | Guangdong | Duck | White Duck | 27 | 1 |
| 2022.07.29 | Guangdong | Duck | White Duck | 27 | 1 |
| 2022.07.29 | Guangdong | Duck | White Duck | 27 | 1 |
| 2022.07.29 | Guangdong | Duck | White Duck | 27 | 1 |
| 2022.07.29 | Fujian | Duck | Muscovy Duck | 40 | 1 |
| 2022.07.29 | Fujian | Duck | Muscovy Duck | 40 | 1 |
| 2022.07.29 | Fujian | Duck | Muscovy Duck | 40 | 1 |
| 2022.07.29 | Fujian | Duck | Muscovy Duck | 40 | 1 |
| 2022.07.29 | Fujian | Duck | Muscovy Duck | 40 | 1 |
| 2022.07.29 | Fujian | Duck | Muscovy Duck | 40 | 1 |
| 2022.07.29 | Fujian | Duck | Muscovy Duck | 40 | 1 |
| 2022.07.29 | Fujian | Duck | Muscovy Duck | 40 | 1 |
| 2022.07.29 | Fujian | Duck | Muscovy Duck | 40 | 1 |
| 2022.07.29 | Fujian | Duck | Muscovy Duck | 40 | 1 |
| 2022.07.29 | Fujian | Duck | Muscovy Duck | 40 | 1 |
| 2022.07.29 | Fujian | Duck | Muscovy Duck | 40 | 1 |
| 2022.07.30 | Shandong | Duck | Cherry Valley Duck | 28 | 1 |
| 2022.07.30 | Shandong | Duck | Cherry Valley Duck | 30 | 1 |
| 2022.07.30 | Guangdong | Duck | White Duck | 280 | 1 |
| 2022.07.30 | Guangdong | Duck | White Duck | 280 | 1 |
| 2022.07.30 | Guangdong | Duck | White Duck | 280 | 1 |
| 2022.07.30 | Guangdong | Duck | Shelduck | 280 | 1 |
| 2022.07.30 | Guangdong | Duck | White Duck | 280 | 1 |
| 2022.08.01 | Shandong | Duck | / | 27 | 1 |
| 2022.08.01 | Shandong | Duck | / | 24 | 1 |
| 2022.08.01 | Shandong | Duck | / | 34 | 1 |
| 2022.08.01 | Shandong | Duck | / | 26 | 1 |
| 2022.08.01 | Shandong | Duck | Cherry Valley Duck | 6 | 1 |
| 2022.08.01 | Shandong | Duck | Cherry Valley Duck | 6 | 1 |
| 2022.08.01 | Shandong | Duck | Cherry Valley Duck | 6 | 1 |
| 2022.08.01 | Shandong | Duck | Cherry Valley Duck | 6 | 1 |
| 2022.08.01 | Shandong | Duck | Cherry Valley Duck | 6 | 1 |
| 2022.08.01 | Shandong | Duck | Cherry Valley Duck | 6 | 1 |
| 2022.08.01 | Shandong | Duck | Cherry Valley Duck | 6 | 1 |
| 2022.08.01 | Shandong | Duck | Cherry Valley Duck | 6 | 1 |
| 2022.08.01 | Shandong | Duck | Cherry Valley Duck | 6 | 1 |
| 2022.08.01 | Shandong | Duck | Cherry Valley Duck | 6 | 1 |
| 2022.08.01 | Shandong | Duck | Cherry Valley Duck | 6 | 1 |
| 2022.08.01 | Shandong | Duck | Cherry Valley Duck | 6 | 1 |
| 2022.08.01 | Shandong | Duck | Cherry Valley Duck | 6 | 1 |
| 2022.08.01 | Shandong | Duck | Cherry Valley Duck | 6 | 1 |
| 2022.08.01 | Shandong | Duck | Cherry Valley Duck | 6 | 1 |
| 2022.08.01 | Shandong | Duck | Cherry Valley Duck | 6 | 1 |
| 2022.08.01 | Shandong | Duck | Cherry Valley Duck | 6 | 1 |
| 2022.08.01 | Shandong | Duck | Cherry Valley Duck | 6 | 1 |
| 2022.08.01 | Shandong | Duck | Cherry Valley Duck | 6 | 1 |
| 2022.08.01 | Shandong | Duck | Cherry Valley Duck | 6 | 1 |
| 2022.08.01 | Shandong | Duck | Cherry Valley Duck | 6 | 1 |
| 2022.08.01 | Shandong | Duck | Cherry Valley Duck | 6 | 1 |
| 2022.08.01 | Shandong | Duck | Cherry Valley Duck | 6 | 1 |
| 2022.08.01 | Shandong | Duck | Cherry Valley Duck | 6 | 1 |
| 2022.08.01 | Shandong | Duck | Cherry Valley Duck | 6 | 1 |
| 2022.08.01 | Shandong | Duck | Cherry Valley Duck | 6 | 1 |
| 2022.08.01 | Shandong | Duck | Cherry Valley Duck | 6 | 1 |
| 2022.08.01 | Shandong | Duck | Cherry Valley Duck | 6 | 1 |
| 2022.08.01 | Shandong | Duck | Cherry Valley Duck | 6 | 1 |
| 2022.08.01 | Shandong | Duck | Cherry Valley Duck | 6 | 1 |
| 2022.08.01 | Shandong | Duck | Cherry Valley Duck | 6 | 1 |
| 2022.08.01 | Shandong | Duck | Cherry Valley Duck | 6 | 1 |
| 2022.08.01 | Shandong | Duck | Cherry Valley Duck | 6 | 1 |
| 2022.08.01 | Shandong | Duck | Cherry Valley Duck | 6 | 1 |
| 2022.08.01 | Shandong | Duck | Cherry Valley Duck | 6 | 1 |
| 2022.08.01 | Shandong | Duck | Cherry Valley Duck | 6 | 1 |
| 2022.08.03 | Shandong | Duck | / | 31 | 1 |
| 2022.08.03 | Shandong | Duck | / | 35 | 1 |
| 2022.08.03 | Jiangxi | Duck | Muscovy Duck | 16 | 1 |
| 2022.08.03 | Jiangxi | Duck | Muscovy Duck | 16 | 1 |
| 2022.08.03 | Guangdong | Duck | Cherry Valley Duck | 40 | 1 |
| 2022.08.03 | Guangdong | Duck | Cherry Valley Duck | 40 | 1 |
| 2022.08.03 | Guangdong | Duck | Cherry Valley Duck | 40 | 1 |
| 2022.08.03 | Guangdong | Duck | Cherry Valley Duck | 40 | 1 |
| 2022.08.04 | Shandong | Duck | / | 31 | 1 |
| 2022.08.04 | Shandong | Duck | Cherry Valley Duck | 45 | 1 |
| 2022.08.04 | Shandong | Duck | Cherry Valley Duck | 45 | 1 |
| 2022.08.04 | Shandong | Duck | Cherry Valley Duck | 45 | 1 |
| 2022.08.04 | Shandong | Duck | Cherry Valley Duck | 45 | 1 |
| 2022.08.04 | Shandong | Duck | Cherry Valley Duck | 45 | 1 |
| 2022.08.04 | Shandong | Duck | Cherry Valley Duck | 45 | 1 |
| 2022.08.04 | Shandong | Duck | Cherry Valley Duck | 45 | 1 |
| 2022.08.04 | Shandong | Duck | Cherry Valley Duck | 45 | 1 |
| 2022.08.04 | Shandong | Duck | Cherry Valley Duck | 45 | 1 |
| 2022.08.04 | Shandong | Duck | Cherry Valley Duck | 45 | 1 |
| 2022.08.04 | Shandong | Duck | Cherry Valley Duck | 45 | 1 |
| 2022.08.04 | Shandong | Duck | Cherry Valley Duck | 45 | 1 |
| 2022.08.04 | Shandong | Duck | Cherry Valley Duck | 45 | 1 |
| 2022.08.04 | Shandong | Duck | Cherry Valley Duck | 45 | 1 |
| 2022.08.04 | Shandong | Duck | Cherry Valley Duck | 45 | 1 |
| 2022.08.04 | Shandong | Duck | Cherry Valley Duck | 45 | 1 |
| 2022.08.04 | Guangdong | Duck | White Duck | 48 | 1 |
| 2022.08.04 | Guangdong | Duck | White Duck | 48 | 1 |
| 2022.08.04 | Guangdong | Duck | White Duck | 48 | 1 |
| 2022.08.04 | Guangdong | Duck | White Duck | 48 | 1 |
| 2022.08.04 | Guangdong | Duck | White Duck | 48 | 1 |
| 2022.08.04 | Guangdong | Duck | White Duck | 48 | 1 |
| 2022.08.05 | Shandong | environment | / | / | 1 |
| 2022.08.05 | Shandong | Duck | Cherry Valley Duck | 37 | 1 |
| 2022.08.05 | Shandong | Duck | Cherry Valley Duck | 37 | 1 |
| 2022.08.05 | Guangdong | Duck | White Duck | 32 | 1 |
| 2022.08.05 | Guangdong | Duck | White Duck | 32 | 1 |
| 2022.08.05 | Guangdong | Duck | White Duck | 32 | 1 |
| 2022.08.05 | Guangdong | Duck | / | 75 | 1 |
| 2022.08.05 | Guangdong | Duck | / | 75 | 1 |
| 2022.08.05 | Guangdong | Duck | / | 200 | 1 |
| 2022.08.08 | Shandong | Duck | Cherry Valley Duck | 20 | 1 |
| 2022.08.08 | Shandong | Duck | Cherry Valley Duck | 20 | 1 |
| 2022.08.08 | Shandong | Duck | Cherry Valley Duck | 20 | 1 |
| 2022.08.08 | Guangdong | Duck | Cherry Valley Duck | 3 | 1 |
| 2022.08.08 | Guangdong | Duck | Cherry Valley Duck | 3 | 1 |
| 2022.08.08 | Guangdong | Duck | Cherry Valley Duck | 3 | 1 |
| 2022.08.08 | Guangdong | Duck | Cherry Valley Duck | 3 | 1 |
| 2022.08.08 | Guangdong | Duck | Cherry Valley Duck | 3 | 1 |
| 2022.08.08 | Guangdong | Duck | Cherry Valley Duck | 3 | 1 |
| 2022.08.08 | Guangdong | Duck | Cherry Valley Duck | 3 | 1 |
| 2022.08.08 | Guangdong | Duck | Cherry Valley Duck | 3 | 1 |
| 2022.08.08 | Guangdong | Duck | Cherry Valley Duck | 3 | 1 |
| 2022.08.08 | Guangdong | Duck | Cherry Valley Duck | 3 | 1 |
| 2022.08.08 | Guangdong | Duck | Cherry Valley Duck | 3 | 1 |
| 2022.08.08 | Guangdong | Duck | Cherry Valley Duck | 3 | 1 |
| 2022.08.08 | Guangdong | Duck | Cherry Valley Duck | 3 | 1 |
| 2022.08.08 | Guangdong | Duck | Cherry Valley Duck | 3 | 1 |
| 2022.08.08 | Guangdong | Duck | Cherry Valley Duck | 3 | 1 |
| 2022.08.09 | Heilongjiang | Duck | / | 20 | 1 |
| 2022.08.09 | Heilongjiang | Duck | / | 20 | 1 |
| 2022.08.09 | Guangxi | Duck | / | 27 | 1 |
| 2022.08.09 | Anhui | Duck | / | / | 1 |
| 2022.08.09 | Anhui | Duck | / | / | 1 |
| 2022.08.09 | Anhui | Duck | / | / | 1 |
| 2022.08.09 | Anhui | Duck | / | / | 1 |
| 2022.08.09 | Anhui | Duck | / | / | 1 |
| 2022.08.10 | Henan | Duck | White Duck | 3 | 1 |
| 2022.08.10 | Henan | Duck | White Duck | 3 | 1 |
| 2022.08.10 | Henan | Duck | White Duck | 3 | 1 |
| 2022.08.10 | Henan | Duck | White Duck | 3 | 1 |
| 2022.08.10 | Henan | Duck | White Duck | 3 | 1 |
| 2022.08.10 | Henan | Duck | White Duck | 3 | 1 |
| 2022.08.10 | Henan | Duck | White Duck | 3 | 1 |
| 2022.08.10 | Henan | Duck | White Duck | 3 | 1 |
| 2022.08.10 | Shandong | Duck | Cherry Valley Duck | 31 | 1 |
| 2022.08.10 | Shandong | Duck | Cherry Valley Duck | 31 | 1 |
| 2022.08.10 | Shandong | Duck | Cherry Valley Duck | 31 | 1 |
| 2022.08.10 | Henan | Duck | / | 22 | 1 |
| 2022.08.10 | Henan | Duck | / | 22 | 1 |
| 2022.08.10 | Henan | Duck | / | 22 | 1 |
| 2022.08.10 | Henan | Duck | / | 22 | 1 |
| 2022.08.10 | Henan | Duck | / | 49 | 1 |
| 2022.08.10 | Henan | Duck | / | 49 | 1 |
| 2022.08.10 | Henan | Duck | / | 49 | 1 |
| 2022.08.10 | Henan | Duck | / | 49 | 1 |
| 2022.08.10 | Henan | Duck | / | 49 | 1 |
| 2022.08.10 | Henan | Duck | / | 49 | 1 |
| 2022.08.10 | Guangdong | Duck | Muscovy Duck | 25 | 1 |
| 2022.08.10 | Guangdong | Duck | Shelduck | 32 | 1 |
| 2022.08.10 | Guangdong | Duck | Shelduck | 32 | 1 |
| 2022.08.10 | Guangdong | Duck | Shelduck | 32 | 1 |
| 2022.08.10 | Anhui | Duck | Cherry Valley Duck | 19 | 1 |
| 2022.08.10 | Guangxi | Duck | / | 47 | 1 |
| 2022.08.10 | Guangxi | Duck | / | 47 | 1 |
| 2022.08.10 | Anhui | Duck | Muscovy Duck | 70 | 1 |
| 2022.08.11 | Shandong | Duck | / | 28 | 1 |
| 2022.08.11 | Guangxi | Duck | Shelduck | 24 | 1 |
| 2022.08.11 | Jiangxi | Duck | Muscovy Duck | 15 | 1 |
| 2022.08.11 | Guangdong | Duck | White Duck | 19 | 1 |
| 2022.08.11 | Guangdong | Duck | White Duck | 19 | 1 |
| 2022.08.11 | Guangdong | Duck | White Duck | 19 | 1 |
| 2022.08.11 | Guangdong | Duck | White Duck | 19 | 1 |
| 2022.08.11 | Guangdong | Duck | White Duck | 19 | 1 |
| 2022.08.11 | Guangxi | Duck | Muscovy Duck | 30 | 1 |
| 2022.08.11 | Guangxi | Duck | Muscovy Duck | 30 | 1 |
| 2022.08.11 | Shandong | Duck | Cherry Valley Duck | 22 | 1 |
| 2022.08.11 | Shandong | Duck | Cherry Valley Duck | 22 | 1 |
| 2022.08.11 | Shandong | Duck | Cherry Valley Duck | 22 | 1 |
| 2022.08.15 | Shandong | Duck | Cherry Valley Duck | 3 | 1 |
| 2022.08.15 | Shandong | Duck | Cherry Valley Duck | 3 | 1 |
| 2022.08.15 | Shandong | Duck | Cherry Valley Duck | 3 | 1 |
| 2022.08.15 | Shandong | Duck | Cherry Valley Duck | 3 | 1 |
| 2022.08.15 | Shandong | Duck | Cherry Valley Duck | 3 | 1 |
| 2022.08.15 | Shandong | Duck | Cherry Valley Duck | 3 | 1 |
| 2022.08.15 | Shandong | Duck | Cherry Valley Duck | 3 | 1 |
| 2022.08.15 | Shandong | Duck | Cherry Valley Duck | 3 | 1 |
| 2022.08.15 | Shandong | Duck | Cherry Valley Duck | 3 | 1 |
| 2022.08.15 | Shandong | Duck | Cherry Valley Duck | 3 | 1 |
| 2022.08.15 | Shandong | Duck | Cherry Valley Duck | 3 | 1 |
| 2022.08.15 | Shandong | Duck | Cherry Valley Duck | 3 | 1 |
| 2022.08.15 | Shandong | Duck | Cherry Valley Duck | 3 | 1 |
| 2022.08.15 | Shandong | Duck | Cherry Valley Duck | 3 | 1 |
| 2022.08.15 | Shandong | Duck | Cherry Valley Duck | 3 | 1 |
| 2022.08.15 | Shandong | Duck | Cherry Valley Duck | 3 | 1 |
| 2022.08.15 | Shandong | Duck | Cherry Valley Duck | 3 | 1 |
| 2022.08.15 | Shandong | Duck | Cherry Valley Duck | 3 | 1 |
| 2022.08.15 | Shandong | Duck | Cherry Valley Duck | 3 | 1 |
| 2022.08.15 | Shandong | Duck | Cherry Valley Duck | 3 | 1 |
| 2022.08.15 | Shandong | Duck | Cherry Valley Duck | 3 | 1 |
| 2022.08.15 | Shandong | Duck | Cherry Valley Duck | 3 | 1 |
| 2022.08.15 | Shandong | Duck | Cherry Valley Duck | 3 | 1 |
| 2022.08.15 | Shandong | Duck | Cherry Valley Duck | 3 | 1 |
| 2022.08.15 | Shandong | Duck | Cherry Valley Duck | 3 | 1 |
| 2022.08.15 | Shandong | Duck | Cherry Valley Duck | 3 | 1 |
| 2022.08.15 | Shandong | Duck | Cherry Valley Duck | 3 | 1 |
| 2022.08.15 | Shandong | Duck | Cherry Valley Duck | 3 | 1 |
| 2022.08.15 | Shandong | Duck | Cherry Valley Duck | 3 | 1 |
| 2022.08.15 | Shandong | Duck | Cherry Valley Duck | 3 | 1 |
| 2022.08.15 | Shandong | Duck | Cherry Valley Duck | 3 | 1 |
| 2022.08.15 | Shandong | Duck | Cherry Valley Duck | 3 | 1 |
| 2022.08.15 | Shandong | Duck | Cherry Valley Duck | 3 | 1 |
| 2022.08.15 | Shandong | Duck | Cherry Valley Duck | 3 | 1 |
| 2022.08.15 | Shandong | Duck | Cherry Valley Duck | 3 | 1 |
| 2022.08.15 | Shandong | Duck | Cherry Valley Duck | 3 | 1 |
| 2022.08.15 | Shandong | Duck | Cherry Valley Duck | 3 | 1 |
| 2022.08.15 | Shandong | Duck | Cherry Valley Duck | 3 | 1 |
| 2022.08.15 | Shandong | Duck | Cherry Valley Duck | 3 | 1 |
| 2022.08.15 | Shandong | Duck | Cherry Valley Duck | 3 | 1 |
| 2022.08.15 | Shandong | Duck | Cherry Valley Duck | 28 | 1 |
| 2022.08.15 | Shandong | Duck | Cherry Valley Duck | 28 | 1 |
| 2022.08.15 | Shandong | Duck | Cherry Valley Duck | 28 | 1 |
| 2022.08.15 | Shandong | Duck | Cherry Valley Duck | 28 | 1 |
| 2022.08.15 | Shandong | Duck | Cherry Valley Duck | 28 | 1 |
| 2022.08.15 | Shandong | Duck | Cherry Valley Duck | 28 | 1 |
| 2022.08.15 | Shandong | Duck | Cherry Valley Duck | 28 | 1 |
| 2022.08.15 | Shandong | Duck | Cherry Valley Duck | 28 | 1 |
| 2022.08.15 | Shandong | Duck | Cherry Valley Duck | 28 | 1 |
| 2022.08.15 | Shandong | Duck | Cherry Valley Duck | 28 | 1 |
| 2022.08.15 | Shandong | Duck | Cherry Valley Duck | 28 | 1 |
| 2022.08.15 | Shandong | Duck | Cherry Valley Duck | 28 | 1 |
| 2022.08.15 | Shandong | Duck | Cherry Valley Duck | 28 | 1 |
| 2022.08.15 | Guangdong | Duck | White Duck | 20 | 1 |
| 2022.08.15 | Guangdong | Duck | White Duck | 20 | 1 |
| 2022.08.15 | Anhui | Duck | Cherry Valley Duck | 3 | 1 |
| 2022.08.15 | Anhui | Duck | Cherry Valley Duck | 3 | 1 |
| 2022.08.15 | Jiangsu | Duck | / | / | 1 |
| 2022.08.15 | Jiangsu | Duck | / | / | 1 |
| 2022.08.15 | Jiangsu | Duck | / | / | 1 |
| 2022.08.15 | Jiangsu | Duck | / | / | 1 |
| 2022.08.15 | Jiangsu | Duck | / | / | 1 |
| 2022.08.17 | Anhui | Duck | Muscovy Duck | 260 | 1 |
| 2022.08.17 | Guangdong | Duck | Cherry Valley Duck | 32 | 1 |
| 2022.08.17 | Guangdong | Duck | Cherry Valley Duck | 32 | 1 |
| 2022.08.17 | Guangdong | Duck | Cherry Valley Duck | 32 | 1 |
| 2022.08.17 | Guangdong | Duck | Mule Duck | 39 | 1 |
| 2022.08.17 | Guangdong | Duck | Mule Duck | 39 | 1 |
| 2022.08.17 | Guangdong | Duck | Mule Duck | 39 | 1 |
| 2022.08.17 | Guangdong | Duck | Mule Duck | 39 | 1 |
| 2022.08.18 | Shandong | Duck | / | 22 | 1 |
| 2022.08.18 | Heilongjiang | Duck | Native Duck | 62 | 1 |
| 2022.08.18 | Heilongjiang | Duck | Native Duck | 62 | 1 |
| 2022.08.18 | Heilongjiang | Duck | Native Duck | 62 | 1 |
| 2022.08.18 | Heilongjiang | Duck | Native Duck | 62 | 1 |
| 2022.08.18 | Heilongjiang | Duck | Native Duck | 62 | 1 |
| 2022.08.19 | Henan | Duck | White Duck | 5 | 1 |
| 2022.08.19 | Henan | Duck | White Duck | 5 | 1 |
| 2022.08.19 | Henan | Duck | White Duck | 5 | 1 |
| 2022.08.19 | Henan | Duck | White Duck | 5 | 1 |
| 2022.08.19 | Shandong | Duck | Cherry Valley Duck | 36 | 1 |
| 2022.08.19 | Shandong | Duck | Cherry Valley Duck | 36 | 1 |
| 2022.08.19 | Shandong | Duck | Cherry Valley Duck | 36 | 1 |
| 2022.08.19 | Shandong | Duck | Cherry Valley Duck | / | 1 |
| 2022.08.19 | Shandong | Duck | Cherry Valley Duck | / | 1 |
| 2022.08.19 | Shandong | Duck | Cherry Valley Duck | / | 1 |
| 2022.08.19 | Shandong | Duck | Cherry Valley Duck | / | 1 |
| 2022.08.19 | Shandong | Duck | Cherry Valley Duck | / | 1 |
| 2022.08.19 | Shandong | Duck | Cherry Valley Duck | / | 1 |
| 2022.08.19 | Shandong | Duck | Cherry Valley Duck | / | 1 |
| 2022.08.19 | Shandong | Duck | Cherry Valley Duck | / | 1 |
| 2022.08.19 | Shandong | Duck | Cherry Valley Duck | / | 1 |
| 2022.08.19 | Shandong | Duck | Cherry Valley Duck | / | 1 |
| 2022.08.19 | Shandong | Duck | Cherry Valley Duck | / | 1 |
| 2022.08.19 | Shandong | Duck | Cherry Valley Duck | / | 1 |
| 2022.08.19 | Shandong | Duck | Cherry Valley Duck | / | 1 |
| 2022.08.19 | Shandong | Duck | Cherry Valley Duck | / | 1 |
| 2022.08.19 | Shandong | Duck | Cherry Valley Duck | / | 1 |
| 2022.08.19 | Shandong | Duck | Cherry Valley Duck | / | 1 |
| 2022.08.19 | Shandong | Duck | Cherry Valley Duck | / | 1 |
| 2022.08.19 | Shandong | Duck | Cherry Valley Duck | / | 1 |
| 2022.08.19 | Shandong | Duck | Cherry Valley Duck | / | 1 |
| 2022.08.19 | Shandong | Duck | Cherry Valley Duck | / | 1 |
| 2022.08.19 | Shandong | Duck | Cherry Valley Duck | / | 1 |
| 2022.08.19 | Shandong | Duck | Cherry Valley Duck | / | 1 |
| 2022.08.19 | Shandong | Duck | Cherry Valley Duck | / | 1 |
| 2022.08.19 | Shandong | Duck | Cherry Valley Duck | / | 1 |
| 2022.08.19 | Shandong | Duck | Cherry Valley Duck | / | 1 |
| 2022.08.19 | Shandong | Duck | Cherry Valley Duck | / | 1 |
| 2022.08.19 | Shandong | Duck | Cherry Valley Duck | / | 1 |
| 2022.08.19 | Shandong | Duck | Cherry Valley Duck | / | 1 |
| 2022.08.19 | Shandong | Duck | Cherry Valley Duck | / | 1 |
| 2022.08.19 | Shandong | Duck | Cherry Valley Duck | / | 1 |
| 2022.08.19 | Shandong | Duck | Cherry Valley Duck | / | 1 |
| 2022.08.19 | Shandong | Duck | Cherry Valley Duck | / | 1 |
| 2022.08.19 | Shandong | Duck | Cherry Valley Duck | / | 1 |
| 2022.08.19 | Shandong | Duck | Cherry Valley Duck | / | 1 |
| 2022.08.19 | Shandong | Duck | Cherry Valley Duck | / | 1 |
| 2022.08.19 | Shandong | Duck | Cherry Valley Duck | / | 1 |
| 2022.08.19 | Shandong | Duck | Cherry Valley Duck | / | 1 |
| 2022.08.19 | Shandong | Duck | Cherry Valley Duck | / | 1 |
| 2022.08.19 | Shandong | Duck | Cherry Valley Duck | / | 1 |
| 2022.08.19 | Shandong | Duck | Cherry Valley Duck | / | 1 |
| 2022.08.19 | Shandong | Duck | Cherry Valley Duck | / | 1 |
| 2022.08.19 | Shandong | Duck | Cherry Valley Duck | / | 1 |
| 2022.08.19 | Shandong | Duck | Cherry Valley Duck | / | 1 |
| 2022.08.19 | Shandong | Duck | Cherry Valley Duck | / | 1 |
| 2022.08.19 | Shandong | Duck | Cherry Valley Duck | / | 1 |
| 2022.08.19 | Shandong | Duck | Cherry Valley Duck | / | 1 |
| 2022.08.19 | Shandong | Duck | Cherry Valley Duck | / | 1 |
| 2022.08.19 | Shandong | Duck | Cherry Valley Duck | / | 1 |
| 2022.08.19 | Shandong | Duck | Cherry Valley Duck | / | 1 |
| 2022.08.19 | Shandong | Duck | Cherry Valley Duck | / | 1 |
| 2022.08.19 | Shandong | Duck | Cherry Valley Duck | / | 1 |
| 2022.08.19 | Shandong | Duck | Cherry Valley Duck | / | 1 |
| 2022.08.19 | Shandong | Duck | Cherry Valley Duck | / | 1 |
| 2022.08.19 | Shandong | Duck | Cherry Valley Duck | / | 1 |
| 2022.08.19 | Shandong | Duck | Cherry Valley Duck | / | 1 |
| 2022.08.19 | Shandong | Duck | Cherry Valley Duck | / | 1 |
| 2022.08.19 | Shandong | Duck | Cherry Valley Duck | / | 1 |
| 2022.08.19 | Shandong | Duck | Cherry Valley Duck | / | 1 |
| 2022.08.19 | Shandong | Duck | Cherry Valley Duck | / | 1 |
| 2022.08.19 | Shandong | Duck | Cherry Valley Duck | / | 1 |
| 2022.08.19 | Shandong | Duck | Cherry Valley Duck | / | 1 |
| 2022.08.19 | Shandong | Duck | Cherry Valley Duck | / | 1 |
| 2022.08.19 | Shandong | Duck | Cherry Valley Duck | / | 1 |
| 2022.08.19 | Shandong | Duck | Cherry Valley Duck | / | 1 |
| 2022.08.19 | Guangdong | Duck | / | / | 1 |
| 2022.08.19 | Guangdong | Duck | / | / | 1 |
| 2022.08.19 | Guangdong | Duck | / | / | 1 |
| 2022.08.19 | Guangdong | Duck | / | / | 1 |
| 2022.08.19 | Guangdong | Duck | / | / | 1 |
| 2022.08.22 | Shandong | Duck | Cherry Valley Duck | 31 | 1 |
| 2022.08.22 | Shandong | Duck | Cherry Valley Duck | 31 | 1 |
| 2022.08.22 | Shandong | Duck | Cherry Valley Duck | 31 | 1 |
| 2022.08.22 | Henan | Duck | White Duck | 5 | 1 |
| 2022.08.22 | Jiangxi | Duck | Muscovy Duck | 29 | 1 |
| 2022.08.22 | Jiangxi | Duck | Muscovy Duck | 17 | 1 |
| 2022.08.22 | Jiangxi | Duck | Muscovy Duck | 20 | 1 |
| 2022.08.22 | Guangdong | Duck | / | / | 1 |
| 2022.08.22 | Guangdong | Duck | / | / | 1 |
| 2022.08.22 | Guangdong | Duck | White Duck | 20 | 1 |
| 2022.08.22 | Guangdong | Duck | Muscovy Duck | 10 | 1 |
| 2022.08.22 | Guangdong | Duck | Cherry Valley Duck | 35 | 1 |
| 2022.08.22 | Guangdong | Duck | Cherry Valley Duck | 35 | 1 |
| 2022.08.22 | Guangdong | Duck | Cherry Valley Duck | 35 | 1 |
| 2022.08.22 | Guangdong | Duck | Cherry Valley Duck | 35 | 1 |
| 2022.08.22 | Guangxi | Duck | White Duck | 38 | 1 |
| 2022.08.22 | Guangxi | Duck | White Duck | 38 | 1 |
| 2022.08.23 | Shandong | Duck | Cherry Valley Duck | 3 | 1 |
| 2022.08.23 | Shandong | Duck | Cherry Valley Duck | 3 | 1 |
| 2022.08.23 | Shandong | Duck | Cherry Valley Duck | 3 | 1 |
| 2022.08.23 | Shandong | Duck | Cherry Valley Duck | 3 | 1 |
| 2022.08.23 | Shandong | Duck | Cherry Valley Duck | 3 | 1 |
| 2022.08.23 | Shandong | Duck | Cherry Valley Duck | 3 | 1 |
| 2022.08.23 | Shandong | Duck | Cherry Valley Duck | 3 | 1 |
| 2022.08.23 | Shandong | Duck | Cherry Valley Duck | 3 | 1 |
| 2022.08.23 | Shandong | Duck | Cherry Valley Duck | 3 | 1 |
| 2022.08.23 | Shandong | Duck | Cherry Valley Duck | 3 | 1 |
| 2022.08.23 | Shandong | Duck | Cherry Valley Duck | 3 | 1 |
| 2022.08.23 | Shandong | Duck | Cherry Valley Duck | 3 | 1 |
| 2022.08.23 | Shandong | Duck | Cherry Valley Duck | 3 | 1 |
| 2022.08.23 | Shandong | Duck | Cherry Valley Duck | 3 | 1 |
| 2022.08.23 | Shandong | Duck | Cherry Valley Duck | 3 | 1 |
| 2022.08.23 | Shandong | Duck | Cherry Valley Duck | 3 | 1 |
| 2022.08.23 | Shandong | Duck | Cherry Valley Duck | 32 | 1 |
| 2022.08.23 | Shandong | Duck | Cherry Valley Duck | 32 | 1 |
| 2022.08.23 | Shandong | Duck | Cherry Valley Duck | 32 | 1 |
| 2022.08.23 | Shandong | Duck | Cherry Valley Duck | 32 | 1 |
| 2022.08.23 | Shandong | Duck | Cherry Valley Duck | 32 | 1 |
| 2022.08.23 | Shandong | Duck | Cherry Valley Duck | 32 | 1 |
| 2022.08.23 | Shandong | Duck | Cherry Valley Duck | 32 | 1 |
| 2022.08.23 | Shandong | Duck | Cherry Valley Duck | 32 | 1 |
| 2022.08.24 | Liaoning | Duck | / | 364 | 1 |
| 2022.08.24 | Liaoning | Duck | / | 406 | 1 |
| 2022.08.24 | Liaoning | Duck | / | 364 | 1 |
| 2022.08.24 | Nei Mongol | Duck | / | 1 | 1 |
| 2022.08.24 | Nei Mongol | Duck | / | 1 | 1 |
| 2022.08.24 | Nei Mongol | Duck | / | 1 | 1 |
| 2022.08.24 | Nei Mongol | Duck | / | 1 | 1 |
| 2022.08.24 | Nei Mongol | Duck | / | 1 | 1 |
| 2022.08.24 | Nei Mongol | Duck | / | 1 | 1 |
| 2022.08.24 | Nei Mongol | Duck | / | 1 | 1 |
| 2022.08.24 | Nei Mongol | Duck | / | 1 | 1 |
| 2022.08.24 | Nei Mongol | Duck | / | 1 | 1 |
| 2022.08.24 | Nei Mongol | Duck | / | 1 | 1 |
| 2022.08.24 | Nei Mongol | Duck | / | 1 | 1 |
| 2022.08.24 | Nei Mongol | Duck | / | 1 | 1 |
| 2022.08.25 | Shandong | Duck | Cherry Valley Duck | 3 | 1 |
| 2022.08.25 | Shandong | Duck | Cherry Valley Duck | 3 | 1 |
| 2022.08.25 | Shandong | Duck | Cherry Valley Duck | 3 | 1 |
| 2022.08.25 | Shandong | Duck | Cherry Valley Duck | 3 | 1 |
| 2022.08.25 | Shandong | Duck | Cherry Valley Duck | 3 | 1 |
| 2022.08.25 | Shandong | Duck | Cherry Valley Duck | 3 | 1 |
| 2022.08.25 | Shandong | Duck | Cherry Valley Duck | 3 | 1 |
| 2022.08.25 | Shandong | Duck | Cherry Valley Duck | 3 | 1 |
| 2022.08.25 | Shandong | Duck | Cherry Valley Duck | 3 | 1 |
| 2022.08.25 | Shandong | Duck | Cherry Valley Duck | 3 | 1 |
| 2022.08.25 | Shandong | Duck | Cherry Valley Duck | 3 | 1 |
| 2022.08.25 | Shandong | Duck | Cherry Valley Duck | 3 | 1 |
| 2022.08.25 | Shandong | Duck | Cherry Valley Duck | 3 | 1 |
| 2022.08.25 | Shandong | Duck | Cherry Valley Duck | 3 | 1 |
| 2022.08.25 | Shandong | Duck | Cherry Valley Duck | 3 | 1 |
| 2022.08.25 | Shandong | Duck | Cherry Valley Duck | 3 | 1 |
| 2022.08.25 | Shandong | Duck | Cherry Valley Duck | 3 | 1 |
| 2022.08.25 | Shandong | Duck | Cherry Valley Duck | 3 | 1 |
| 2022.08.25 | Shandong | Duck | Cherry Valley Duck | 3 | 1 |
| 2022.08.25 | Shandong | Duck | Cherry Valley Duck | 3 | 1 |
| 2022.08.25 | Shandong | Duck | Cherry Valley Duck | 3 | 1 |
| 2022.08.25 | Shandong | Duck | Cherry Valley Duck | 3 | 1 |
| 2022.08.25 | Shandong | Duck | Cherry Valley Duck | 3 | 1 |
| 2022.08.25 | Shandong | Duck | Cherry Valley Duck | 3 | 1 |
| 2022.08.25 | Shandong | Duck | Cherry Valley Duck | 30 | 1 |
| 2022.08.25 | Shandong | Duck | Cherry Valley Duck | 30 | 1 |
| 2022.08.25 | Shandong | Duck | Cherry Valley Duck | 30 | 1 |
| 2022.08.25 | Shandong | Duck | Cherry Valley Duck | 13 | 1 |
| 2022.08.25 | Guangdong | Duck | Muscovy Duck | 29 | 1 |
| 2022.08.25 | Guangdong | Duck | Shelduck | 29 | 1 |
| 2022.08.25 | Guangdong | Duck | Muscovy Duck | 29 | 1 |
| 2022.08.25 | Guangdong | Duck | White Duck | 17 | 1 |
| 2022.08.25 | Guangdong | Duck | White Duck | 17 | 1 |
| 2022.08.25 | Guangdong | Duck | White Duck | 17 | 1 |
| 2022.08.25 | Guangdong | Duck | White Duck | 17 | 1 |
| 2022.08.25 | Guangdong | Duck | White Duck | 17 | 1 |
| 2022.08.25 | Guangdong | Duck | White Duck | 17 | 1 |
| 2022.08.25 | Guangdong | Duck | White Duck | 17 | 1 |
| 2022.08.25 | Guangdong | Duck | White Duck | 17 | 1 |
| 2022.08.25 | Guangdong | Duck | White Duck | 17 | 1 |
| 2022.08.25 | Guangdong | Duck | White Duck | 17 | 1 |
| 2022.08.25 | Guangdong | Duck | White Duck | 17 | 1 |
| 2022.08.25 | Guangdong | Duck | White Duck | 17 | 1 |
| 2022.08.25 | Guangdong | Duck | White Duck | 17 | 1 |
| 2022.08.25 | Guangdong | Duck | White Duck | 17 | 1 |
| 2022.08.26 | Guangdong | Duck | White Duck | 20 | 1 |
| 2022.08.26 | Guangdong | Duck | White Duck | 20 | 1 |
| 2022.08.29 | Nei Mongol | Duck | Cherry Valley Duck | 19 | 1 |
| 2022.08.29 | Nei Mongol | Duck | Cherry Valley Duck | 19 | 1 |
| 2022.08.29 | Shandong | Duck | / | 16 | 1 |
| 2022.08.29 | Shandong | Duck | / | 12 | 1 |
| 2022.08.29 | Shandong | Duck | / | 12 | 1 |
| 2022.08.29 | Shandong | Duck | / | 12 | 1 |
| 2022.08.29 | Shandong | Duck | / | 12 | 1 |
| 2022.08.29 | Shandong | Duck | / | 12 | 1 |
| 2022.08.29 | Shandong | Duck | / | 12 | 1 |
| 2022.08.29 | Shandong | Duck | / | 12 | 1 |
| 2022.08.29 | Shandong | Duck | / | 12 | 1 |
| 2022.08.29 | Shandong | Duck | / | 12 | 1 |
| 2022.08.29 | Shandong | Duck | / | 12 | 1 |
| 2022.08.29 | Shandong | Duck | / | 12 | 1 |
| 2022.08.29 | Shandong | Duck | / | 12 | 1 |
| 2022.08.29 | Shandong | Duck | / | 12 | 1 |
| 2022.08.29 | Shandong | Duck | / | 12 | 1 |
| 2022.08.29 | Shandong | Duck | / | 12 | 1 |
| 2022.08.29 | Shandong | Duck | / | 12 | 1 |
| 2022.08.29 | Anhui | Duck | Cherry Valley Duck | 27 | 1 |
| 2022.08.29 | Guangdong | Duck | Shelduck | 21 | 1 |
| 2022.08.29 | Guangdong | Duck | Shelduck | 21 | 1 |
| 2022.08.29 | Guangdong | Duck | Shelduck | 21 | 1 |
| 2022.08.29 | Guangdong | Duck | Shelduck | 21 | 1 |
| 2022.08.29 | Guangdong | Duck | Shelduck | 21 | 1 |
| 2022.08.29 | Guangdong | Duck | Shelduck | 21 | 1 |
| 2022.08.29 | Guangdong | Duck | Shelduck | 21 | 1 |
| 2022.08.29 | Guangdong | Duck | Muscovy Duck | 55 | 1 |
| 2022.08.29 | Guangdong | Duck | Muscovy Duck | 55 | 1 |
| 2022.08.29 | Guangdong | Duck | / | 15 | 1 |
| 2022.08.29 | Guangdong | Duck | / | 15 | 1 |
| 2022.08.29 | Guangdong | Duck | / | 15 | 1 |
| 2022.08.29 | Guangdong | Duck | / | 15 | 1 |
| 2022.08.29 | Guangdong | Duck | / | 15 | 1 |
| 2022.08.29 | Guangdong | Duck | / | 15 | 1 |
| 2022.08.29 | Guangdong | Duck | / | 15 | 1 |
| 2022.08.29 | Guangdong | Duck | / | 15 | 1 |
| 2022.08.30 | Hebei | Duck | / | 20 | 1 |
| 2022.08.30 | Hebei | Duck | / | 20 | 1 |
| 2022.08.30 | Hebei | Duck | / | 20 | 1 |
| 2022.08.30 | Hebei | Duck | / | 20 | 1 |
| 2022.08.30 | Shandong | Duck | Cherry Valley Duck | 252 | 1 |
| 2022.08.30 | Shandong | Duck | Cherry Valley Duck | 252 | 1 |
| 2022.08.30 | Shandong | Duck | Cherry Valley Duck | 252 | 1 |
| 2022.08.30 | Shandong | Duck | Cherry Valley Duck | 252 | 1 |
| 2022.08.30 | Shandong | Duck | Cherry Valley Duck | 252 | 1 |
| 2022.08.30 | Guangdong | Duck | White Duck | 33 | 1 |
| 2022.08.30 | Jiangxi | Duck | Muscovy Duck | 9 | 1 |
| 2022.08.30 | Jiangxi | Duck | Muscovy Duck | 9 | 1 |
| 2022.08.31 | Nei Mongol | Duck | Cherry Valley Duck | 3 | 1 |
| 2022.09.01 | Jiangsu | Duck | White Duck | 24 | 1 |
| 2022.09.01 | Jiangsu | Duck | White Duck | 24 | 1 |
| 2022.09.01 | Jiangsu | Duck | White Duck | 24 | 1 |
| 2022.09.01 | Jiangsu | Duck | White Duck | 24 | 1 |
| 2022.09.01 | Jiangsu | Duck | White Duck | 21 | 1 |
| 2022.09.01 | Jiangsu | Duck | White Duck | 25 | 1 |
| 2022.09.05 | Shandong | Duck | Cherry Valley Duck | 30 | 1 |
| 2022.09.05 | Shandong | Duck | Cherry Valley Duck | 30 | 1 |
| 2022.09.05 | Shandong | Duck | Cherry Valley Duck | 30 | 1 |
| 2022.09.05 | Shandong | Duck | Cherry Valley Duck | 30 | 1 |
| 2022.09.05 | Shandong | Duck | Cherry Valley Duck | 30 | 1 |
| 2022.09.05 | Shandong | Duck | Cherry Valley Duck | 30 | 1 |
| 2022.09.05 | Shandong | Duck | Cherry Valley Duck | 30 | 1 |
| 2022.09.05 | Shandong | Duck | Cherry Valley Duck | 30 | 1 |
| 2022.09.05 | Shandong | Duck | Cherry Valley Duck | 30 | 1 |
| 2022.09.05 | Shandong | Duck | Cherry Valley Duck | 30 | 1 |
| 2022.09.05 | Jiangxi | Duck | Muscovy Duck | 13 | 1 |
| 2022.09.05 | Jiangxi | Duck | Muscovy Duck | 13 | 1 |
| 2022.09.05 | Jiangxi | Duck | Muscovy Duck | 13 | 1 |
| 2022.09.05 | Shandong | Duck | / | 469 | 1 |
| 2022.09.05 | Shandong | Duck | / | 469 | 1 |
| 2022.09.05 | Shandong | Duck | / | 469 | 1 |
| 2022.09.05 | Shandong | Duck | / | 469 | 1 |
| 2022.09.05 | Shandong | Duck | / | 469 | 1 |
| 2022.09.05 | Shandong | Duck | / | 469 | 1 |
| 2022.09.05 | Shandong | Duck | / | 469 | 1 |
| 2022.09.05 | Shandong | Duck | / | 469 | 1 |
| 2022.09.06 | Shandong | Duck | White Duck | 22 | 1 |
| 2022.09.06 | Shandong | Duck | White Duck | 22 | 1 |
| 2022.09.06 | Shandong | Duck | White Duck | 22 | 1 |
| 2022.09.07 | Zhejiang | Duck | Cherry Valley Duck | 272 | 1 |
| 2022.09.07 | Zhejiang | Duck | Cherry Valley Duck | 272 | 1 |
| 2022.09.07 | Zhejiang | Duck | Cherry Valley Duck | 272 | 1 |
| 2022.09.07 | Zhejiang | Duck | Cherry Valley Duck | 272 | 1 |
| 2022.09.07 | Zhejiang | Duck | Cherry Valley Duck | 272 | 1 |
| 2022.09.07 | Zhejiang | Duck | Cherry Valley Duck | 272 | 1 |
| 2022.09.07 | Zhejiang | Duck | Cherry Valley Duck | 272 | 1 |
| 2022.09.07 | Zhejiang | Duck | Cherry Valley Duck | 272 | 1 |
| 2022.09.07 | Zhejiang | Duck | Cherry Valley Duck | 272 | 1 |
| 2022.09.07 | Zhejiang | Duck | Cherry Valley Duck | 272 | 1 |
| 2022.09.07 | Zhejiang | Duck | Cherry Valley Duck | 272 | 1 |
| 2022.09.07 | Zhejiang | Duck | Cherry Valley Duck | 272 | 1 |
| 2022.09.07 | Zhejiang | Duck | Cherry Valley Duck | 272 | 1 |
| 2022.09.07 | Zhejiang | Duck | Cherry Valley Duck | 272 | 1 |
| 2022.09.07 | Zhejiang | Duck | Cherry Valley Duck | 272 | 1 |
| 2022.09.07 | Zhejiang | Duck | Cherry Valley Duck | 272 | 1 |
| 2022.09.07 | Zhejiang | Duck | Cherry Valley Duck | 272 | 1 |
| 2022.09.07 | Zhejiang | Duck | Cherry Valley Duck | 272 | 1 |
| 2022.09.07 | Zhejiang | Duck | Cherry Valley Duck | 272 | 1 |
| 2022.09.07 | Zhejiang | Duck | Cherry Valley Duck | 272 | 1 |
| 2022.09.07 | Zhejiang | Duck | Cherry Valley Duck | 272 | 1 |
| 2022.09.07 | Zhejiang | Duck | Cherry Valley Duck | 272 | 1 |
| 2022.09.07 | Zhejiang | Duck | Cherry Valley Duck | 272 | 1 |
| 2022.09.07 | Zhejiang | Duck | Cherry Valley Duck | 272 | 1 |
| 2022.09.07 | Zhejiang | Duck | Cherry Valley Duck | 272 | 1 |
| 2022.09.07 | Zhejiang | Duck | Cherry Valley Duck | 272 | 1 |
| 2022.09.07 | Zhejiang | Duck | Cherry Valley Duck | 272 | 1 |
| 2022.09.07 | Zhejiang | Duck | Cherry Valley Duck | 272 | 1 |
| 2022.09.07 | Zhejiang | Duck | Cherry Valley Duck | 272 | 1 |
| 2022.09.07 | Zhejiang | Duck | Cherry Valley Duck | 272 | 1 |
| 2022.09.07 | Zhejiang | Duck | Cherry Valley Duck | 272 | 1 |
| 2022.09.08 | Shandong | Duck | White Duck | 18 | 1 |
| 2022.09.08 | Jiangxi | Duck | Muscovy Duck | 19 | 1 |
| 2022.09.08 | Jiangxi | Duck | Muscovy Duck | 19 | 1 |
| 2022.09.08 | Jiangxi | Duck | Muscovy Duck | 19 | 1 |
| 2022.09.09 | Shandong | Duck | Cherry Valley Duck | 30 | 1 |
| 2022.09.09 | Shandong | Duck | Cherry Valley Duck | 30 | 1 |
| 2022.09.09 | Shandong | Duck | Cherry Valley Duck | 30 | 1 |
| 2022.09.09 | Shandong | Duck | Cherry Valley Duck | 30 | 1 |
| 2022.09.09 | Guangdong | Duck | White Duck | 35 | 1 |
| 2022.09.09 | Guangdong | Duck | White Duck | 35 | 1 |
| 2022.09.09 | Guangdong | Duck | White Duck | 35 | 1 |
| 2022.09.09 | Guangdong | Duck | White Duck | 35 | 1 |
| 2022.09.09 | Guangdong | Duck | White Duck | 35 | 1 |
| 2022.09.09 | Guangdong | Duck | White Duck | 35 | 1 |
| 2022.09.09 | Guangdong | Duck | White Duck | 35 | 1 |
| 2022.09.09 | Guangdong | Duck | White Duck | 35 | 1 |
| 2022.09.09 | Guangdong | Duck | White Duck | 35 | 1 |
| 2022.09.09 | Guangdong | Duck | White Duck | 35 | 1 |
| 2022.09.09 | Guangdong | Duck | White Duck | 35 | 1 |
| 2022.09.09 | Guangdong | Duck | White Duck | 35 | 1 |
| 2022.09.09 | Guangdong | Duck | White Duck | 35 | 1 |
| 2022.09.13 | Heilongjiang | Duck | / | 34 | 1 |
| 2022.09.13 | Anhui | Duck | / | 3 | 1 |
| 2022.09.13 | Anhui | Duck | / | 3 | 1 |
| 2022.09.13 | Anhui | Duck | / | 3 | 1 |
| 2022.09.13 | Anhui | Duck | / | 3 | 1 |
| 2022.09.13 | Anhui | Duck | / | 3 | 1 |
| 2022.09.13 | Anhui | Duck | / | 3 | 1 |
| 2022.09.13 | Anhui | Duck | / | 3 | 1 |
| 2022.09.13 | Anhui | Duck | / | 3 | 1 |
| 2022.09.13 | Jiangxi | Duck | Muscovy Duck | 9 | 1 |
| 2022.09.13 | Jiangxi | Duck | Muscovy Duck | 9 | 1 |
| 2022.09.15 | Guangdong | Duck | White Duck | / | 1 |
| 2022.09.15 | Guangdong | Duck | White Duck | / | 1 |
| 2022.09.15 | Guangdong | Duck | White Duck | / | 1 |
| 2022.09.15 | Guangdong | Duck | White Duck | / | 1 |
| 2022.09.15 | Guangdong | Duck | White Duck | / | 1 |
| 2022.09.15 | Guangdong | Duck | White Duck | / | 1 |
| 2022.09.15 | Guangdong | Duck | Shelduck | 16 | 1 |
| 2022.09.19 | Hebei | Duck | Cherry Valley Duck | 25 | 1 |
| 2022.09.19 | Hebei | Duck | Cherry Valley Duck | 25 | 1 |
| 2022.09.19 | Hebei | Duck | Cherry Valley Duck | 25 | 1 |
| 2022.09.19 | Hebei | Duck | Cherry Valley Duck | 25 | 1 |
| 2022.09.19 | Hebei | Duck | Cherry Valley Duck | 25 | 1 |
| 2022.09.19 | Hebei | Duck | Cherry Valley Duck | 25 | 1 |
| 2022.09.19 | Hebei | Duck | Cherry Valley Duck | 25 | 1 |
| 2022.09.19 | Hebei | Duck | Cherry Valley Duck | 25 | 1 |
| 2022.09.19 | Hebei | Duck | Cherry Valley Duck | 25 | 1 |
| 2022.09.19 | Hebei | Duck | Cherry Valley Duck | 25 | 1 |
| 2022.09.19 | Hebei | Duck | Cherry Valley Duck | 25 | 1 |
| 2022.09.19 | Hebei | Duck | Cherry Valley Duck | 25 | 1 |
| 2022.09.19 | Henan | Duck | / | 21 | 1 |
| 2022.09.19 | Henan | Duck | / | 21 | 1 |
| 2022.09.19 | Henan | Duck | / | 21 | 1 |
| 2022.09.19 | Henan | Duck | / | 21 | 1 |
| 2022.09.19 | Henan | Duck | / | 21 | 1 |
| 2022.09.19 | Henan | Duck | / | 21 | 1 |
| 2022.09.19 | Henan | Duck | / | 21 | 1 |
| 2022.09.19 | Henan | Duck | / | 21 | 1 |
| 2022.09.19 | Shandong | Duck | Cherry Valley Duck | 10 | 1 |
| 2022.09.19 | Shandong | Duck | Cherry Valley Duck | 10 | 1 |
| 2022.09.19 | Shandong | Duck | Cherry Valley Duck | 10 | 1 |
| 2022.09.19 | Shandong | Duck | Cherry Valley Duck | 10 | 1 |
| 2022.09.19 | Shandong | Duck | Cherry Valley Duck | 10 | 1 |
| 2022.09.19 | Shandong | Duck | Cherry Valley Duck | 10 | 1 |
| 2022.09.19 | Shandong | Duck | / | 3 | 1 |
| 2022.09.19 | Shandong | Duck | / | 3 | 1 |
| 2022.09.19 | Shandong | Duck | / | 3 | 1 |
| 2022.09.19 | Shandong | Duck | / | 3 | 1 |
| 2022.09.19 | Guangdong | Duck | / | 110 | 1 |
| 2022.09.19 | Guangdong | Duck | / | 110 | 1 |
| 2022.09.19 | Guangdong | Duck | / | 110 | 1 |
| 2022.09.19 | Jiangsu | Duck | Cherry Valley Duck | 70 | 1 |
| 2022.09.19 | Jiangsu | Duck | Cherry Valley Duck | 70 | 1 |
| 2022.09.19 | Jiangsu | Duck | Cherry Valley Duck | 70 | 1 |
| 2022.09.19 | Jiangsu | Duck | Cherry Valley Duck | 70 | 1 |
| 2022.09.19 | Jiangsu | Duck | Cherry Valley Duck | 70 | 1 |
| 2022.09.19 | Jiangsu | Duck | Cherry Valley Duck | 70 | 1 |
| 2022.09.19 | Jiangsu | Duck | Cherry Valley Duck | 70 | 1 |
| 2022.09.19 | Jiangsu | Duck | Cherry Valley Duck | 70 | 1 |
| 2022.09.19 | Jiangsu | Duck | Cherry Valley Duck | 70 | 1 |
| 2022.09.19 | Anhui | Duck | Cherry Valley Duck | 28 | 1 |
| 2022.09.19 | Anhui | Duck | Cherry Valley Duck | 28 | 1 |
| 2022.09.19 | Anhui | Duck | Cherry Valley Duck | 28 | 1 |
| 2022.09.19 | Anhui | Duck | Cherry Valley Duck | 28 | 1 |
| 2022.09.19 | Anhui | Duck | Cherry Valley Duck | 28 | 1 |
| 2022.09.19 | Anhui | Duck | Cherry Valley Duck | 28 | 1 |
| 2022.09.19 | Anhui | Duck | Cherry Valley Duck | 28 | 1 |
| 2022.09.19 | Anhui | Duck | Cherry Valley Duck | 28 | 1 |
| 2022.09.19 | Anhui | Duck | Cherry Valley Duck | 28 | 1 |
| 2022.09.19 | Jiangxi | Duck | Muscovy Duck | 85 | 1 |
| 2022.09.19 | Jiangxi | Duck | Muscovy Duck | 85 | 1 |
| 2022.09.19 | Jiangxi | Duck | Muscovy Duck | 85 | 1 |
| 2022.09.19 | Jiangxi | Duck | Muscovy Duck | 85 | 1 |
| 2022.09.19 | Jiangxi | Duck | Muscovy Duck | 85 | 1 |
| 2022.09.19 | Henan | Duck | Cherry Valley Duck | 38 | 1 |
| 2022.09.20 | Shandong | Duck | / | 31 | 1 |
| 2022.09.20 | Shandong | Duck | / | 31 | 1 |
| 2022.09.20 | Anhui | Duck | / | 3 | 1 |
| 2022.09.20 | Anhui | Duck | / | 3 | 1 |
| 2022.09.20 | Anhui | Duck | / | 3 | 1 |
| 2022.09.20 | Anhui | Duck | / | 3 | 1 |
| 2022.09.20 | Anhui | Duck | / | 3 | 1 |
| 2022.09.20 | Anhui | Duck | / | 3 | 1 |
| 2022.09.20 | Anhui | Duck | / | 3 | 1 |
| 2022.09.20 | Anhui | Duck | / | 3 | 1 |
| 2022.09.20 | Anhui | Duck | White Duck | 20 | 1 |
| 2022.09.20 | Anhui | Duck | White Duck | 20 | 1 |
| 2022.09.20 | Anhui | Duck | / | 33 | 1 |
| 2022.09.20 | Anhui | Duck | / | 33 | 1 |
| 2022.09.20 | Anhui | Duck | / | 33 | 1 |
| 2022.09.20 | Anhui | Duck | / | 33 | 1 |
| 2022.09.20 | Anhui | Duck | / | 33 | 1 |
| 2022.09.20 | Anhui | Duck | / | 33 | 1 |
| 2022.09.21 | Guangxi | Duck | Cherry Valley Duck | 24 | 1 |
| 2022.09.21 | Guangxi | Duck | Cherry Valley Duck | 24 | 1 |
| 2022.09.21 | Guangxi | Duck | Cherry Valley Duck | 24 | 1 |
| 2022.09.21 | Guangxi | Duck | Cherry Valley Duck | 24 | 1 |
| 2022.09.22 | Shandong | Duck | Cherry Valley Duck | 30 | 1 |
| 2022.09.22 | Shandong | Duck | Cherry Valley Duck | 30 | 1 |
| 2022.09.22 | Shandong | Duck | Cherry Valley Duck | 30 | 1 |
| 2022.09.22 | Shandong | Duck | Cherry Valley Duck | 30 | 1 |
| 2022.09.22 | Shandong | Duck | Cherry Valley Duck | 30 | 1 |
| 2022.09.22 | Shandong | Duck | Cherry Valley Duck | 30 | 1 |
| 2022.09.22 | Shandong | Duck | Cherry Valley Duck | 30 | 1 |
| 2022.09.22 | Shandong | Duck | Cherry Valley Duck | 30 | 1 |
| 2022.09.22 | Shandong | Duck | Cherry Valley Duck | 30 | 1 |
| 2022.09.22 | Shandong | Duck | Cherry Valley Duck | 30 | 1 |
| 2022.09.23 | Hebei | Duck | Cherry Valley Duck | 28 | 1 |
| 2022.09.23 | Hebei | Duck | Cherry Valley Duck | 35 | 1 |
| 2022.09.23 | Hebei | Duck | Cherry Valley Duck | 35 | 1 |
| 2022.09.23 | Hebei | Duck | Cherry Valley Duck | 35 | 1 |
| 2022.09.23 | Hebei | Duck | Cherry Valley Duck | 35 | 1 |
| 2022.09.23 | Hebei | Duck | Cherry Valley Duck | 35 | 1 |
| 2022.09.23 | Hebei | Duck | Cherry Valley Duck | 35 | 1 |
| 2022.09.23 | Hebei | Duck | Cherry Valley Duck | 35 | 1 |
| 2022.09.23 | Hebei | Duck | Cherry Valley Duck | 35 | 1 |
| 2022.09.23 | Hebei | Duck | Cherry Valley Duck | 35 | 1 |
| 2022.09.23 | Hebei | Duck | Cherry Valley Duck | 35 | 1 |
| 2022.09.23 | Hebei | Duck | Cherry Valley Duck | 35 | 1 |
| 2022.09.23 | Hebei | Duck | Cherry Valley Duck | 35 | 1 |
| 2022.09.24 | Anhui | Duck | Cherry Valley Duck | 27 | 1 |
| 2022.09.24 | Anhui | Duck | Muscovy Duck | 30 | 1 |
| 2022.09.26 | Shandong | Duck | Cherry Valley Duck | 22 | 1 |
| 2022.09.26 | Shandong | Duck | Cherry Valley Duck | 22 | 1 |
| 2022.09.26 | Shandong | Duck | Cherry Valley Duck | 22 | 1 |
| 2022.09.26 | Shandong | Duck | Cherry Valley Duck | 22 | 1 |
| 2022.09.26 | Shandong | Duck | Cherry Valley Duck | 22 | 1 |
| 2022.09.26 | Shandong | Duck | Cherry Valley Duck | 22 | 1 |
| 2022.09.26 | Shandong | Duck | Cherry Valley Duck | 22 | 1 |
| 2022.09.26 | Shandong | Duck | Cherry Valley Duck | 22 | 1 |
| 2022.09.26 | Shandong | Duck | Cherry Valley Duck | 22 | 1 |
| 2022.09.26 | Shandong | Duck | Cherry Valley Duck | 22 | 1 |
| 2022.09.26 | Shandong | Duck | Cherry Valley Duck | 329 | 1 |
| 2022.09.26 | Anhui | Duck | / | 32 | 1 |
| 2022.09.27 | Shandong | Duck | White Duck | 23 | 1 |
| 2022.09.27 | Shandong | Duck | White Duck | 23 | 1 |
| 2022.09.27 | Shandong | Duck | White Duck | 23 | 1 |
| 2022.09.27 | Guangdong | Duck | Muscovy Duck | 63 | 1 |
| 2022.09.27 | Guangdong | Duck | Muscovy Duck | 63 | 1 |
| 2022.09.27 | Guangdong | Duck | Muscovy Duck | 63 | 1 |
| 2022.09.27 | Guangdong | Duck | Muscovy Duck | 63 | 1 |
| 2022.09.27 | Guangdong | Duck | Muscovy Duck | 63 | 1 |
| 2022.09.29 | Shandong | Duck | Cherry Valley Duck | 30 | 1 |
| 2022.09.29 | Shandong | Duck | Cherry Valley Duck | 30 | 1 |
| 2022.09.29 | Guangdong | Duck | Muscovy Duck | 400 | 1 |
| 2022.09.29 | Guangdong | Duck | White Duck | 40 | 1 |
| 2022.09.29 | Guangdong | Duck | White Duck | 40 | 1 |
| 2022.09.29 | Guangdong | Duck | White Duck | 40 | 1 |
| 2022.09.30 | Sichuan | Duck | / | 31 | 1 |
| 2022.09.30 | Sichuan | Duck | / | 31 | 1 |
| 2022.09.30 | Sichuan | Duck | / | 31 | 1 |
| 2022.09.30 | Sichuan | Duck | / | 31 | 1 |
| 2022.09.30 | Guangxi | Duck | Cherry Valley Duck | 30 | 1 |
| 2022.09.30 | Guangxi | Duck | Cherry Valley Duck | 30 | 1 |
| 2022.09.30 | Jiangsu | Duck | Cherry Valley Duck | 34 | 1 |
| 2022.09.30 | Jiangsu | Duck | Cherry Valley Duck | 9 | 1 |
| 2022.09.30 | Guangdong | Duck | Shelduck | 45 | 1 |
| 2022.09.30 | Guangdong | Duck | White Duck | 22 | 1 |
| 2022.09.30 | Guangdong | Duck | White Duck | 22 | 1 |
| 2022.09.30 | Guangdong | Duck | White Duck | 22 | 1 |
| 2022.09.30 | Guangdong | Duck | White Duck | 22 | 1 |
| 2022.1.12 | Shandong | Duck | / | 21 | 1 |
| 2022.10.05 | Shandong | Duck | Cherry Valley Duck | 22 | 1 |
| 2022.10.05 | Shandong | Duck | Cherry Valley Duck | 22 | 1 |
| 2022.10.05 | Shandong | Duck | Cherry Valley Duck | 22 | 1 |
| 2022.10.05 | Shandong | Duck | Cherry Valley Duck | 22 | 1 |
| 2022.10.05 | Shandong | Duck | Cherry Valley Duck | 22 | 1 |
| 2022.10.05 | Shandong | Duck | Cherry Valley Duck | 22 | 1 |
| 2022.10.05 | Shandong | Duck | Cherry Valley Duck | 22 | 1 |
| 2022.10.05 | Shandong | Duck | Cherry Valley Duck | 22 | 1 |
| 2022.10.05 | Shandong | Duck | Cherry Valley Duck | 22 | 1 |
| 2022.10.05 | Shandong | Duck | Cherry Valley Duck | 22 | 1 |
| 2022.10.05 | Shandong | Duck | Cherry Valley Duck | 22 | 1 |
| 2022.10.05 | Shandong | Duck | Cherry Valley Duck | 22 | 1 |
| 2022.10.05 | Shandong | Duck | Cherry Valley Duck | 22 | 1 |
| 2022.10.05 | Shandong | Duck | Cherry Valley Duck | 22 | 1 |
| 2022.10.05 | Shandong | Duck | Cherry Valley Duck | 22 | 1 |
| 2022.10.05 | Shandong | Duck | Cherry Valley Duck | 22 | 1 |
| 2022.10.05 | Shandong | Duck | Cherry Valley Duck | 22 | 1 |
| 2022.10.05 | Shandong | Duck | Cherry Valley Duck | 22 | 1 |
| 2022.10.05 | Shandong | Duck | Cherry Valley Duck | 22 | 1 |
| 2022.10.05 | Shandong | Duck | Cherry Valley Duck | 22 | 1 |
| 2022.10.05 | Shandong | Duck | Cherry Valley Duck | 22 | 1 |
| 2022.10.05 | Shandong | Duck | Cherry Valley Duck | 22 | 1 |
| 2022.10.05 | Shandong | Duck | Cherry Valley Duck | 22 | 1 |
| 2022.10.05 | Shandong | Duck | Cherry Valley Duck | 31 | 1 |
| 2022.10.05 | Shandong | Duck | Cherry Valley Duck | 30 | 1 |
| 2022.10.05 | Jiangxi | Duck | / | / | 1 |
| 2022.10.05 | Jiangxi | Duck | Muscovy Duck | 8 | 1 |
| 2022.10.05 | Jiangxi | Duck | Muscovy Duck | 8 | 1 |
| 2022.10.05 | Jiangxi | Duck | Muscovy Duck | 8 | 1 |
| 2022.10.05 | Guangdong | Duck | White Duck | 38 | 1 |
| 2022.10.05 | Guangdong | Duck | White Duck | 38 | 1 |
| 2022.10.05 | Guangdong | Duck | White Duck | 38 | 1 |
| 2022.10.05 | Guangdong | Duck | White Duck | 38 | 1 |
| 2022.10.05 | Guangdong | Duck | White Duck | 38 | 1 |
| 2022.10.05 | Guangdong | Duck | White Duck | 38 | 1 |
| 2022.10.05 | Guangdong | Duck | White Duck | 38 | 1 |
| 2022.10.05 | Guangdong | Duck | White Duck | 38 | 1 |
| 2022.10.05 | Guangdong | Duck | White Duck | 38 | 1 |
| 2022.10.05 | Guangdong | Duck | White Duck | 38 | 1 |
| 2022.10.05 | Guangdong | Duck | White Duck | 38 | 1 |
| 2022.10.05 | Guangdong | Duck | White Duck | 14 | 1 |
| 2022.10.05 | Guangdong | Duck | White Duck | 14 | 1 |
| 2022.10.05 | Guangdong | Duck | White Duck | 14 | 1 |
| 2022.10.05 | Guangdong | Duck | White Duck | 14 | 1 |
| 2022.10.07 | Shandong | Duck | / | 3 | 1 |
| 2022.10.07 | Shandong | Duck | / | 3 | 1 |
| 2022.10.07 | Shandong | Duck | / | 3 | 1 |
| 2022.10.07 | Shandong | Duck | / | 3 | 1 |
| 2022.10.07 | Shandong | Duck | / | 3 | 1 |
| 2022.10.07 | Shandong | Duck | / | 3 | 1 |
| 2022.10.07 | Jiangxi | Duck | Muscovy Duck | 6 | 1 |
| 2022.10.07 | Jiangxi | Duck | Muscovy Duck | 6 | 1 |
| 2022.10.10 | Anhui | Duck | / | 3 | 1 |
| 2022.10.10 | Anhui | Duck | / | 3 | 1 |
| 2022.10.10 | Anhui | Duck | / | 3 | 1 |
| 2022.10.10 | Anhui | Duck | / | 3 | 1 |
| 2022.10.10 | Anhui | Duck | / | 3 | 1 |
| 2022.10.10 | Anhui | Duck | / | 3 | 1 |
| 2022.10.10 | Anhui | Duck | / | 3 | 1 |
| 2022.10.10 | Anhui | Duck | / | 3 | 1 |
| 2022.10.10 | Shandong | Duck | / | 3 | 1 |
| 2022.10.10 | Shandong | Duck | / | 3 | 1 |
| 2022.10.10 | Shandong | Duck | / | 3 | 1 |
| 2022.10.10 | Shandong | Duck | / | 3 | 1 |
| 2022.10.10 | Shandong | Duck | Cherry Valley Duck | 25 | 1 |
| 2022.10.10 | Shandong | Duck | Cherry Valley Duck | 25 | 1 |
| 2022.10.10 | Shandong | Duck | Cherry Valley Duck | 25 | 1 |
| 2022.10.10 | Shandong | Duck | Cherry Valley Duck | 25 | 1 |
| 2022.10.10 | Shandong | Duck | Cherry Valley Duck | 25 | 1 |
| 2022.10.10 | Guangdong | Duck | White Duck | 34 | 1 |
| 2022.10.10 | Guangdong | Duck | White Duck | 34 | 1 |
| 2022.10.10 | Guangdong | Duck | White Duck | 34 | 1 |
| 2022.10.10 | Guangdong | Duck | White Duck | 34 | 1 |
| 2022.10.10 | Guangdong | Duck | White Duck | 24 | 1 |
| 2022.10.10 | Guangdong | Duck | White Duck | 24 | 1 |
| 2022.10.10 | Guangdong | Duck | White Duck | 24 | 1 |
| 2022.10.10 | Guangdong | Duck | White Duck | 24 | 1 |
| 2022.10.11 | Shannxi | Duck | Peking Duck | 3 | 1 |
| 2022.10.11 | Shannxi | Duck | Peking Duck | 3 | 1 |
| 2022.10.11 | Shannxi | Duck | Peking Duck | 3 | 1 |
| 2022.10.11 | Shannxi | Duck | Peking Duck | 3 | 1 |
| 2022.10.11 | Shannxi | Duck | Peking Duck | 3 | 1 |
| 2022.10.11 | Shannxi | Duck | Peking Duck | 3 | 1 |
| 2022.10.11 | Shannxi | Duck | Peking Duck | 3 | 1 |
| 2022.10.11 | Shannxi | Duck | Peking Duck | 3 | 1 |
| 2022.10.11 | Shandong | Duck | / | 31 | 1 |
| 2022.10.11 | Hebei | Duck | Cherry Valley Duck | 30 | 1 |
| 2022.10.11 | Hebei | Duck | Cherry Valley Duck | 30 | 1 |
| 2022.10.14 | Shandong | Duck | / | Duck embryos | 1 |
| 2022.10.14 | Shandong | Duck | / | 25 | 1 |
| 2022.10.17 | Hebei | Duck | / | / | 1 |
| 2022.10.17 | Hebei | Duck | / | / | 1 |
| 2022.10.17 | Hebei | Duck | / | / | 1 |
| 2022.10.17 | Hebei | Duck | / | / | 1 |
| 2022.10.17 | Hebei | Duck | / | / | 1 |
| 2022.10.17 | Hebei | Duck | / | / | 1 |
| 2022.10.17 | Hebei | Duck | / | / | 1 |
| 2022.10.17 | Hebei | Duck | / | / | 1 |
| 2022.10.17 | Hebei | Duck | / | / | 1 |
| 2022.10.17 | Hebei | Duck | / | / | 1 |
| 2022.10.17 | Hebei | Duck | / | / | 1 |
| 2022.10.17 | Hebei | Duck | / | / | 1 |
| 2022.10.17 | Hebei | Duck | / | / | 1 |
| 2022.10.17 | Hebei | Duck | / | / | 1 |
| 2022.10.17 | Hebei | Duck | / | / | 1 |
| 2022.10.17 | Henan | Duck | Cherry Valley Duck | / | 1 |
| 2022.10.17 | Henan | Duck | Jinding Duck | 15 | 1 |
| 2022.10.17 | Anhui | Duck | / | 3 | 1 |
| 2022.10.17 | Anhui | Duck | / | 3 | 1 |
| 2022.10.17 | Anhui | Duck | / | 3 | 1 |
| 2022.10.17 | Anhui | Duck | / | 3 | 1 |
| 2022.10.17 | Anhui | Duck | / | 3 | 1 |
| 2022.10.17 | Anhui | Duck | / | 3 | 1 |
| 2022.10.17 | Jiangxi | Duck | / | 16 | 1 |
| 2022.10.17 | Jiangxi | Duck | / | 16 | 1 |
| 2022.10.17 | Jiangxi | Duck | / | 16 | 1 |
| 2022.10.17 | Henan | Duck | / | / | 1 |
| 2022.10.17 | Henan | Duck | / | / | 1 |
| 2022.10.17 | Guangdong | Duck | Muscovy Duck | / | 1 |
| 2022.10.17 | Guangdong | Duck | Muscovy Duck | / | 1 |
| 2022.10.17 | Guangdong | Duck | Muscovy Duck | / | 1 |
| 2022.10.17 | Guangdong | Duck | Muscovy Duck | / | 1 |
| 2022.10.17 | Guangdong | Duck | / | 300 | 1 |
| 2022.10.17 | Guangdong | Duck | / | 300 | 1 |
| 2022.10.17 | Guangdong | Duck | / | 300 | 1 |
| 2022.10.17 | Guangdong | Duck | / | 300 | 1 |
| 2022.10.17 | Anhui | Duck | Cherry Valley Duck | 40 | 1 |
| 2022.10.17 | Shandong | Duck | / | 35 | 1 |
| 2022.10.17 | Shandong | Duck | / | 35 | 1 |
| 2022.10.18 | Guangxi | Duck | Cherry Valley Duck | 34 | 1 |
| 2022.10.18 | Guangxi | Duck | Cherry Valley Duck | 34 | 1 |
| 2022.10.18 | Guangxi | Duck | Cherry Valley Duck | 34 | 1 |
| 2022.10.18 | Guangxi | Duck | Cherry Valley Duck | 34 | 1 |
| 2022.10.18 | Guangxi | Duck | Cherry Valley Duck | 34 | 1 |
| 2022.10.18 | Guangxi | Duck | Cherry Valley Duck | 34 | 1 |
| 2022.10.18 | Guangxi | Duck | Cherry Valley Duck | 34 | 1 |
| 2022.10.18 | Guangxi | Duck | Cherry Valley Duck | 34 | 1 |
| 2022.10.18 | Jiangxi | Duck | Muscovy Duck | 21 | 1 |
| 2022.10.18 | Jiangxi | Duck | Muscovy Duck | 21 | 1 |
| 2022.10.18 | Guangdong | Duck | Muscovy Duck | 58 | 1 |
| 2022.10.18 | Guangdong | Duck | Muscovy Duck | 58 | 1 |
| 2022.10.18 | Guangdong | Duck | Muscovy Duck | 58 | 1 |
| 2022.10.18 | Guangdong | Duck | Muscovy Duck | 58 | 1 |
| 2022.10.18 | Guangdong | Duck | Muscovy Duck | 58 | 1 |
| 2022.10.18 | Guangdong | Duck | Muscovy Duck | 58 | 1 |
| 2022.10.18 | Guangdong | Duck | Muscovy Duck | 58 | 1 |
| 2022.10.18 | Guangdong | Duck | Muscovy Duck | 58 | 1 |
| 2022.10.18 | Guangdong | Duck | Muscovy Duck | 58 | 1 |
| 2022.10.18 | Guangdong | Duck | Muscovy Duck | 58 | 1 |
| 2022.10.18 | Guangdong | Duck | Muscovy Duck | 58 | 1 |
| 2022.10.18 | Guangdong | Duck | Muscovy Duck | 58 | 1 |
| 2022.10.18 | Guangdong | Duck | Muscovy Duck | 58 | 1 |
| 2022.10.18 | Guangdong | Duck | Muscovy Duck | 58 | 1 |
| 2022.10.18 | Jiangsu | Duck | / | 69 | 1 |
| 2022.10.18 | Jiangsu | Duck | / | 69 | 1 |
| 2022.10.18 | Jiangsu | Duck | / | 69 | 1 |
| 2022.10.18 | Jiangsu | Duck | / | 69 | 1 |
| 2022.10.18 | Jiangsu | Duck | / | 69 | 1 |
| 2022.10.18 | Jiangsu | Duck | / | 69 | 1 |
| 2022.10.20 | Shandong | Duck | / | 36 | 1 |
| 2022.10.20 | Sichuan | Duck | / | 15 | 1 |
| 2022.10.20 | Anhui | Duck | / | 24 | 1 |
| 2022.10.20 | Guangdong | Duck | Muscovy Duck | 8 | 1 |
| 2022.10.20 | Guangdong | Duck | Muscovy Duck | 8 | 1 |
| 2022.10.20 | Jiangxi | Duck | Muscovy Duck | 19 | 1 |
| 2022.10.20 | Jiangxi | Duck | Muscovy Duck | 19 | 1 |
| 2022.10.20 | Shandong | Duck | / | 31 | 1 |
| 2022.10.20 | Shandong | Duck | / | 31 | 1 |
| 2022.10.20 | Shandong | Duck | / | 32 | 1 |
| 2022.10.21 | Shandong | Duck | / | / | 1 |
| 2022.10.21 | Shandong | Duck | / | / | 1 |
| 2022.10.21 | Shandong | Duck | / | / | 1 |
| 2022.10.21 | Shandong | Duck | / | / | 1 |
| 2022.10.21 | Shandong | Duck | / | / | 1 |
| 2022.10.21 | Shandong | Duck | / | / | 1 |
| 2022.10.21 | Shandong | Duck | / | / | 1 |
| 2022.10.21 | Shandong | Duck | / | / | 1 |
| 2022.10.21 | Shandong | Duck | / | / | 1 |
| 2022.10.21 | Shandong | Duck | / | / | 1 |
| 2022.10.21 | Shandong | Duck | / | / | 1 |
| 2022.10.21 | Shandong | Duck | / | / | 1 |
| 2022.10.21 | Shandong | Duck | / | / | 1 |
| 2022.10.21 | Shandong | Duck | / | / | 1 |
| 2022.10.21 | Shandong | Duck | / | / | 1 |
| 2022.10.21 | Anhui | Duck | / | 34 | 1 |
| 2022.10.23 | Shandong | Duck | / | 30 | 1 |
| 2022.10.24 | Shandong | Duck | / | 25 | 1 |
| 2022.10.24 | Shandong | Duck | / | 30 | 1 |
| 2022.10.24 | Shandong | Duck | / | 3 | 1 |
| 2022.10.24 | Shandong | Duck | / | 3 | 1 |
| 2022.10.24 | Shandong | Duck | / | 3 | 1 |
| 2022.10.24 | Shandong | Duck | / | 3 | 1 |
| 2022.10.24 | Shandong | Duck | / | 3 | 1 |
| 2022.10.24 | Shandong | Duck | / | 3 | 1 |
| 2022.10.24 | Nei Mongol | Duck | / | 35 | 1 |
| 2022.10.24 | Nei Mongol | Duck | / | 35 | 1 |
| 2022.10.24 | Nei Mongol | Duck | / | 35 | 1 |
| 2022.10.24 | Nei Mongol | Duck | / | 35 | 1 |
| 2022.10.24 | Nei Mongol | Duck | / | 35 | 1 |
| 2022.10.24 | Nei Mongol | Duck | / | 35 | 1 |
| 2022.10.24 | Shandong | Duck | Cherry Valley Duck | 1 | 1 |
| 2022.10.24 | Shandong | Duck | Cherry Valley Duck | 1 | 1 |
| 2022.10.24 | Shandong | Duck | Cherry Valley Duck | 1 | 1 |
| 2022.10.24 | Shandong | Duck | Cherry Valley Duck | 1 | 1 |
| 2022.10.24 | Shandong | Duck | Cherry Valley Duck | 1 | 1 |
| 2022.10.24 | Shandong | Duck | Cherry Valley Duck | 1 | 1 |
| 2022.10.24 | Shandong | Duck | Cherry Valley Duck | 1 | 1 |
| 2022.10.24 | Shandong | Duck | Cherry Valley Duck | 1 | 1 |
| 2022.10.24 | Shandong | Duck | Cherry Valley Duck | 1 | 1 |
| 2022.10.24 | Shandong | Duck | Cherry Valley Duck | 1 | 1 |
| 2022.10.24 | Shandong | Duck | Cherry Valley Duck | 1 | 1 |
| 2022.10.24 | Shandong | Duck | Cherry Valley Duck | 1 | 1 |
| 2022.10.24 | Shandong | Duck | Cherry Valley Duck | 1 | 1 |
| 2022.10.24 | Shandong | Duck | Cherry Valley Duck | 1 | 1 |
| 2022.10.24 | Shandong | Duck | Cherry Valley Duck | 1 | 1 |
| 2022.10.24 | Shandong | Duck | Cherry Valley Duck | 1 | 1 |
| 2022.10.24 | Shandong | Duck | Cherry Valley Duck | 1 | 1 |
| 2022.10.24 | Shandong | Duck | Cherry Valley Duck | 1 | 1 |
| 2022.10.24 | Shandong | Duck | Cherry Valley Duck | 1 | 1 |
| 2022.10.24 | Shandong | Duck | Cherry Valley Duck | 1 | 1 |
| 2022.10.24 | Shandong | Duck | Cherry Valley Duck | 1 | 1 |
| 2022.10.24 | Shandong | Duck | Cherry Valley Duck | 1 | 1 |
| 2022.10.24 | Shandong | Duck | Cherry Valley Duck | 1 | 1 |
| 2022.10.24 | Shandong | Duck | Cherry Valley Duck | 1 | 1 |
| 2022.10.24 | Shandong | Duck | Cherry Valley Duck | 1 | 1 |
| 2022.10.24 | Shandong | Duck | Cherry Valley Duck | 1 | 1 |
| 2022.10.24 | Shandong | Duck | Cherry Valley Duck | 1 | 1 |
| 2022.10.24 | Shandong | Duck | Cherry Valley Duck | 1 | 1 |
| 2022.10.24 | Shandong | Duck | Cherry Valley Duck | 1 | 1 |
| 2022.10.24 | Shandong | Duck | Cherry Valley Duck | 1 | 1 |
| 2022.10.24 | Shandong | Duck | Cherry Valley Duck | 1 | 1 |
| 2022.10.24 | Shandong | Duck | Cherry Valley Duck | 1 | 1 |
| 2022.10.24 | Shandong | Duck | Cherry Valley Duck | 1 | 1 |
| 2022.10.24 | Shandong | Duck | Cherry Valley Duck | 1 | 1 |
| 2022.10.24 | Shandong | Duck | Cherry Valley Duck | 1 | 1 |
| 2022.10.24 | Shandong | Duck | Cherry Valley Duck | 7 | 1 |
| 2022.10.24 | Guangdong | Duck | Muscovy Duck | 25 | 1 |
| 2022.10.24 | Guangdong | Duck | Muscovy Duck | 25 | 1 |
| 2022.10.24 | Guangdong | Duck | Muscovy Duck | 25 | 1 |
| 2022.10.24 | Guangdong | Duck | Muscovy Duck | 25 | 1 |
| 2022.10.24 | Guangdong | Duck | White Duck | 33 | 1 |
| 2022.10.24 | Guangdong | Duck | White Duck | 33 | 1 |
| 2022.10.24 | Guangdong | Duck | White Duck | 33 | 1 |
| 2022.10.24 | Guangdong | Duck | White Duck | 33 | 1 |
| 2022.10.24 | Guangdong | Duck | White Duck | 33 | 1 |
| 2022.10.24 | Guangdong | Duck | White Duck | 33 | 1 |
| 2022.10.24 | Guangdong | Duck | White Duck | 33 | 1 |
| 2022.10.24 | Guangdong | Duck | White Duck | 33 | 1 |
| 2022.10.24 | Guangdong | Duck | White Duck | 33 | 1 |
| 2022.10.25 | Shandong | Duck | Muscovy Duck | 20 | 1 |
| 2022.10.25 | Shandong | Duck | Cherry Valley Duck | 23 | 1 |
| 2022.10.25 | Shandong | Duck | Cherry Valley Duck | 23 | 1 |
| 2022.10.25 | Shandong | Duck | Cherry Valley Duck | 23 | 1 |
| 2022.10.25 | Shandong | Duck | Cherry Valley Duck | 23 | 1 |
| 2022.10.25 | Hebei | Duck | / | 28 | 1 |
| 2022.10.25 | Hebei | Duck | / | 28 | 1 |
| 2022.10.26 | Guangdong | Duck | Muscovy Duck | 40 | 1 |
| 2022.10.26 | Guangdong | Duck | Muscovy Duck | 40 | 1 |
| 2022.10.26 | Guangdong | Duck | Muscovy Duck | 40 | 1 |
| 2022.10.26 | Shandong | Duck | / | 35 | 1 |
| 2022.10.26 | Shandong | Duck | / | 33 | 1 |
| 2022.10.27 | Nei Mongol | Duck | Cherry Valley Duck | 250 | 1 |
| 2022.10.27 | Anhui | Duck | / | 23 | 1 |
| 2022.10.27 | Anhui | Duck | / | 23 | 1 |
| 2022.10.27 | Anhui | Duck | / | 23 | 1 |
| 2022.10.27 | Anhui | Duck | / | 23 | 1 |
| 2022.10.27 | Anhui | Duck | / | 23 | 1 |
| 2022.10.27 | Anhui | Duck | / | 23 | 1 |
| 2022.10.27 | Anhui | Duck | / | 23 | 1 |
| 2022.10.27 | Anhui | Duck | / | 23 | 1 |
| 2022.10.27 | Anhui | Duck | / | 38 | 1 |
| 2022.10.29 | Anhui | Duck | Muscovy Duck | 55 | 1 |
| 2022.10.29 | Anhui | Duck | Muscovy Duck | 55 | 1 |
| 2022.10.29 | Anhui | Duck | Muscovy Duck | 55 | 1 |
| 2022.10.29 | Anhui | Duck | Muscovy Duck | 55 | 1 |
| 2022.10.29 | Shandong | Duck | / | 33 | 1 |
| 2022.10.29 | Hebei | Duck | / | 22 | 1 |
| 2022.10.29 | Hebei | Duck | / | 30 | 1 |
| 2022.10.29 | Shanxi | Duck | / | 41 | 1 |
| 2022.10.30 | Anhui | Duck | / | 25 | 1 |
| 2022.10.31 | Shandong | Duck | Cherry Valley Duck | 0 | 1 |
| 2022.10.31 | Shandong | Duck | Cherry Valley Duck | 0 | 1 |
| 2022.10.31 | Shandong | Duck | Cherry Valley Duck | 0 | 1 |
| 2022.10.31 | Shandong | Duck | Cherry Valley Duck | 0 | 1 |
| 2022.10.31 | Shandong | Duck | Cherry Valley Duck | 0 | 1 |
| 2022.10.31 | Shandong | Duck | Cherry Valley Duck | 0 | 1 |
| 2022.10.31 | Shandong | Duck | Cherry Valley Duck | 0 | 1 |
| 2022.10.31 | Shandong | Duck | Cherry Valley Duck | 0 | 1 |
| 2022.10.31 | Shandong | Duck | Cherry Valley Duck | 0 | 1 |
| 2022.10.31 | Shandong | Duck | Cherry Valley Duck | 0 | 1 |
| 2022.10.31 | Shandong | Duck | Cherry Valley Duck | 0 | 1 |
| 2022.10.31 | Shandong | Duck | Cherry Valley Duck | 0 | 1 |
| 2022.10.31 | Shandong | Duck | Cherry Valley Duck | 0 | 1 |
| 2022.10.31 | Shandong | Duck | Cherry Valley Duck | 0 | 1 |
| 2022.10.31 | Shandong | Duck | Cherry Valley Duck | 0 | 1 |
| 2022.10.31 | Shandong | Duck | Cherry Valley Duck | 0 | 1 |
| 2022.10.31 | Shandong | Duck | Cherry Valley Duck | 0 | 1 |
| 2022.10.31 | Shandong | Duck | Cherry Valley Duck | 0 | 1 |
| 2022.10.31 | Shandong | Duck | Cherry Valley Duck | 0 | 1 |
| 2022.10.31 | Shandong | Duck | Cherry Valley Duck | 0 | 1 |
| 2022.10.31 | Shandong | Duck | Cherry Valley Duck | 0 | 1 |
| 2022.10.31 | Shandong | Duck | Cherry Valley Duck | 0 | 1 |
| 2022.10.31 | Shandong | Duck | Cherry Valley Duck | 0 | 1 |
| 2022.10.31 | Shandong | Duck | Cherry Valley Duck | 0 | 1 |
| 2022.10.31 | Shandong | Duck | Cherry Valley Duck | 0 | 1 |
| 2022.10.31 | Shandong | Duck | Cherry Valley Duck | 0 | 1 |
| 2022.10.31 | Shandong | Duck | Cherry Valley Duck | 0 | 1 |
| 2022.10.31 | Shandong | Duck | Cherry Valley Duck | 0 | 1 |
| 2022.10.31 | Shandong | Duck | Cherry Valley Duck | 0 | 1 |
| 2022.10.31 | Shandong | Duck | Cherry Valley Duck | 0 | 1 |
| 2022.10.31 | Shandong | Duck | Cherry Valley Duck | 0 | 1 |
| 2022.10.31 | Shandong | Duck | Cherry Valley Duck | 0 | 1 |
| 2022.10.31 | Shandong | Duck | Cherry Valley Duck | 0 | 1 |
| 2022.10.31 | Shandong | Duck | Cherry Valley Duck | 0 | 1 |
| 2022.10.31 | Shandong | Duck | Cherry Valley Duck | 0 | 1 |
| 2022.10.31 | Shandong | Duck | Cherry Valley Duck | 0 | 1 |
| 2022.10.31 | Shandong | Duck | Cherry Valley Duck | 0 | 1 |
| 2022.10.31 | Shandong | Duck | Cherry Valley Duck | 0 | 1 |
| 2022.10.31 | Shandong | Duck | Cherry Valley Duck | 0 | 1 |
| 2022.10.31 | Shandong | Duck | Cherry Valley Duck | 0 | 1 |
| 2022.10.31 | Shandong | Duck | Cherry Valley Duck | 0 | 1 |
| 2022.10.31 | Shandong | Duck | Cherry Valley Duck | 0 | 1 |
| 2022.10.31 | Shandong | Duck | Cherry Valley Duck | 0 | 1 |
| 2022.10.31 | Shandong | Duck | Cherry Valley Duck | 0 | 1 |
| 2022.10.31 | Shandong | Duck | Cherry Valley Duck | 0 | 1 |
| 2022.10.31 | Shandong | Duck | Cherry Valley Duck | 0 | 1 |
| 2022.10.31 | Shandong | Duck | Cherry Valley Duck | 0 | 1 |
| 2022.10.31 | Shandong | Duck | Cherry Valley Duck | 0 | 1 |
| 2022.10.31 | Shandong | Duck | Cherry Valley Duck | 0 | 1 |
| 2022.10.31 | Shandong | Duck | Cherry Valley Duck | 0 | 1 |
| 2022.10.31 | Shandong | Duck | Cherry Valley Duck | 0 | 1 |
| 2022.10.31 | Shandong | Duck | Cherry Valley Duck | 0 | 1 |
| 2022.10.31 | Shandong | Duck | Cherry Valley Duck | 0 | 1 |
| 2022.10.31 | Shandong | Duck | Cherry Valley Duck | 0 | 1 |
| 2022.10.31 | Shandong | Duck | Cherry Valley Duck | 0 | 1 |
| 2022.10.31 | Shandong | Duck | Cherry Valley Duck | 0 | 1 |
| 2022.10.31 | Shandong | Duck | Cherry Valley Duck | 0 | 1 |
| 2022.10.31 | Shandong | Duck | Cherry Valley Duck | 0 | 1 |
| 2022.10.31 | Shandong | Duck | Cherry Valley Duck | 0 | 1 |
| 2022.10.31 | Shandong | Duck | Cherry Valley Duck | 0 | 1 |
| 2022.10.31 | Shandong | Duck | Cherry Valley Duck | 0 | 1 |
| 2022.10.31 | Shandong | Duck | Cherry Valley Duck | 0 | 1 |
| 2022.10.31 | Shandong | Duck | Cherry Valley Duck | 0 | 1 |
| 2022.10.31 | Shandong | Duck | Cherry Valley Duck | 0 | 1 |
| 2022.10.31 | Shandong | Duck | Cherry Valley Duck | 0 | 1 |
| 2022.10.31 | Shandong | Duck | Cherry Valley Duck | 0 | 1 |
| 2022.10.31 | Shandong | Duck | Cherry Valley Duck | 0 | 1 |
| 2022.10.31 | Shandong | Duck | Cherry Valley Duck | 0 | 1 |
| 2022.10.31 | Shandong | Duck | Cherry Valley Duck | 0 | 1 |
| 2022.10.31 | Shandong | Duck | Cherry Valley Duck | 0 | 1 |
| 2022.10.31 | Shandong | Duck | Cherry Valley Duck | 0 | 1 |
| 2022.10.31 | Shandong | Duck | Cherry Valley Duck | 0 | 1 |
| 2022.10.31 | Shandong | Duck | Cherry Valley Duck | 0 | 1 |
| 2022.10.31 | Shandong | Duck | Cherry Valley Duck | 0 | 1 |
| 2022.10.31 | Shandong | Duck | Cherry Valley Duck | 0 | 1 |
| 2022.10.31 | Shandong | Duck | Cherry Valley Duck | 0 | 1 |
| 2022.10.31 | Shandong | Duck | Cherry Valley Duck | 0 | 1 |
| 2022.10.31 | Shandong | Duck | Cherry Valley Duck | 0 | 1 |
| 2022.10.31 | Shandong | Duck | Cherry Valley Duck | 0 | 1 |
| 2022.10.31 | Shandong | Duck | Cherry Valley Duck | 0 | 1 |
| 2022.10.31 | Shandong | Duck | Cherry Valley Duck | 0 | 1 |
| 2022.10.31 | Shandong | Duck | Cherry Valley Duck | 0 | 1 |
| 2022.10.31 | Shandong | Duck | Cherry Valley Duck | 0 | 1 |
| 2022.10.31 | Shandong | Duck | Cherry Valley Duck | 0 | 1 |
| 2022.10.31 | Shandong | Duck | Cherry Valley Duck | 0 | 1 |
| 2022.10.31 | Shandong | Duck | Cherry Valley Duck | 0 | 1 |
| 2022.10.31 | Shandong | Duck | Cherry Valley Duck | 0 | 1 |
| 2022.10.31 | Shandong | Duck | Cherry Valley Duck | 0 | 1 |
| 2022.10.31 | Shandong | Duck | Cherry Valley Duck | 0 | 1 |
| 2022.10.31 | Shandong | Duck | Cherry Valley Duck | 0 | 1 |
| 2022.10.31 | Shandong | Duck | Cherry Valley Duck | 0 | 1 |
| 2022.10.31 | Shandong | Duck | Cherry Valley Duck | 0 | 1 |
| 2022.10.31 | Shandong | Duck | Cherry Valley Duck | 0 | 1 |
| 2022.10.31 | Shandong | Duck | Cherry Valley Duck | 0 | 1 |
| 2022.10.31 | Shandong | Duck | Cherry Valley Duck | 0 | 1 |
| 2022.10.31 | Shandong | Duck | Cherry Valley Duck | 0 | 1 |
| 2022.10.31 | Shandong | Duck | Cherry Valley Duck | 0 | 1 |
| 2022.10.31 | Shandong | Duck | Cherry Valley Duck | 0 | 1 |
| 2022.10.31 | Shandong | Duck | Cherry Valley Duck | 0 | 1 |
| 2022.10.31 | Shandong | Duck | Cherry Valley Duck | 0 | 1 |
| 2022.10.31 | Shandong | Duck | Cherry Valley Duck | 0 | 1 |
| 2022.10.31 | Shandong | Duck | Cherry Valley Duck | 0 | 1 |
| 2022.10.31 | Shandong | Duck | Cherry Valley Duck | 28 | 1 |
| 2022.10.31 | Nei Mongol | Duck | Cherry Valley Duck | 270 | 1 |
| 2022.10.31 | Nei Mongol | Duck | Cherry Valley Duck | 270 | 1 |
| 2022.10.31 | Nei Mongol | Duck | Cherry Valley Duck | 270 | 1 |
| 2022.10.31 | Nei Mongol | Duck | Cherry Valley Duck | 270 | 1 |
| 2022.10.31 | Nei Mongol | Duck | Cherry Valley Duck | 270 | 1 |
| 2022.10.31 | Nei Mongol | Duck | Cherry Valley Duck | 270 | 1 |
| 2022.10.31 | Nei Mongol | Duck | Cherry Valley Duck | 270 | 1 |
| 2022.10.31 | Nei Mongol | Duck | Cherry Valley Duck | 270 | 1 |
| 2022.10.31 | Nei Mongol | Duck | Cherry Valley Duck | 270 | 1 |
| 2022.10.31 | Nei Mongol | Duck | Cherry Valley Duck | 270 | 1 |
| 2022.10.31 | Guangxi | Duck | / | 20 | 1 |
| 2022.10.31 | Guangxi | Duck | / | 20 | 1 |
| 2022.10.31 | Guangxi | Duck | / | 20 | 1 |
| 2022.10.31 | Guangxi | Duck | / | 20 | 1 |
| 2022.10.31 | Guangxi | Duck | / | 20 | 1 |
| 2022.10.5 | Henan | Duck | / | 39 | 1 |
| 2022.10.5 | Henan | Duck | / | 39 | 1 |
| 2022.10.5 | Shandong | Duck | / | 33 | 1 |
| 2022.10.5 | Shandong | Duck | / | 33 | 1 |
| 2022.3.11 | Shandong | Duck | / | 24 | 1 |
| 2022.3.11 | Shandong | Duck | / | 26 | 1 |
| 2022.3.11 | Shandong | Duck | / | 24 | 1 |
| 2022.3.14 | Shandong | Duck | / | 26 | 1 |
| 2022.3.15 | Shandong | Duck | / | 27 | 1 |
| 2022.3.16 | Shandong | Duck | / | 18 | 1 |
| 2022.3.24 | Shandong | Duck | / | 27 | 1 |
| 2022.3.27 | Shandong | Duck | / | 21 | 1 |
| 2022.3.27 | Shandong | Duck | / | 26 | 1 |
| 2022.3.30 | Shandong | Duck | / | 26 | 1 |
| 2022.3.4 | Shandong | Duck | / | 28 | 2 |
| 2022.3.6 | Shandong | Duck | / | 27 | 1 |
| 2022.3.8 | Shandong | Duck | / | 25 | 1 |
| 2022.3.9 | Shandong | Duck | / | 24 | 1 |
| 2022.4.10 | Shandong | Duck | / | 26 | 1 |
| 2022.4.11 | Shandong | Duck | / | 19 | 1 |
| 2022.4.17 | Shandong | Duck | / | 25 | 1 |
| 2022.4.21 | Shandong | Duck | / | 24 | 1 |
| 2022.4.23 | Shandong | Duck | / | 26 | 1 |
| 2022.4.24 | Shandong | Duck | / | 20 | 1 |
| 2022.4.26 | Shandong | Duck | / | 27 | 1 |
| 2022.5.12 | Shandong | Duck | / | 27 | 1 |
| 2022.5.15 | Shandong | Duck | / | 16 | 1 |
| 2022.5.15 | Shandong | Duck | / | 28 | 1 |
| 2022.5.16 | Shandong | Duck | / | 18 | 1 |
| 2022.5.16 | Shandong | Duck | / | 28 | 1 |
| 2022.5.18 | Shandong | Duck | / | 27 | 1 |
| 2022.5.22 | Shandong | Duck | / | 15 | 1 |
| 2022.5.22 | Shandong | Duck | / | 15 | 1 |
| 2022.5.26 | Shandong | Duck | / | 15 | 1 |
| 2022.5.28 | Shandong | Duck | / | 21 | 1 |
| 2022.5.6 | Shandong | Duck | / | 30 | 1 |
| 2022.5.8 | Shandong | Duck | / | 18 | 1 |
| 2022.6.1 | Shandong | Duck | / | 21 | 1 |
| 2022.6.1 | Shandong | Duck | / | 15 | 1 |
| 2022.6.10 | Shandong | Duck | / | 35 | 1 |
| 2022.6.12 | Henan | Duck | / | 29 | 1 |
| 2022.6.12 | Henan | Duck | / | 30 | 1 |
| 2022.6.12 | Henan | Duck | / | 17 | 1 |
| 2022.6.12 | Shandong | Duck | / | 22 | 1 |
| 2022.6.14 | Shandong | Duck | / | 30 | 1 |
| 2022.6.16 | Shandong | Duck | / | 34 | 1 |
| 2022.6.22 | Shandong | Duck | / | 34 | 1 |
| 2022.6.24 | Shandong | Duck | / | 33 | 1 |
| 2022.6.26 | Shandong | Duck | / | / | 7 |
| 2022.6.26 | Shandong | Duck | / | / | 3 |
| 2022.6.26 | Shandong | Duck | / | / | 1 |
| 2022.6.7 | Shandong | Duck | / | 18 | 1 |
| 2022.6.7 | Shandong | Duck | / | 26 | 1 |
| 2022.6.8 | Shandong | Duck | / | 27 | 1 |
| 2022.6.8 | Nei Mongol | Duck | / | 17 | 1 |
| 2022.6.8 | Nei Mongol | Duck | / | 17 | 1 |
| 2022.6.8 | Shandong | Duck | / | 35 | 1 |
| 2022.6.8 | Shandong | Duck | / | 35 | 1 |
| 2022.6.8 | Shandong | Duck | / | 35 | 1 |
| 2022.6.8 | Shandong | Duck | / | 20 | 1 |
| 2022.6.9 | Shandong | Duck | / | 34 | 1 |
| 2022.7.10 | Hebei | Duck | / | 36 | 1 |
| 2022.7.11 | Shandong | Duck | / | 36 | 1 |
| 2022.7.11 | Shandong | Duck | / | 36 | 1 |
| 2022.7.11 | Shandong | Duck | / | 37 | 1 |
| 2022.7.11 | Shandong | Duck | / | / | 1 |
| 2022.7.11 | Shandong | Duck | / | / | 1 |
| 2022.7.11 | Shandong | Duck | / | 34 | 1 |
| 2022.7.11 | Shandong | Duck | / | 34 | 1 |
| 2022.7.11 | Shandong | Duck | / | 34 | 1 |
| 2022.7.11 | Shandong | Duck | / | 34 | 1 |
| 2022.7.11 | Shandong | Duck | / | 34 | 1 |
| 2022.7.11 | Shandong | Duck | / | 34 | 1 |
| 2022.7.12 | Shandong | Duck | / | / | 1 |
| 2022.7.13 | Shandong | Duck | / | 25 | 1 |
| 2022.7.13 | Shandong | Duck | / | 84 | 1 |
| 2022.7.13 | Shandong | Duck | / | / | 1 |
| 2022.7.13 | Shandong | Duck | / | / | 1 |
| 2022.7.13 | Shandong | Duck | / | 37 | 1 |
| 2022.7.13 | Shandong | Duck | / | 5 | 1 |
| 2022.7.13 | Shandong | Duck | / | 5 | 1 |
| 2022.7.17 | Shandong | Duck | / | 25 | 1 |
| 2022.7.17 | Shandong | Duck | / | 33 | 1 |
| 2022.7.2 | Shandong | Duck | / | 31 | 1 |
| 2022.7.23 | Shandong | Duck | / | 22 | 1 |
| 2022.7.24 | Shandong | Duck | / | / | 1 |
| 2022.7.25 | Shandong | Duck | / | 21 | 1 |
| 2022.7.25 | Shandong | Duck | / | 31 | 1 |
| 2022.7.25 | Shandong | Duck | / | 28 | 1 |
| 2022.7.25 | Shandong | Duck | / | 30 | 1 |
| 2022.7.25 | Shandong | Duck | / | 30 | 1 |
| 2022.7.28 | Shandong | Duck | / | 24 | 1 |
| 2022.7.28 | Shandong | Duck | / | / | 1 |
| 2022.7.5 | Shandong | Duck | / | 24 | 1 |
| 2022.7.5 | Shandong | Duck | / | / | 1 |
| 2022.7.7 | Shandong | Duck | / | 14 | 1 |
| 2022.7.7 | Shandong | Duck | / | 26 | 1 |
| 2022.7.8 | Shandong | Duck | / | / | 2 |
| 2022.7.9 | Shandong | Duck | / | 33 | 1 |
| 2022.8.1 | Shandong | Duck | / | 36 | 1 |
| 2022.8.12 | Hebei | Duck | / | 38 | 1 |
| 2022.8.12 | Shandong | Duck | / | 28 | 1 |
| 2022.8.12 | Shandong | Duck | / | 26 | 1 |
| 2022.8.12 | Shandong | Duck | / | 36 | 1 |
| 2022.8.15 | Shandong | Duck | / | / | 1 |
| 2022.8.15 | Shandong | Duck | / | 31 | 1 |
| 2022.8.16 | Shandong | Duck | / | 16 | 1 |
| 2022.8.16 | Shandong | Duck | / | 38 | 1 |
| 2022.8.16 | Shandong | Duck | / | 22 | 1 |
| 2022.8.19 | Shandong | Duck | / | / | 1 |
| 2022.8.19 | Shandong | Duck | / | 32 | 1 |
| 2022.8.19 | Shandong | Duck | / | 32 | 1 |
| 2022.8.20 | Shandong | Duck | / | 25 | 1 |
| 2022.8.20 | Shandong | Duck | / | 30 | 1 |
| 2022.8.22 | Shandong | Duck | / | 20 | 1 |
| 2022.8.23 | Hebei | Duck | / | 28 | 1 |
| 2022.8.23 | Hebei | Duck | / | 37 | 1 |
| 2022.8.26 | Shandong | Duck | / | 22 | 1 |
| 2022.8.27 | Hebei | Duck | / | 33 | 1 |
| 2022.8.27 | Shandong | Duck | / | 30 | 1 |
| 2022.8.29 | Shandong | Duck | / | 36 | 1 |
| 2022.8.29 | Shandong | Duck | / | 29 | 1 |
| 2022.8.29 | Shandong | Duck | / | 21 | 1 |
| 2022.8.29 | Shandong | Duck | / | 20 | 1 |
| 2022.8.3 | Hebei | Duck | / | 38 | 1 |
| 2022.8.3 | Hebei | Duck | / | 38 | 1 |
| 2022.8.3 | Shandong | Duck | / | 29 | 1 |
| 2022.8.6 | Shandong | Duck | / | 30 | 1 |
| 2022.8.9 | Shandong | Duck | / | 26 | 1 |
| 2022.8.9 | Shandong | Duck | / | 20 | 1 |
| 2022.8.9 | Shandong | Duck | / | 36 | 1 |
| 2022.8.9 | Shandong | Duck | / | 21 | 1 |
| 2022.9.1 | Shandong | Duck | / | 36 | 1 |
| 2022.9.1 | Shandong | Duck | / | 30 | 1 |
| 2022.9.11 | Shandong | Duck | / | 18 | 1 |
| 2022.9.15 | Shandong | Duck | / | 31 | 1 |
| 2022.9.18 | Shandong | Duck | / | 36 | 1 |
| 2022.9.18 | Shandong | Duck | / | 34 | 1 |
| 2022.9.23 | Shandong | Duck | / | 32 | 1 |
| 2022.9.24 | Shandong | Duck | / | 39 | 1 |
| 2022.9.28 | Shandong | Duck | / | 28 | 1 |
| 2022.9.29 | Hebei | Duck | / | 33 | 1 |
| 2022.9.29 | Hebei | Duck | / | 33 | 1 |
| 2022.9.5 | Shandong | Duck | / | 35 | 1 |
| 2022.9.6 | Shandong | Duck | / | 15 | 1 |
| 2022.9.7 | Shandong | Duck | / | 13 | 1 |
| 2022.9.7 | Shandong | Duck | / | 19 | 1 |

The “/” indicates that the specific information of the item is unknown.
